# Apprehension and educational outcomes among Hispanic students in the United States: The impact of Secure Communities

**DOI:** 10.1371/journal.pone.0276636

**Published:** 2022-10-24

**Authors:** Rosa Weber

**Affiliations:** 1 Department of Sociology, Stockholm University, Stockholm, Sweden; 2 Institut National d’Études Démographiques, Paris, France; University of Milano–Bicocca: Universita degli Studi di Milano-Bicocca, ITALY

## Abstract

Prior research suggests that disruptive events, such as shocks induced by family instability, neighborhood violence, or relocation, tend to be detrimental for children’s educational outcomes, but findings are heterogeneous depending on the type of event. Limited evidence is available on how shocks resulting from immigration enforcement impact educational outcomes among targeted minority groups. This study contributes to the literature by assessing how a policy implementation in the US–Secure Communities–is related to the school district level achievement of Hispanic students. The Secure Communities program is a national level immigration enforcement policy that was rolled out on a county-by-county basis. The program has increased the risk of deportation and led to rising apprehension and insecurity among undocumented migrants and the wider Hispanic community. Using detailed information on the implementation of Secure Communities, data from the Stanford Education Data Archive, and the Current Population Survey, this study estimates dynamic difference in differences exploiting regional variation in the timing of the policy change to assess its impact on educational outcomes. Results show that the activation of Secure Communities is negatively associated with Hispanic students’ subsequent English language arts achievement, while white and black students’ achievement does not change. Findings further suggest that Hispanic students living in the South, rural areas, and areas with high proportions of likely undocumented migrants are disproportionately impacted by the program’s activation. Whereas, Hispanic students in sanctuary jurisdictions, which reduce the likelihood of deportation, are not impacted. These findings indicate that immigration enforcement can have negative consequences for educational and social inequalities in the United States.

## Introduction

A considerable literature studies the impact of disruptive events on children’s outcomes. Prior studies suggest that shocks are on average detrimental for children’s academic progress and social-psychological well-being, though effects vary depending on the type of shock. Local violence and family instability have been shown to impact children’s cognitive performance and educational achievement negatively [1–5]. In contrast, relocation to procure better school quality or policies that subsidize college education are generally achievement-enhancing [6, 7].

Some types of US immigration enforcement have been conceptualized as shocks to the wider Hispanic community [8, 9]. A number of recent studies show that immigration enforcement not only affects undocumented migrants through the direct risk of deportation [10–12], but also has unintended negative consequences for documented and US-born Hispanics [13–17]. While family ties to undocumented migrants have been linked to anxiety that a parent or sibling will be deported [16], statistical discrimination can lead to difficulties in securing work or stigmatization of the wider Hispanic community [18, 19]. As many as 5.1 million children under age 18 lived in mixed status households in the US during the period 2009–13 [20]. Considering the size of the population as well as dramatic increases in deportations observed over recent decades, it is important to gain a better understanding of how Hispanic children respond to immigration enforcement.

Prior studies have indicated that immigration enforcement can have negative consequences for Hispanic students’ educational outcomes [21, 22]. Bellows examined the relationship between Secure Communities (SC) and student achievement at the county level [23]. Analyses based on data from the Stanford Education Data Archive showed that the activation of SC was associated with decreases in Hispanic students’ English language arts achievement and black students’ English language arts and math achievement. In contrast, Kirksey and co-authors’ study using the same data found an association between deportations and the Hispanic-white educational achievement gap, but no association between deportations and the black-white achievement gap [24]. These divergent findings may be in part due to different samples and study designs, where the first study assesses county level achievement across the US [23], and the second study assesses school district level achievement gaps in cities which are within 100 miles of deportation sites [24]. How students across racial groups are impacted by immigration enforcement, and how they respond to them in their educational outcomes, requires further investigation.

This study builds on the prior literature in two ways. First, it exploits the staggered rollout of SC to estimate dynamic difference in differences on the impact of SC on school district level achievement (SDLA) of Hispanic, white, and black students using detailed information on the implementation and geographic scope of SC and data from the Stanford Education Data Archive (SEDA). It also runs a placebo test on sanctuary jurisdictions, which are cities and counties that ordained laws or regulations to block immigration enforcement. The previously described study by Bellows assessed educational outcomes at the county level [23]. While SC was implemented at the county level, there is considerable heterogeneity within counties. The US has about 3,240 counties, whereas there are more than 13,000 geographically defined public school districts. School districts, thus, provide a more disaggregated measure of test scores, as well as students’ demographic characteristics and socioeconomic background. Most school districts also have authority over public schools that are within their geographic boundaries and have been shown to differ in their approaches to school improvement, which can impact student learning through school district-wide reforms [25–27].

Second, this study assesses heterogeneity within the relationship between SC and Hispanic students’ educational achievement across different contexts. Even though SC was activated nation-wide, regional variation in attitudes toward immigrants and the size of the undocumented population likely shape how disruptive the policy is for Hispanics. For instance, roughly half of children under age 18 are estimated to be children of migrants in California, whereas the national share lies at 23 percent [28]. I therefore incorporate Current Population Census (CPS) data, which allow me to assess the likely undocumented population in the state. Using this information, I analyze differences in the impact of SC across areas with different shares of likely undocumented migrants, as well as census regions, and rural and urban areas. This can provide indirect evidence on the likely mechanisms underlying the impact of SC on Hispanic students’ educational outcomes.

Common perceptions about the implications of disruptive events, such as changed policy settings and sudden economic distress, differ by the underlying perspective. On the one hand, fear that a family member will be deported as well as reduced interaction with the wider society can lead to stress and impede English progress, negatively impacting educational performance [16, 29, 30]. On the other hand, children’s outcomes can also be positively affected. Considering that undocumented migrants face barriers to participating in the formal labor market and Hispanic students are more likely to encounter employer discrimination than other students, education can provide an important alternative to entering the labor market [7, 31, 32]. This may encourage Hispanic families to invest in their children’s human capital with the aim of facilitating the transition to upper secondary education.

Hispanic children, who are frequently subjected to shocks, evoke particular concern. Studies show that return and circular migration have become less common among Mexican migrants in the US, at least in part, because of rising deportations [33, 34]. During their longer stays in the US, many undocumented migrants form families north of the border [17, 35, 36]. Even though most children of undocumented migrants hold a US citizenship, they risk facing a combination of economic disadvantage, school instability, and discrimination [30, 37]. Early life adversities may accrue over the life course, for example due to group-level differences in the returns to socioeconomic resources, as hypothesized by the theory of cumulative disadvantage [38], or when certain outcomes are used as markers for subsequent opportunities, as the theory of cumulative advantage posits [39, 40]. These mechanisms have the potential to impede future labor market outcomes and can exacerbate social differences in the US [41].

In the following sections, I discuss the implementation of SC. Thereafter, I review the theoretical background and previous literature. Then, I describe the data, followed by the empirical strategy, and findings. Finally, I conclude and outline next steps for research.

## Secure Communities–A policy background

Federal initiatives, such as the SC program, have drawn state, county, and municipal police forces into the active enforcement of federal immigration laws. The SC program sought to increase the detention and deportation of immigrants who commit crimes in the US. Beginning in October 2008, SC created a screening process wherein every person arrested by local law enforcement officials in the US would automatically be cross-referenced with federal authorities for immigration status and deportation eligibility [42]. Previous programs required police officers or Immigration and Customs Enforcement (ICE) agents to interview each person individually to assess whether they were immigration violators. By automating the process, SC greatly increased the likelihood of detention. The fiscal year of 2012 marked a peak of more than 464,000 migrants cycling through detention facilities [43].

Prior studies show that the program has not impacted overall crime rates [44], but instead increased the likelihood that Hispanics are arrested and undocumented migrants deported [45]. Even though SC was directed at all migrants, the policy primarily targeted the Hispanic community, at least in part, due to differences in the share of the undocumented population across groups. Estimates based on the CPS indicate that about 23 percent of Hispanics age 20 and above are likely undocumented migrants. Among white and black persons, the respective shares are 5 and 2 percent.

Although SC marks the largest expansion of interior immigration enforcement in the US to date, deportations have risen consistently in bipartisan fashion since the early 1990s. Immigration enforcement previously focused on border crossings but has shifted to the US interior in more recent years. This is largely due to the decreasing number of undocumented migrants attempting to cross the border [33, 34]. Besides SC, a number of other immigration enforcement policies were implemented at the state and county-level during the 2000s. These included E-verify, Omnibus Immigration Laws, and 287(g) agreements, which enable employers, as well as local and state officials, to check employees’ migration status and to apprehend undocumented migrants. These policies have been implemented selectively and are not nearly as widespread as SC. Local values and political views have been found to influence which states and counties implement these policies [46].

**Fig 1:**
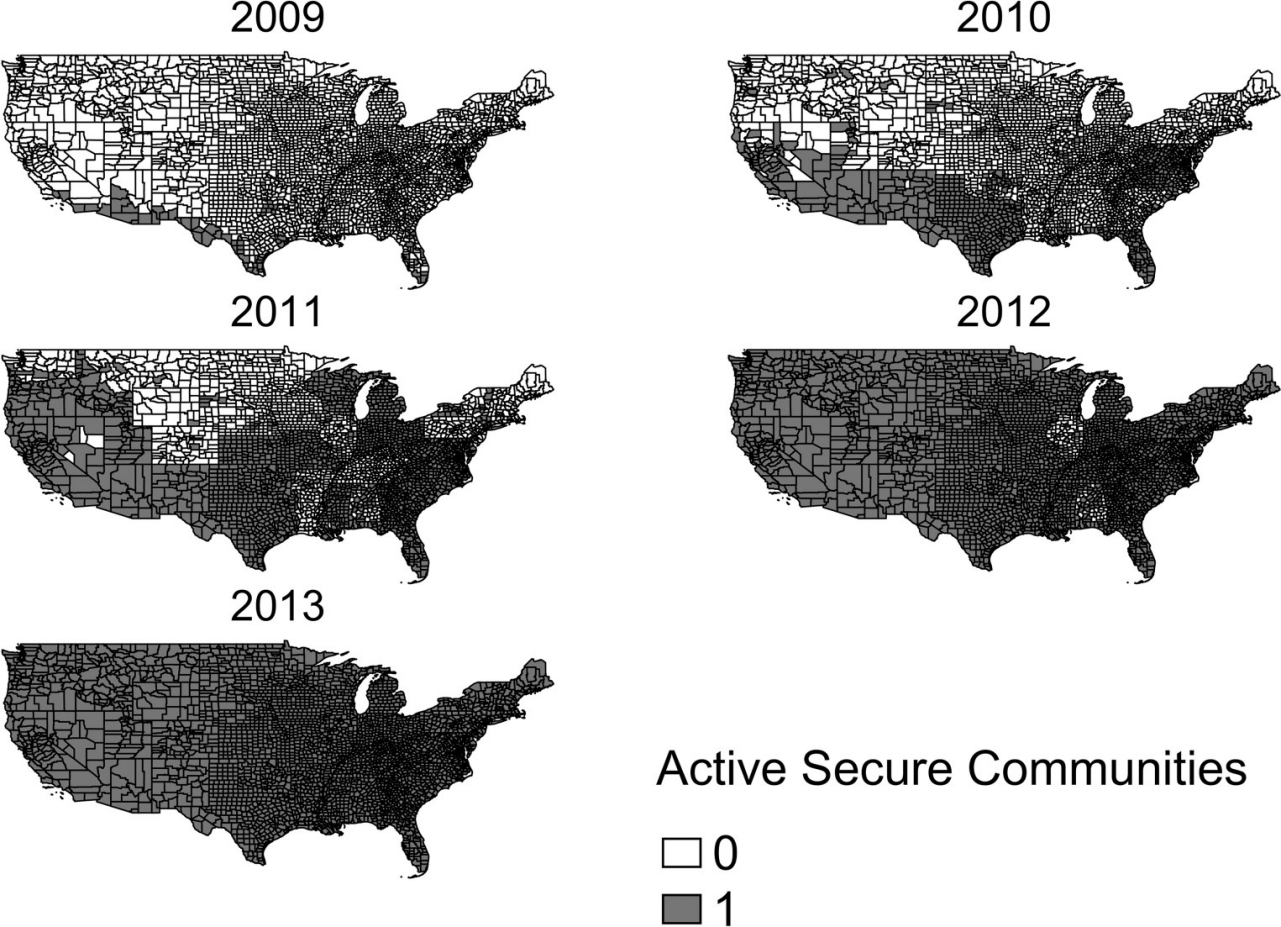
The rollout of Secure Communities across the United States. Data from the Department of Homeland Security (DHS). The tiger/line shapefile was obtained from the US Census Bureau website [48].

The scope of SC, resource bottlenecks, and technological constraints meant that the program could not be activated in the entire country at once. Consequently, the rollout was staggered over four years. The federal government determined the sequence of the rollout and implemented it on a county-by-county basis. Fig 1 shows that the program was first activated in counties located by the Mexico-US border. Prior work also reveals that the implementation of the program mirrored federal enforcement priorities for immigration [47]. By 2011, SC was implemented in roughly half the country and by January 2013 it was completely activated nation-wide. The federal government prohibited local governments from formally opting out of SC even though some counties did not want to participate. Once the screening system was in place, informal noncompliance was practically impossible [44].

Still, several cities and counties ordained laws or regulations that to this day block immigration enforcement and protect undocumented migrants from ICE. These are called sanctuary jurisdictions. A report based on data from the Transactional Records Access Clearinghouse (TRAC) at Syracuse University shows that sanctuary jurisdictions reduce the odds that undocumented migrants are arrested and deported by ICE. Despite official statements that ICE compensates for this by allocating more resources to communities where local jurisdictions fail to cooperate, the report does not find that the odds of other arrests, i.e., that are not linked to SC, are greater in sanctuary versus non-sanctuary jurisdictions [49].

Strong critique of SC started in Los Angeles and spread between 2012 and 2014. In November 2014, the program was officially discontinued. However, the Priority Enforcement Program directly replaced SC. The Priority Enforcement Program is in the same spirit as SC and relies on the same automated screening. Still, under this new program, ICE was instructed to transfer only information on severe criminal offenders and to request a detainer only if the person in custody was subject to a final order of removal or if there was other sufficient probable cause that the person was removable. The Trump administration subsequently re-labelled the Priority Enforcement Program as SC [50].

There are a number of educational reforms that were enacted simultaneously. The Deferred Action for Childhood Arrivals program was passed in 2012 and has been instrumental in providing undocumented migrants relief from deportation and work authorization if they are in education. Dream Acts have been passed in a number of states and allow undocumented students to pay in-state tuition at public higher education institutions [7]. In some states, including Texas, undocumented students can also apply for financial aid. Lastly, the No Child Left Behind Act administers standardized testing and tracks the progress of vulnerable student groups, such as migrant and minority groups. These reforms have provided undocumented migrants with opportunities to continue their education and increased the monitoring of low-performing groups.

## Previous research

Immigration enforcement influences the context that migrants live in and how they interact with society. The concept of racialized legal status has been used to describe social positions that are based on race-neutral legal classifications, such as documentation status, but which disproportionately impact racial minorities [8]. Racialized legal status directly affects undocumented migrants through economic distress [51, 52], detrimental health outcomes [10, 11, 13], and by exacerbating the psychosocial consequences of perceived stigma related to undocumented status in the US [53].

Racialized legal status has also been argued to impact documented migrants and US-born Hispanics indirectly. Such indirect effects are termed *chilling* effects referring to the fact that they arise from an icy policy climate rather than from a direct risk to the individual. In more general terms, chilling is used to describe a “discouraging or deterring effect, especially one resulting from a restrictive law or regulation” [54, 55]. In the context of welfare reform, chilling has been cited as a potential explanation for declines in program participation beyond what would be predicted due to eligibility changes alone [14, 15].

Chilling effects may operate through two mechanisms. The first mechanism hinges on social proximity to undocumented persons. For instance, children, who are born to undocumented parents in the US and are thus US citizens, fear that immigration enforcement will dissolve their families [16]. This increases the group’s anxiety levels and may have longer-term consequences for their academic progress [56]. The second mechanism operates through statistical discrimination. This impacts the wider Hispanic community, as employers or police officers racialize Hispanics by assuming that Hispanics, as a rule, are undocumented [19, 57, 58]. Investigatory traffic stops have been shown to disproportionately put Hispanics at risk of arrest and citation, independent of their legal status [9, 59]. Immigration policies may also incite anti-immigrant sentiments. For example, the enactment of anti-immigrant legislation in Arizona increased the frequency of negative tweets about migrants in that state [60]. These situations and the stress they trigger likely have negative consequences for educational performance.

Prior research further reveals that the way in which immigration enforcement impacts the wider Hispanic community depends on features of the local context. Considering regional differences in political values and attitudes toward immigrants, there is likely heterogeneity in the consequences of perceived stigma and discrimination of Hispanics across the US [61]. For instance, republican views have been shown to correlate with higher levels of deportation [46], which are overrepresented in more conservative Southern and mid-Western red states. In contrast, Northern, coastal states, and urban areas tend to be more politically liberal [62]. Similarly, residents in rural areas often have more restrictive views on immigration than residents in urban areas [63, 64]. While nearly 60 percent of residents in rural areas believe that the growing number of immigrants threatens traditional American customs and values, 35 percent of urban residents share this view [61]. In rural areas and small towns that have become new destinations to growing numbers of immigrants, Hispanics are also often met by negative stereotypes when speaking Spanish or gathering in public places [19].

Another feature of the local context that shapes the extent to which children are exposed to immigration enforcement is the size of the undocumented population in the area. For Hispanic children, who live in areas with large undocumented populations, immigration enforcement has a strong presence. In New Jersey, where the Mexican community is relatively large and established, deportations directly affect children’s neighbors and other community members. Seeing this, children try to predict the risk that their parents’ lack of documentation will lead to deportation. In contrast, in settings where the Mexican community is small, documentation status does not affect Hispanic children’s daily lives, unless the family experienced deportation directly [16]. Children are, however, aware that for many Americans ‘Mexican’ is synonymous with ‘illegal’ and describe being teased and excluded because of their racial background [16]. This suggests that Hispanics are impacted in both settings, but that the level of exposure to immigration enforcement is likely higher in areas with large undocumented populations.

Depending on the underlying perspective, shocks resulting from immigration enforcement may have different impacts on children’s and young adults’ educational outcomes. Immigration enforcement has been shown to augment economic insecurity [51, 65] and the frequency of relocation [66, 67], which can influence Hispanic students’ cognitive performance negatively [29, 30]. Reduced interaction with native-English speakers may, in particular, impact Hispanic students’ English language development [19, 53]. In line with this perspective, evidence based on the CPS reveals that intensified enforcement raises the probability of repeating a grade by 14 percent for children aged 6–13, whose parents are assumed to be undocumented. The likelihood of dropping out of school also increases by 18 percent for those aged 14–17 [21, 22]. The previously mentioned study by Bellows reveals a negative relationship between SC and county level achievement among Hispanic and black students [23]. Whereas, Kirksey and co-authors find that deportations are related to the Hispanic-white school district level achievement gap, but not to the black-white achievement gap [24].

However, immigration enforcement could potentially also have a positive impact on educational outcomes. In response to policy shifts, undocumented migrants’ labor market position has become increasingly tenuous since the mid-1980s [32, 68]. For many undocumented students, upper secondary education has therefore come to represent an escape from menial jobs when finishing school [7, 31]. Policy shifts have also changed how employers treat Hispanic workers in general [69]. Seeing that greater enforcement means more penalties related to the hiring of undocumented migrants, employers increasingly outsource the hiring of migrant workers to avoid federal sanctions. Employers have also started to verify the legal documents of all persons who they suspect to be foreign-born [57]. In other words, immigration enforcement has increased employer screening and in turn often augmented racial discrimination. Lower employment opportunities and discrimination resulting from immigration enforcement may then incentivize Hispanic families to invest in the human capital of their children, independent of their documentation status. This can lead Hispanic students to perform better in school, as they aspire to stay in education rather than entering the labor market where they face fewer opportunities.

This study contributes to the literature by assessing the potential positive and negative impacts of SC on Hispanic, white, and black students’ educational outcomes at the school district level, and by showing how these vary across census regions, rural and urban areas, and areas with different proportions of likely undocumented migrants. Since the policy implementation targeted the Hispanic community and white and black students are less likely to be in close social proximity to undocumented migrants, I do not expect to observe sizeable changes in the educational outcomes of white and black students.

## Data

First, I obtain publicly available data from the Department of Homeland Security (DHS) that provide the month and year in which SC was implemented for each county 2008–12 [70, 71]. I use these data to define the treatment status, coding the academic year in which SC was implemented in the county and subsequent years when the policy was in place as one, otherwise the indicator variable is coded as zero.

Second, I link these data using state ID, county name, and academic year to the SEDA data, which are publicly available aggregate data [72]. Specifically, the data provide annual measures of English language arts and math achievement for Hispanic, white, and black students over a set of different geographic units (geographically defined school districts, counties, metropolitan areas, commuting zones, and states) for each year 2009–18 and for each grade 3–8. Year refers to the spring of each academic year throughout the paper, so 2009 represents the 2008–9 academic year. Given that the DHS provides the month and year of the implementation of SC, the indicator variable shifts to one in the academic year of the policy activation. As an example, the indicator shifts to one in the academic year of 2010, if SC was implemented between July 2009 and June 2010.

The SEDA data uniquely identify schools by defining stable school identifiers and assign schools to larger geographic units. The stable school identifiers are based on *Longitudinal ID Crosswalks* provided in the Common Core of Data and enable tracking schools as they change school ID or school district. School district changes can be due to school districts splitting, merging, or some other administrative change [72, 73]. To define school districts, the SEDA data use the *2019 EDGE Unified and Elementary School District Boundaries* [74]. The US has more than 13,000 geographically defined public school districts. The structure of school districts varies by state and region. For instance, school districts in the Mid-Atlantic and New England states tend to follow county, township, or city boundaries, while school districts in the Midwest and Western states are generally independent of other municipal boundaries [75]. The total population size also varies across school districts. While the total population of the Berkeley Unified School District in California amounts to over 120,000 individuals, the Cochise Elementary District in Arizona comprises about 300 [76].

Most public-school districts have administrative control over the public schools that fall within their specific geographic boundaries. For schools that are physically located within a school district but not under its administrative control, such as charter and magnet schools, the SEDA data use information on location (latitude and longitude coordinates), school type, and school status to assign them to school districts [72]. This assignment ensures that school district level achievement reflects most of the students living in the school district. It also provides better alignment between achievement at the school district level and students’ demographic characteristics and socioeconomic background. These are constructed using information from the American Community Survey and reported for all families living in the geographic boundaries of the school district.

To assign schools to counties, the SEDA data use the county code provided in the Common Core of Data in the most recent year the school was observed, meaning that it is also stable over time. While most school districts are in one county, five percent of school districts belong to multiple counties. I assign each school district, which is in multiple counties, to the first recorded county. This is documented in S1 Table, where *County ID new* and *County name new* indicate the new assignment. The last column in S1 Table shows that 76 percent of school districts, which are in multiple counties, are in counties that activated SC in the same year (indicated by zero). The treatment assignment may therefore be incorrect for no more than 164 school districts. I ran sensitivity analyses dropping these school districts finding similar patterns to those presented in the main analysis. The results and are provided in S1 Fig.

I analyze school district level achievement aggregated across grades 3–8 by race and subject. The SEDA data are constructed using information on standardized tests that each state in the US is required to administer in public schools. States have flexibility in designing and administering the test. States also set their own standards regarding the proficiency level in each grade and subject. In order to improve comparability, the SEDA data transform SDLA into a common national scale based on overall state performance and on school district performance on the state exam relative to other school districts in the state [77]. Specifically, the cohort standardized scale is standardized within subject and grade, relative to the average of the four cohorts in the data who were in 4th grade in 2009, 2011, 2013, and 2015. Standardized school district means have an overall average near zero and a standard deviation of one. For example, a school district with a mean of 0.5 on the cohort standardized scale represents a school district where the average student scored one half of a standard deviation higher than the national reference cohort in the same grade [72, 78].

The SEDA data provide SDLA for each school district, grade, year, and subject in which there are at least 20 students in each cell or group (i.e., at least 20 Hispanic students in each grade, year, and subject within a school district). Values for observations with less than the required sample size are suppressed for privacy reasons [72]. In addition, SEDA data omit observations in which: the state test participation rate is less than 95 percent; students in the same state, grade, year, and subject took different tests; or the state did not report sufficient data. Overall, SEDA omits 10.9 percent of school district-grade-year-subject observations [72].

The SEDA data also provide information on annual school district-level characteristics including student demographics, which include the proportion of students who: qualify for free or reduced-price lunch; are in Special Education; and are English Language Learners. A socio-economic composite score combines information on median family income, household poverty rate, the proportion of adults who have a bachelor’s degree or higher, the proportion of adults who are unemployed, the proportion of households receiving SNAP benefits, and the proportion of households with children that are headed by a single mother. Additional school district characteristics include the proportion of students who are in city/urban, suburban, town, and rural locale schools. For more detailed information, see the Stanford Education Data Archive technical report under https://stacks.stanford.edu/file/druid:db586ns4974/seda_documentation_4.1.pdf.

Third, I link these data at the state level to the CPS for the years 2009–18 [79]. The CPS is collected by the US Census Bureau and has been approved by the Office of Management and Budget (OMB Number 0607–0049). All personally identifiable information is replaced by one or more artificial identifiers, or pseudonyms before the data are made publicly available. For more information, see https://www.census.gov/programs-surveys/cps/about.html. The CPS does not contain information on the documentation status of foreign-born, but following an existing literature I am able to identify probabilistically undocumented migrants among the adult population [80, 81]. In particular, I define an individual as probabilistically undocumented if the person is a foreign-born, non-veteran with no post-secondary education. I find that 26.6 percent of the foreign-born population age 20 and above are likely undocumented migrants, which is in line with prior estimates [82]. Among foreign-born Hispanics, about 45 percent are undocumented [83].

### Sample restrictions

I make some sample restrictions that are summarized in Table 1. The SEDA data suppress test scores by race where fewer than 20 students took the test, the participation rate was below 95 percent, students took different tests, or states did not report sufficient data [72]. Missing values are more common among Hispanic and black than white students. I also exclude Alaska and Hawaii, as well as early adopters from the sample, considering that the first counties that implemented SC substantially differ in economic and demographic composition from other counties. This is in line with previous research [14, 84]. In a last step, I exclude school districts without recorded scores for Hispanic students. I do this in order to estimate the coefficients for Hispanic, white, and black students using comparable samples. Considering that white students are observed roughly three times as often as Hispanic and black students, comparing estimates across models would otherwise imply comparisons of different samples. Results are robust to alternative sample restrictions, for instance keeping only school districts where we observe all three races, or excluding school districts where only white students’ scores are recorded. The analytical sample for English language arts consists of 29,735 school district-year observations for Hispanic students. The respective numbers are 27,500 for white students, and 15,078 for black students. For Math, it consists of 29,679 school district-year observations for Hispanic students. Again, the respective numbers are 27,394 for white students, and 14,925 for black students.

**Table 1:** Sample restrictions for the SEDA standardized achievement scores datafile.

A. Hispanic students	School district-grade-subject-years	School district-years	School districts	County	State
SEDA Standardized Achievement Scores data file	1,293,318	117,148	12,801	3,113	51
1. Exclude observations with missing achievement scores	383,583	34,775	4,482	1,576	50
2. Exclude Alaska and Hawaii	383,031	34,729	4,475	1,569	48
3. Exclude counties that were early adopters	333,782	30,129	3,966	1,488	48
4. Exclude school districts without recorded scores for Hispanic students	333,782	30,129	3,966	1,488	48
English language arts	172,748	**29,735**	3,945	1,479	48
Math	161,034	**29,679**	3,925	1,480	48
B. White students	School district-grade-subject-years	School district-years	School districts	County	State
SEDA Standardized Achievement Scores data file	1,293,318	117,148	12,801	3,113	51
1. Exclude observations with missing achievement scores	1,079,256	95,708	10,924	2,967	51
2. Exclude Alaska and Hawaii	1,077,878	95,589	10,905	2,951	49
3. Exclude counties that were early adopters	1,033,360	91,468	10,449	2,881	49
4. Exclude school districts without recorded scores for Hispanic students	310,015	27,846	3,727	1,465	48
English language arts	160,278	**27,500**	3,711	1,457	48
Math	149,737	**27,394**	3,692	1,458	48
C. Black students	School district-grade-subject-years	School district-years	School districts	County	State
SEDA Standardized Achievement Scores data file	1,293,318	117,148	12,801	3,113	51
1. Exclude observations with missing achievement scores	293,283	26,076	3,144	1,387	51
2. Exclude Alaska and Hawaii	292,923	26,046	3,140	1,383	49
3. Exclude counties that were early adopters	264,875	23,470	2,847	1,326	49
4. Exclude school districts without recorded scores for Hispanic students	171,326	15,244	2,042	938	48
English language arts	88,451	**15,078**	2,030	931	48
Math	82,875	**14,925**	2,017	931	48

Early adopters refer to school districts that implemented SC before the fall semester 2009.

Table 2 provides mean values for school districts in the years before and after the implementation of SC. Hispanic students’ SDLA in English language arts is about a third of a standard deviation below the national reference cohort (with a mean of -0.342 in years prior to SC and -0.260 in years after). In math, Hispanic students score about one fourth of a standard deviation lower than the national reference cohort indicated by mean values of -0.281 in years before the implementation of the program and -0.256 in years after. In both subjects, we observe improvements in SDLA over the time period analyzed.

**Table 2:** Descriptive statistics comparing years before and after Secure Communities.

Variable	Pre-Secure Communities	Post-Secure Communities	t-statistic
Hispanic English language arts	-0.342	-0.260	18.815
	(0.303)	(0.313)	
Hispanic Math	-0.281	-0.256	6.079
	(0.293)	(0.312)	
White English language arts	0.162	0.204	9.741
	(0.282)	(0.305)	
White Math	0.173	0.183	2.098
	(0.305)	(0.330)	
Black English language arts	-0.383	-0.380	0.606
	(0.268)	(0.284)	
Black Math	-0.424	-0.458	-6.379
	(0.260)	(0.296)	
% Free/reduced lunch	0.498	0.538	12.456
	(0.230)	(0.233)	
% Special education	0.133	0.128	-8.247
	(0.049)	(0.047)	
% English language learner	0.091	0.093	1.477
	(0.111)	(0.107)	
SES composite score	0.232	0.094	-11.280
	(0.846)	(0.929)	

Data from SEDA 2009–18 and DHS. The year of implementation is defined as Post-Secure Communities.

White students perform better in English language arts and math than the national reference cohort (0.162 in English language arts and 0.173 in math in years prior to SC). When comparing years before and after the implementation of SC, we also observe an increase in SDLA. Black students perform considerably lower than the national reference cohort (-0.383 in English language arts and -0.424 in math in years prior to SC). Among black students, we observe no improvement in SDLA over the observation period.

## Empirical strategy

The first part of the analysis estimates the impact of SC by comparing changes in the SDLA of Hispanic students in adopting school districts with contemporaneous changes in school districts that had not yet implemented SC. I estimate the following difference in differences specification:

$$Y_{dt}=\alpha +\beta I_{dt}^{post}+{X\prime }_{dt}\gamma +\mu_{d}+\rho_{t}+\epsilon_{dt},$$ (1)

where *Y*_*dt*_ stands for the dependent variable (i.e., English language arts or math SDLA among Hispanic students). Equivalent equations are estimated for white and black students. The suffix *d* stands for school district and *t* represents the year. The indicator of interest is $I_{dt}^{post}$. It is equal to one in all school district-years after the activation of SC. *X*′ is a vector of covariates that vary between school districts and over time. They include other immigration enforcement policies, a binary indicator of sanctuary jurisdictions, as well as the proportion of students who qualify for free or reduced-price lunch, are in Special Education, and are English Language Learners. Additional controls include a socioeconomic composite score. I control for these characteristics as they are determinants of SDLA [85, 86]. The specification also includes school district fixed effects (*μ*_*d*_) to account for any unobserved time-invariant school district-level factors that affect test scores, and year fixed effects (*ρ*_*t*_) to account for nation-wide policies or economic shocks that might influence SDLA, such as the enactment of the Deferred Action for Childhood Arrivals program or the No Child Left Behind Act. The models are weighted using precision weighting, which is the inverse of the standard error of SDLA squared.

I use two alternative approaches to estimate Eq 1, which are standard two-way fixed effects specifications and the interaction weighted estimation method proposed by Sun and Abraham [87]. This method provides estimates that are robust to treatment effect heterogeneity across cohorts. To estimate parameters following the robust approach, I first define cohorts of school districts that implemented SC in a given year. Second, I estimate cohort-specific average treatment effects using a two-way fixed effects specification that interacts relative period indicators with cohort indicators. Third, I calculate the weights for each relative time period using the sample shares of each cohort in the given period. Fourth, I combine the cohort-specific average treatment effect estimates and the weights to calculate the “interaction-weighted” estimate.

The main assumption underlying the empirical identification strategy is that without treatment time trends group specific outcomes would develop in parallel [88]. Considering that SC was implemented in a nation-wide rollout, school districts are classified as treated once SC has been implemented. Under the parallel trend assumption, comparing Hispanic students’ achievement in school districts that have implemented SC with that in school districts that have not yet implemented SC provides an estimate of the causal effect of SC on the change in Hispanic students’ SDLA [89]. Estimates for white and black students are interpreted in an analogous fashion. Considering that I cannot formally test the identification assumption, I plot pre- and post-trends in test scores to assess whether trends are parallel. Given the staggered rollout of SC, I have more information from years prior to the activation of SC for some counties than for others. Still, the SEDA data provide more limited information from years prior to the implementation. The dynamic difference in differences model underlying the method by Sun and Abraham is given by the following equation:

$$Y_{dt}=\alpha +\sum_{\tau =2}^{4}\delta_{-\tau }I_{d,t-\tau }^{pre}+\sum_{\tau =0}^{7}\delta_{+\tau }I_{d,t+\tau }^{post}+{X\prime }_{dt}\gamma +\mu_{d}+\rho_{t}+\epsilon_{dt},$$ (2)

where *Y*_*dt*_ stands for the dependent variable (i.e., English language arts or math SDLA among Hispanic students). Equivalent models are estimated for white and black students. The suffix *d* stands for school district and *t* represents the year. The coefficient *δ*_−*τ*_ identifies the impact of SC *τ* years prior to the implementation relative to the reference category of one year prior to the implementation of SC. We observe school districts up to four years prior to the implementation of SC. The coefficient *δ*_+*τ*_ identifies the impact of SC *τ* years after to the implementation and follows school districts up to seven years after the implementation. *X*′ is a vector of covariates that varies at the school district level and over time. It includes the same set of controls as provided in Eq 1. The specification also includes school district fixed effects (*μ*_*d*_) and year fixed effects (*ρ*_*t*_). Models are weighted using precision weighting.

A threat to the difference in differences assumptions is that the groups respond to the announcement of treatment. For instance, Hispanic families may relocate in response to the implementation of SC. Previous research shows that Hispanic families relocate to another US state when local immigration enforcement increases [66, 67]. However, considering that the SC program was implemented nation-wide, relocation because of it is not expected. S2 Table also shows that the proportion of Hispanic students in the school district does not change as a result of the program’s activation. This suggests that Hispanic families did not necessarily relocate to other parts of the US in response to SC.

My second approach is to focus on Hispanic students’ SDLA and to estimate stratified models across census regions, rural and urban areas, and the proportion of likely undocumented migrants in the state. As in the approach described above, this analysis builds on difference in differences models and is based on the specification in Eq 1. Results are obtained using the Sun and Abraham method. However, rather than analyzing the entire US, this approach assesses whether there is heterogeneity in the impact of SC across local contexts. First, information on the state of residence allows me to differentiate between four census regions: Northeast, Midwest, South, and West. Second, I use information from the SEDA data on the proportion of students in the school district who are in urban, suburban, town, or rural locale schools. Third, I incorporate data from the CPS and calculate quartiles on the proportion of likely undocumented migrants at the state-level. These analyses also include a placebo test, assessing educational outcomes among Hispanic students in sanctuary jurisdictions, where the activation of SC had a reduced impact.

## Empirical findings

### Difference in differences

**Fig 2:**
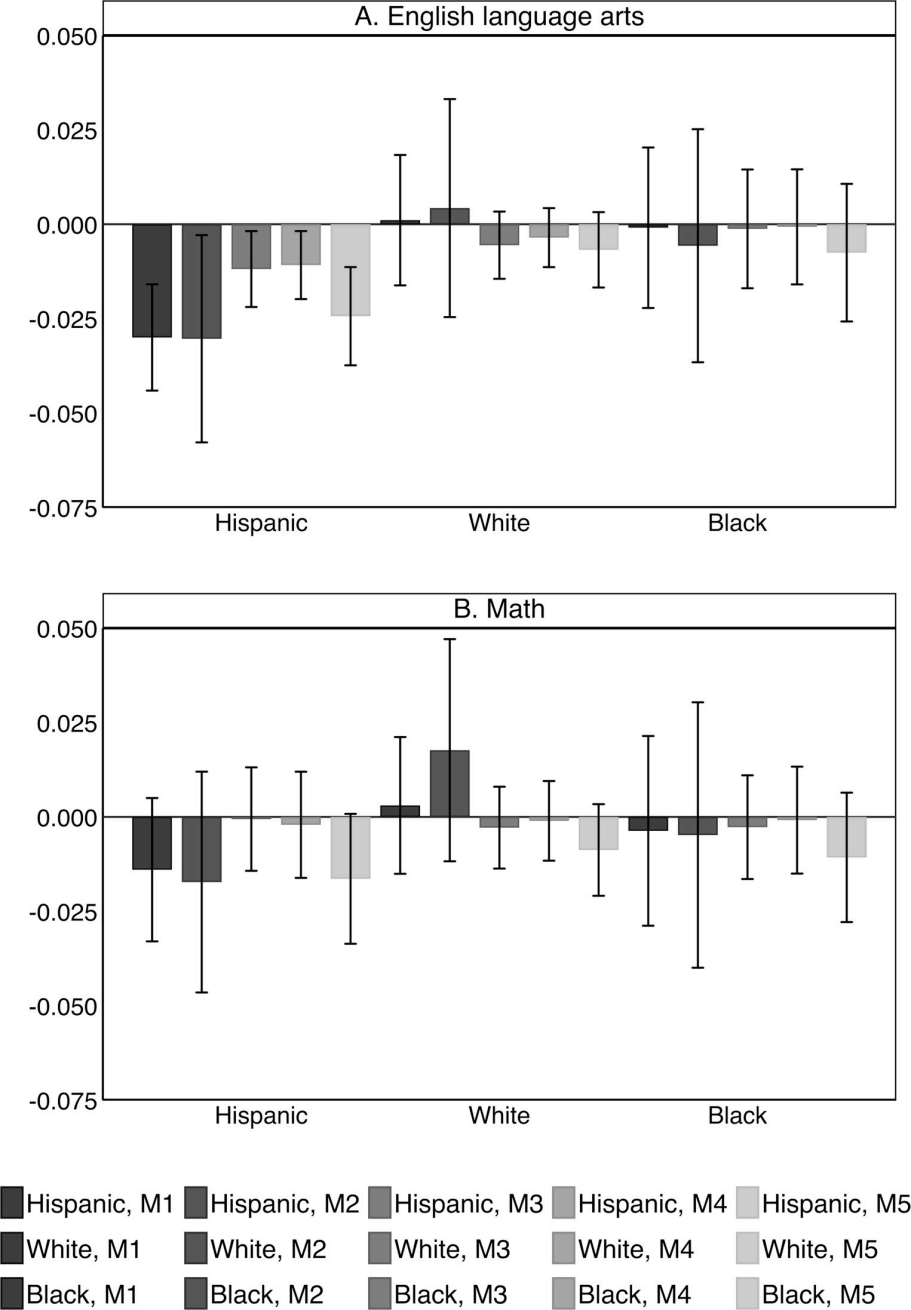
Estimated associations between the implementation of Secure Communities and school district level achievement among Hispanic, white, and black students. Data from SEDA 2009–18 and DHS. Coefficients and 95 percent confidence intervals based on Eq 1 are plotted. Models 1–4 show results obtained from standard two-way fixed effect specifications. Model 5 shows results obtained using the method outlined by Sun and Abraham (2021). The y-axis reports the size of the coefficients for Secure Communities and the x-axis reports race. Models are estimated with clustered standard errors at the county level and precision weights.

I begin by assessing the impact of SC on Hispanic, white, and black students’ test scores in the entire US. Fig 2 shows results from difference in differences models based on Eq 1. The models are estimated separately for Hispanic, white, and black students, and for test scores in English language arts and math. Each point estimate reports the impact of SC by comparing changes in the SDLA of adopting school districts with contemporaneous changes in school districts that had not yet implemented the program. Five models with different sets of fixed effects are presented. The first model includes year and state fixed effects and the second model adds state-by-year fixed effects. The third model includes year and county fixed effects and the fourth model includes year and school district fixed effects. The abovementioned models are estimated using standard two-way fixed effects specifications. The fifth model is estimated using the Sun and Abraham method and includes year and school district fixed effects. All models control for other policies that were implemented over the time period analyzed. These include E-verify, Omnibus Immigration Laws, state and county-level 287(g) agreements, and sanctuary jurisdictions. The presented models also account for school district level controls, which refer to the proportion of students who have free or reduced-price lunch, are in Special Education, are English Language Learners, and the SES composite score. Corresponding estimates and standard errors are provided in S3 and S4 Tables.

The estimate for SC is negative for Hispanic students’ SDLA in English language arts. In the first two models, we observe an estimate of -0.030. Introducing county fixed effects in model 3, reduces the estimate to -0.012 likely due to the fact that unobserved characteristics at the county level account for part of the association. The estimate remains similar in size in model 4, which further introduces school district fixed effects. Model 5 also includes school district fixed effects and accounts for potential heterogeneity in the treatment effect across cohorts using the Sun and Abraham method. The estimate indicates a change of -0.024 in English language arts SDLA. Among white and black students, there is no significant decline in English language arts achievement following the implementation of SC. For math, we observe no relationship between the implementation of SC and Hispanic, white, or black students’ achievement. The estimate for Hispanic students is negative but non-significant.

Regarding the size of the coefficient, Hispanic students’ SDLA in English language arts increased from -0.342 in years prior to the program to -0.260 in years after the implementation (Table 2). This indicates a nation-wide increase of 0.082 points. The negative estimate of SC suggests that the program led to a reduced improvement of Hispanic students’ SDLA. In relation to the increase of 0.082, the estimated coefficient of SC at -0.024 within school districts is non-trivial.

**Fig 3:**
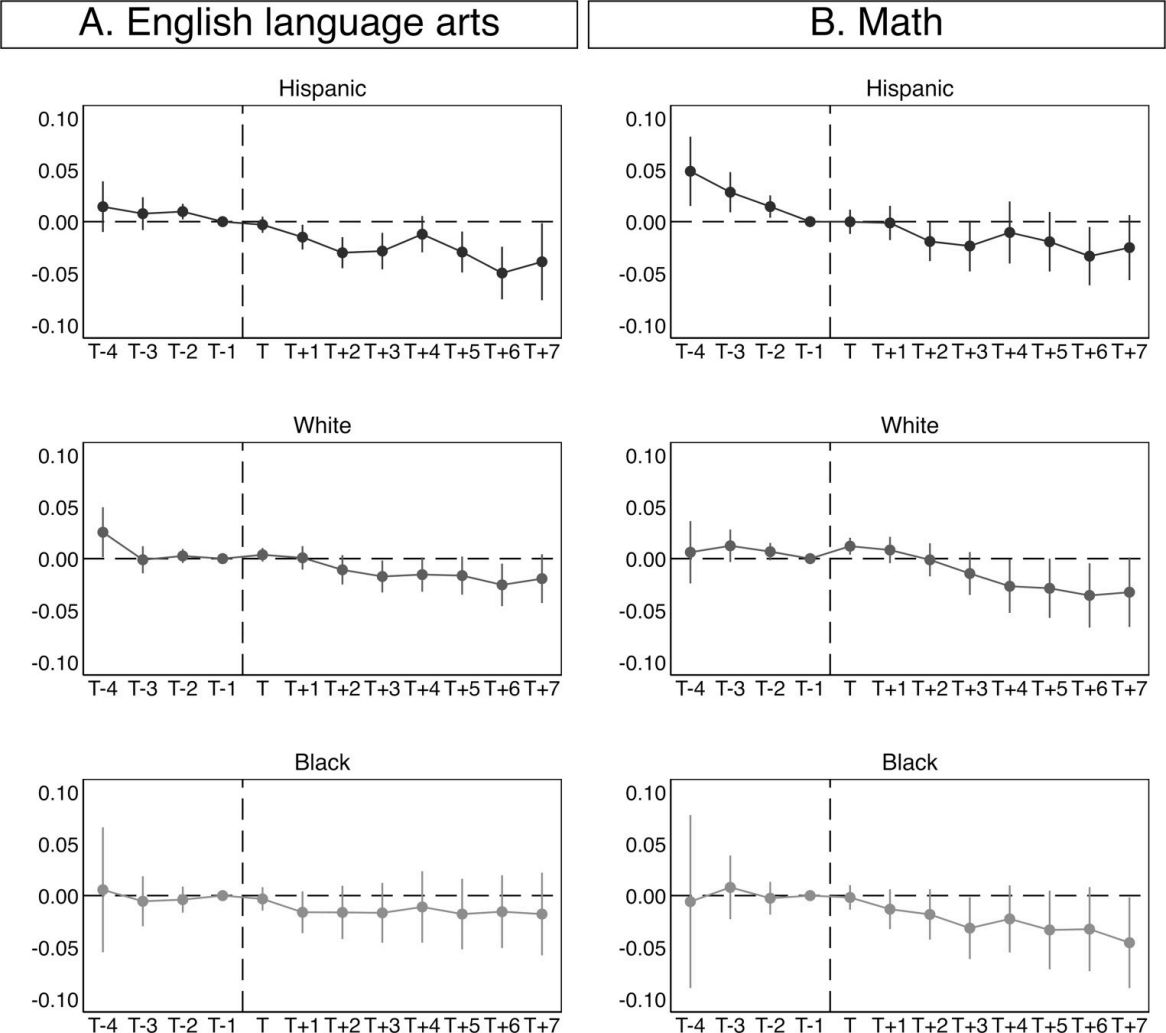
Estimated associations between the implementation of Secure Communities and school district level achievement among Hispanic, white, and black students relative to the implementation year. Data from SEDA 2009–18 and DHS. Coefficients and 95 percent confidence intervals based on Eq 2 are plotted. Results are obtained using the method outlined by Sun and Abraham (2021). The y-axis reports the size of the coefficients for Secure Communities and the x-axis the year relative to the implementation. T stands for the year of the implementation. Models are estimated with clustered standard errors at the county level and precision weights.

Fig 3 provides pre- and post-trends for the difference in English language arts and math scores between treated versus control school districts based on Eq 2. The x-axis shows years in relation to the implementation of SC. Four years of lead and seven years of lag are included. T stands for the year of the implementation. In year T, SC has been activated, while it has not yet been activated in year T-1. Non-significant trends in SDLA in years prior to the adoption of SC indicate parallel trends before the policy implementation and are used to assess the parallel trends assumption. The models are estimated separately for Hispanic, white, and black students, and for test scores in English language arts and math. They include school district and year fixed effects and are estimated using the Sun and Abraham method. The models further include the same set of control variables as those presented in Fig 2 (other policies and school district level controls). Corresponding estimates and standard errors are provided in S5 Table. S6 Table additionally provides weights for each relative time period obtained in the Sun and Abraham method.

In Fig 3, panel A shows that Hispanic students’ English language arts scores are similar in all years prior to the implementation of SC. These estimates indicate a stable pre-trend. After the implementation of the program, we observe a decline in SDLA. The impact of SC becomes stronger in the first years after implementation and then stabilizes, which is plausible considering that the policy may progressively expose children to stress as they gain awareness about its consequences before the impact stabilizes. Among white and black students, we observe no sizeable change in SDLA relative to the policy implementation.

Panel B shows that math scores decline prior to the activation of SC among Hispanic students. This indicates that the parallel trends assumption does not hold for math. Among white students, we observe relatively high math scores in the first years after the policy implementation. Subsequently, point estimates indicate decreasing scores but confidence intervals become quite large. Among black students, we do not observe a substantial decrease in math scores after the activation of SC. Taken together, the results presented in Fig 3 indicate that the parallel trends assumption does not hold for math, but roughly holds for Hispanic students in English language arts. Still, we need to be cautious when interpreting the coefficients as causal estimates.

### Stratified analyses

We assume that Hispanic students are more exposed to SC, because they are more likely to be in close social proximity to undocumented migrants and to experience statistical discrimination than white and black students. However, considering North-South and rural-urban divides in anti-immigrant sentiments and political values, it may be that Hispanic students are more strongly impacted in the South and rural areas. In contrast, sanctuary jurisdictions reduce the likelihood that undocumented migrants are arrested and deported by ICE. Hispanic students in these cities and counties are, thus, expected to be less exposed to SC.

Due to variation in the undocumented population, we further expect that Hispanic students are disproportionately impacted by immigration enforcement in states with high proportions of undocumented migrants in the adult population. In the following, I incorporate information from the CPS to calculate the proportion of likely undocumented migrants in the adult population age 20 and above at the state level between the years 2009 and 2018.

**Fig 4:**
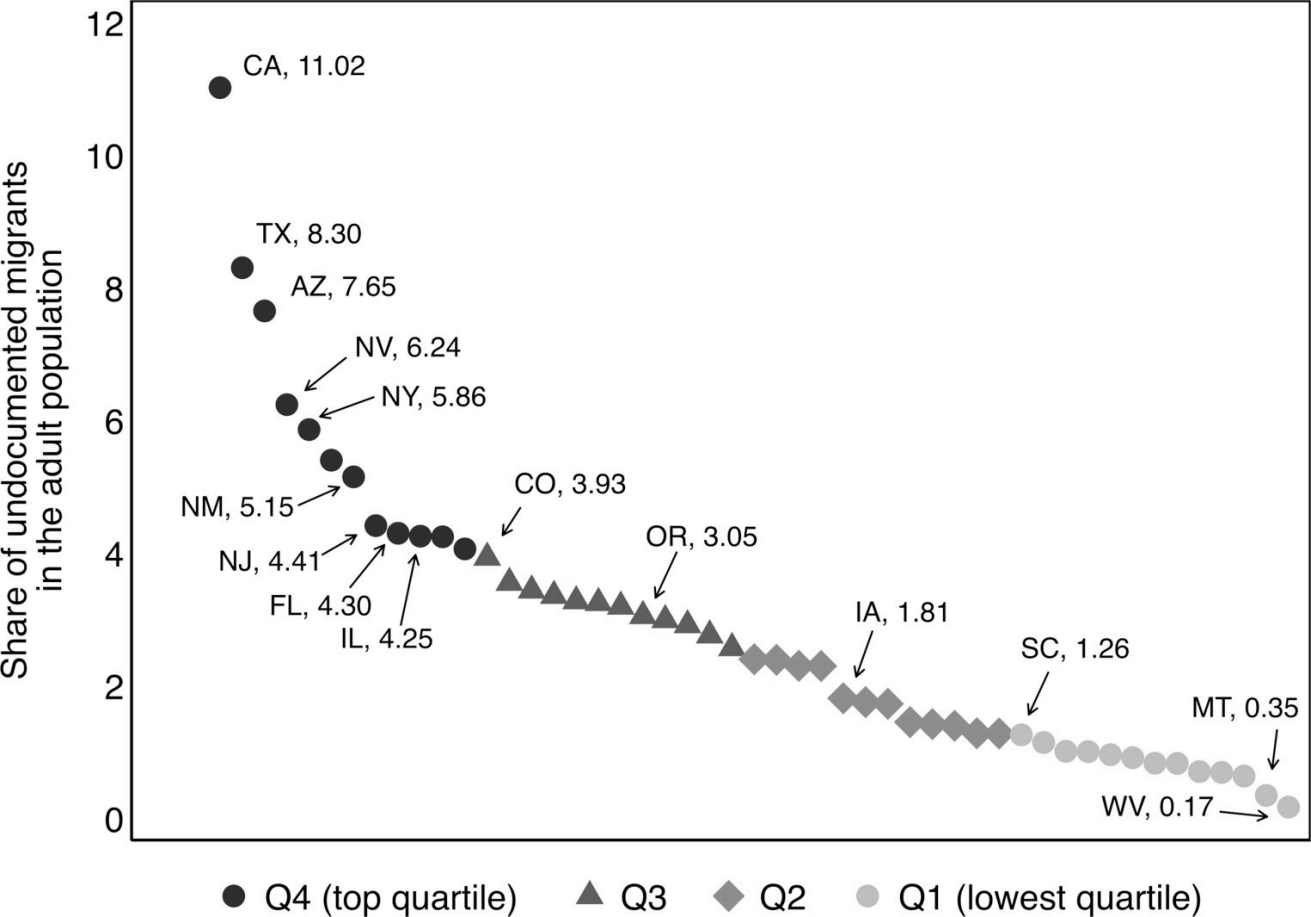
The proportion of likely undocumented in the state. Data from the CPS 2009–18.

Fig 4 provides a scatterplot of the share of undocumented migrants in the adult population based on the CPS data on the y-axis. The figure reveals considerable variation in the proportion of undocumented migrants across states. For instance, the proportion undocumented is highest in California where 11 percent of the adult population are likely undocumented migrants, followed by Texas and Arizona with 8 percent. The lowest proportions are observed in states, such as West Virginia and Montana with 0.17 and 0.35 percent, respectively.

I calculate quartiles where the top quartile comprises states with the highest proportion undocumented (indicated by dark gray circles) and the bottom quartile comprises states with the lowest proportion undocumented between 2009 and 2018 (indicated by light gray circles).

**Fig 5:**
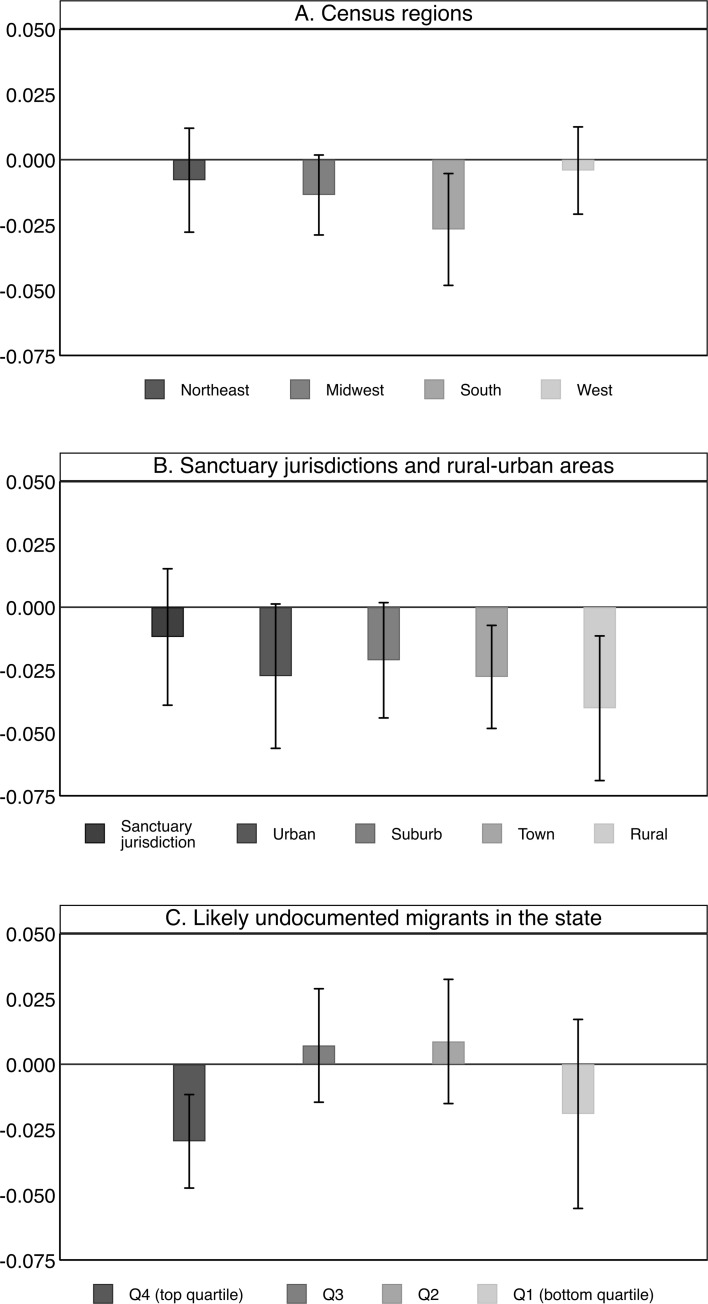
Estimated associations between the implementation of Secure Communities and school district level English language arts achievement among Hispanic students across census regions, rural-urban areas, and the proportion of likely undocumented migrants. Data from SEDA 2009–18, DHS, and CPS. Coefficients and 95 percent confidence intervals based on Eq 1 are plotted. Results are obtained using the method outlined by Sun and Abraham (2021). The y-axis reports the size of the coefficients for Secure Communities. Models are estimated with clustered standard errors at the county level and precision weights. Urban areas exclude sanctuary jurisdictions.

Fig 5 provides estimates from stratified models by census region, rural and urban area, and the proportion likely undocumented. The top panel shows that SC is more strongly related to Hispanic students’ English language arts scores in the South than in other regions. The second panel assesses the impact of SC across sanctuary jurisdictions, urban areas, suburbs, towns, and rural areas. We find that the impact is negative and large in rural areas and towns. In sanctuary jurisdictions, SC is not related to Hispanic students’ English language arts scores. This goes in line with the expectation that the impact of SC is considerably smaller in sanctuary jurisdictions.

The results in the bottom panel show that SC is negatively related to Hispanics students’ English language arts scores in states that fall into the top quartile in terms of the proportion of probabilistic undocumented migrants. In the other quartiles, where the proportion of undocumented is lower, we do not observe an impact of SC on Hispanic students’ English language arts scores. Estimates and standard errors are provided in S7 Table. Results for math are presented in S2 Fig.

About a third of Hispanic students in states with a high proportion of probabilistic undocumented migrants live in the South and the West, respectively. S3 Fig disaggregates census regions into census divisions to gain some insight into how results in the top and bottom panels relate. These estimates suggest that the negative impact observed in states with a high proportion of undocumented migrants is largely due to negative associations observed in the West South Central division (Census region South), which includes Texas, and states that are in the Mountain division (Census region West). The Mountain division includes Arizona, Nevada, and New Mexico. In sum, these results indicate that the impact of SC is especially pronounced in the West South Central and Mountain divisions, towns and rural areas, and in states with a high share of undocumented migrants.

### Sensitivity analyses

I have assessed whether the results hold for alternative ways of estimating the proportion of undocumented migrants and alternate sample restrictions. I assess the proportion of likely undocumented migrants among Hispanics (rather than the total population) and the proportion of likely undocumented migrants in the foreign-born population. These lead to less stable estimates, which is expected given the relatively small Hispanic and foreign-born population in some states. Results based on alternate sample restrictions also show that the findings for Hispanic and white students are robust, while the results for black students are relatively noisy. I have further estimated the models with different sets of control variables (including information on the poverty rate and the proportion of Hispanic students in the school district), which leads to very similar results.

The main analyses estimated the relationship between SC and test scores for Hispanic, white, and black students separately. S4 Fig instead provides estimates of the Hispanic-white and black-white achievement gaps. The top panel shows that the Hispanic-white achievement gap in English language arts increases with the activation of SC in the first two models. However, once we include county or school district fixed effects, the estimate is attenuated. This indicates that unobserved characteristics at the county and school district level are important for changes in the achievement gap. Estimates for the black-white achievement gap in English language arts indicate no change following the implementation of SC. The bottom panel shows that the Hispanic-white and the black-white achievement gaps in math do not change following the implementation of SC, with the exception of the estimate for the Hispanic-white gap which increases in model 2. Again, the estimate is attenuated when we include county and school district fixed effects. In sum, we find no clear association between SC and racial achievement gaps at the school district level, while the main analysis revealed a negative association between the policy implementation and Hispanic students’ achievement. Taken together, these results indicate that SC likely played a notable role for time trends in Hispanic students’ educational outcomes, but that other factors were important for achievement gaps.

## Conclusion and discussion

This study analyzed whether the shock resulting from the implementation of Secure Communities can be linked to changes in Hispanic students’ educational outcomes.

There are three main findings. First, SC has a negative impact on Hispanic students’ school district level achievement in English language arts. In contrast, white and black students’ achievement does not change following the activation of SC. This is in keeping with the view that white and black students are less strongly impacted, as the policy primarily targeted the Hispanic community. The role of SC is substantial in relation to time trends in Hispanic students’ achievement, but modest for racial achievement gaps. When compared to other studies, the negative impact is similar in size for test scores [23], though previous studies find somewhat larger estimates for the achievement gap likely due to different sample restrictions [24]. For Hispanic students’ math scores, no association is observed. This may be due to the fact that Hispanic students’ math skills are less contingent on every-day contact with non-Hispanic neighbors than their English skills, as math can be understood and practiced without firm knowledge of English [20, 53]. Still, the finding that the increase in Hispanic students’ school district level achievement in English language arts is slowed down by the implementation of SC indicates that the shock resulting from SC has a negative, rather than a positive, impact on Hispanic students’ educational outcomes.

Second, stratified results reveal that Hispanic students living in the South, rural areas, and in states with high proportions of likely undocumented migrants are disproportionately impacted by SC. The observed patterns provide some insight into potential mechanisms underlying the associations. Anti-immigrant sentiments, which are especially high in the South and rural areas, may hamper integration [61, 62, 64]. In the rural Midwest, Maya Guatemalan migrants say that they prefer staying indoors as they are scared of being apprehended when driving or walking outside [53]. By reducing interaction with the wider community, immigration enforcement may impact Hispanic students’ English progress. Although the majority of Hispanic students were born in the US and speak some English, 30 percent of US-citizen children with undocumented parents are estimated to have limited English proficiency between ages 5–11, as compared to 18 percent among US citizen-children of migrants [20]. Likewise in the South, undocumented families’ choice of residence is often not only constrained by financial factors but also by assessments of the level of law enforcement patrols [90]. Given that Hispanic students are more likely to be in social proximity to undocumented migrants in areas with high proportions undocumented, findings also corroborate the assumption that Hispanic students are more exposed to SC due to their higher propensity to have undocumented parents, neighbors, or other community members [15].

Third, findings reveal that there is no association between SC and Hispanics students’ test scores in sanctuary jurisdictions. This serves as a placebo test, showing that areas in which the likelihood of deportation was lower were not impacted by SC to the same extent. These cities and counties tend to perceive immigrants as valued members of the community and often provide infrastructure support programs for immigrants aiming to alleviate poverty [64].

The policy implementation analyzed in this paper is specific to the US, but increases in deportations can also be observed in Canada and many European countries [91]. A study from Switzerland indicates that long asylum processes are related to a decrease in the subsequent employment rate of refugees [92]. The authors argue that waiting in limbo fosters psychological discouragement among refugees. In the UK, asylum seekers who receive a negative asylum-application decision also note that this impacts their quality of life and is linked to feelings of distress, isolation, and statelessness [93]. This evidence suggests that policy-related shocks and apprehension status can have negative consequences for migrants’ employment and health outcomes in Europe, which may spill over to their children, as in the US context.

This study has several limitations. The SEDA data are published in aggregated form due to privacy concerns. I use the lowest level of aggregation available, the school district, as the unit of analysis. The SEDA data are also disaggregated by race, and I study the performance of Hispanic, white, and black students separately. Incorporating data from the CPS further allows me to stratify the sample by the proportion of probabilistic undocumented migrants in the area. Still, when inferring individual-level processes from aggregate data, results need to be interpreted with care. Furthermore, since SC was eventually implemented nation-wide, there is no truly unexposed area. Sanctuary jurisdictions provide the closest proxy to unexposed areas.

The study contributes to the literature by illustrating that shocks induced by SC have a negative impact on Hispanic students’ English language arts performance. This is particularly pronounced in the South, rural areas, and areas with high proportions of undocumented migrants. These findings suggest that policy-related shocks can have unintended consequences for educational inequalities in the US, potentially impacting longer term economic trajectories of the children of migrants as well as social inequalities.

In future studies, efforts should be directed at observing the mechanisms at the individual level. Educational achievement may decrease among Hispanic students due to increased stress and anxiety experienced among this group and/or result from reduced interaction with non-Hispanic neighbors and lower investment in the community. It will also be worthwhile to further explore differences in the impact of immigration enforcement by subject.

## Supporting information

S1 Fig Estimated associations between the implementation of Secure Communities and school district level achievement among Hispanic, white, and black students, excluding school districts that are in multiple counties. Data from SEDA 2009–18 and DHS. Coefficients and 95 percent confidence intervals based on Eq 1 are plotted. Models 1–4 show results obtained from standard two-way fixed effect specifications. Model 5 shows results obtained using the method outlined by Sun and Abraham (2021). The y-axis reports the size of the coefficients for Secure Communities and the x-axis reports race. Models are estimated with clustered standard errors at the county level and precision weights.
(TIF)

S2 Fig Estimated associations between the implementation of Secure Communities and school district level math achievement among Hispanic students across census regions, rural-urban areas, and the proportion of likely undocumented migrants. Data from SEDA 2009–18, DHS, and CPS. Coefficients and 95 percent confidence intervals based on Eq 1 are plotted. Results are obtained using the method outlined by Sun and Abraham (2021). The y-axis reports the size of the coefficients for Secure Communities. Models are estimated with clustered standard errors at the county level and precision weights. Urban areas exclude sanctuary jurisdictions.
(TIF)

S3 Fig Estimated associations between the implementation of Secure Communities and school district level English language arts achievement among Hispanic students across census divisions. Data from SEDA 2009–18 and DHS. Coefficients and 95 percent confidence intervals based on Eq 1 are plotted. Results are obtained using the method outlined by Sun and Abraham (2021). The y-axis reports the size of the coefficients for Secure Communities. Models are estimated with clustered standard errors at the county level and precision weights.
(TIF)

S4 Fig Estimated associations between the implementation of Secure Communities and school district level Hispanic-white and black-white achievement gaps. Data from SEDA 2009–18 and DHS. Coefficients and 95 percent confidence intervals based on Eq 1 are plotted. Models 1–4 show results obtained from standard two-way fixed effect specifications. Model 5 shows results obtained using the method outlined by Sun and Abraham (2021). The y-axis reports the size of the coefficients for Secure Communities and the x-axis reports race. Models are estimated with clustered standard errors at the county level and precision weights.
(TIF)

S1 Table Documentation of the assignment of school districts which are in multiple counties. (PDF)

S2 Table Estimated associations between Secure Communities and the proportion of Hispanic students in the school district for grades 3–8. Data from SEDA 2009–18 and DHS. Standard two-way fixed effects specifications are estimated with clustered standard errors at the county level and precision weights. * p < 0.05, ** p < 0.01, *** p < 0.001 (two-tailed).
(PDF)

S3 Table Estimated associations between the implementation of Secure Communities and school district level English language arts achievement among (A) Hispanic, (B) white, and (C) black students. Data from SEDA 2009–18 and DHS. Precision weighted estimates are based on Eq 1. Models 1–4 show results obtained from standard two-way fixed effect specifications. Model 5 shows results obtained using the method outlined by Sun and Abraham (2021). Clustered standard errors at the county level are in parentheses. ^a^ Considering that these are state-level policies, there is no variation once we include state-by-year fixed effects. * p < 0.05, ** p < 0.01, *** p < 0.001 (two-tailed).
(PDF)

S4 Table Estimated associations between the implementation of Secure Communities and school district level math achievement among (A) Hispanic, (B) white, and (C) black students. Data from SEDA 2009–18 and DHS. Precision weighted estimates are based on Eq 1. Models 1–4 show results obtained from standard two-way fixed effect specifications. Model 5 shows results obtained using the method outlined by Sun and Abraham (2021). Clustered standard errors at the county level are in parentheses. ^a^ Considering that these are state-level policies, there is no variation once we include state-by-year fixed effects. * p < 0.05, ** p < 0.01, *** p < 0.001 (two-tailed).
(PDF)

S5 Table Estimated associations between the implementation of Secure Communities and school district level (A) English language arts and (B) math achievement among Hispanic, white, and black students relative to the implementation year. Data from SEDA 2009–18 and DHS. Precision weighted estimates are based on Eq 2. Results are obtained using the method outlined by Sun and Abraham (2021). Clustered standard errors at the county level are in parentheses. * p < 0.05, ** p < 0.01, *** p < 0.001 (two-tailed).
(PDF)

S6 Table Weights for each relative time period obtained in the Sun and Abraham (2021) method. Data from SEDA 2009–18 and DHS. After sample restrictions, there are four cohorts: 2010, 2011, 2012, 2013. The first treated cohort is used as the control group.
(PDF)

S7 Table Estimated associations between Secure Communities and school district level English language arts achievement among Hispanic students across (A) census regions, (B) rural-urban areas, and (C) the proportion of likely undocumented migrants. Data from SEDA 2009–18, DHS, and CPS. Precision weighted estimates are based on Eq 1. Results are obtained using the method outlined by Sun and Abraham (2021). Clustered standard errors at the county level are in parentheses. Urban areas exclude sanctuary jurisdictions. ^a^ No policy identified in the states. * p < 0.05, ** p < 0.01, *** p < 0.001 (two-tailed).
(PDF)
