# Supplementary figures and images for: Apprehension and educational outcomes among Hispanic students in the United States: The impact of Secure Communities
Source: PLoS One. 2022 Oct 24;17(10):e0276636. doi: 10.1371/journal.pone.0276636 (PMC9591052; doi:10.1371/journal.pone.0276636)

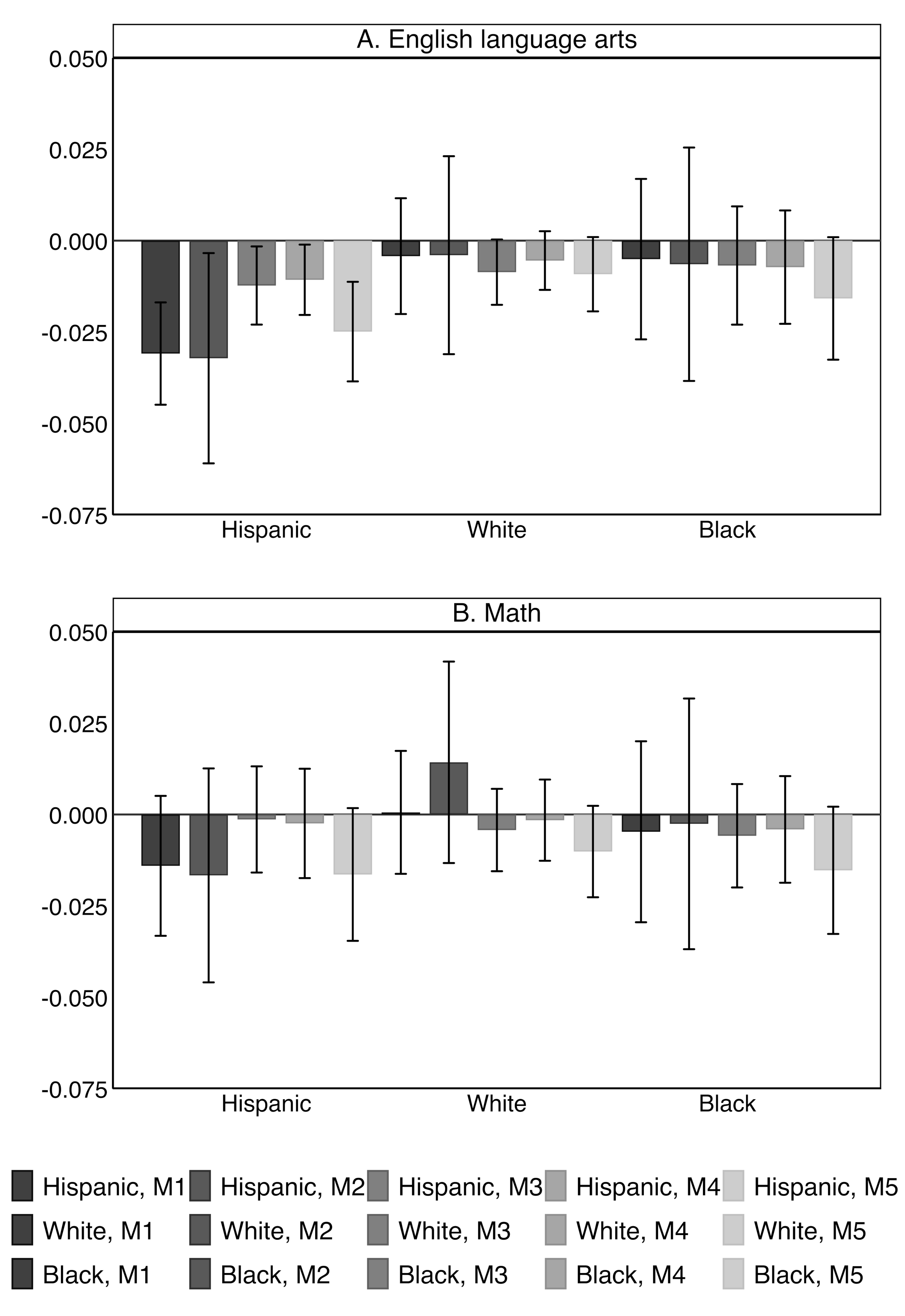

Supplement: S1 Fig — Data from SEDA 2009–18 and DHS. Coefficients and 95 percent confidence intervals based on Eq 1 are plotted. Models 1–4 show results obtained from standard two-way fixed effect specifications. Model 5 shows results obtained using the method outlined by Sun and Abraham (2021). The y-axis reports the size of the coefficients for Secure Communities and the x-axis reports race. Models are estimated with clustered standard errors at the county level and precision weights. (TIF) [file pone.0276636.s001.tif]

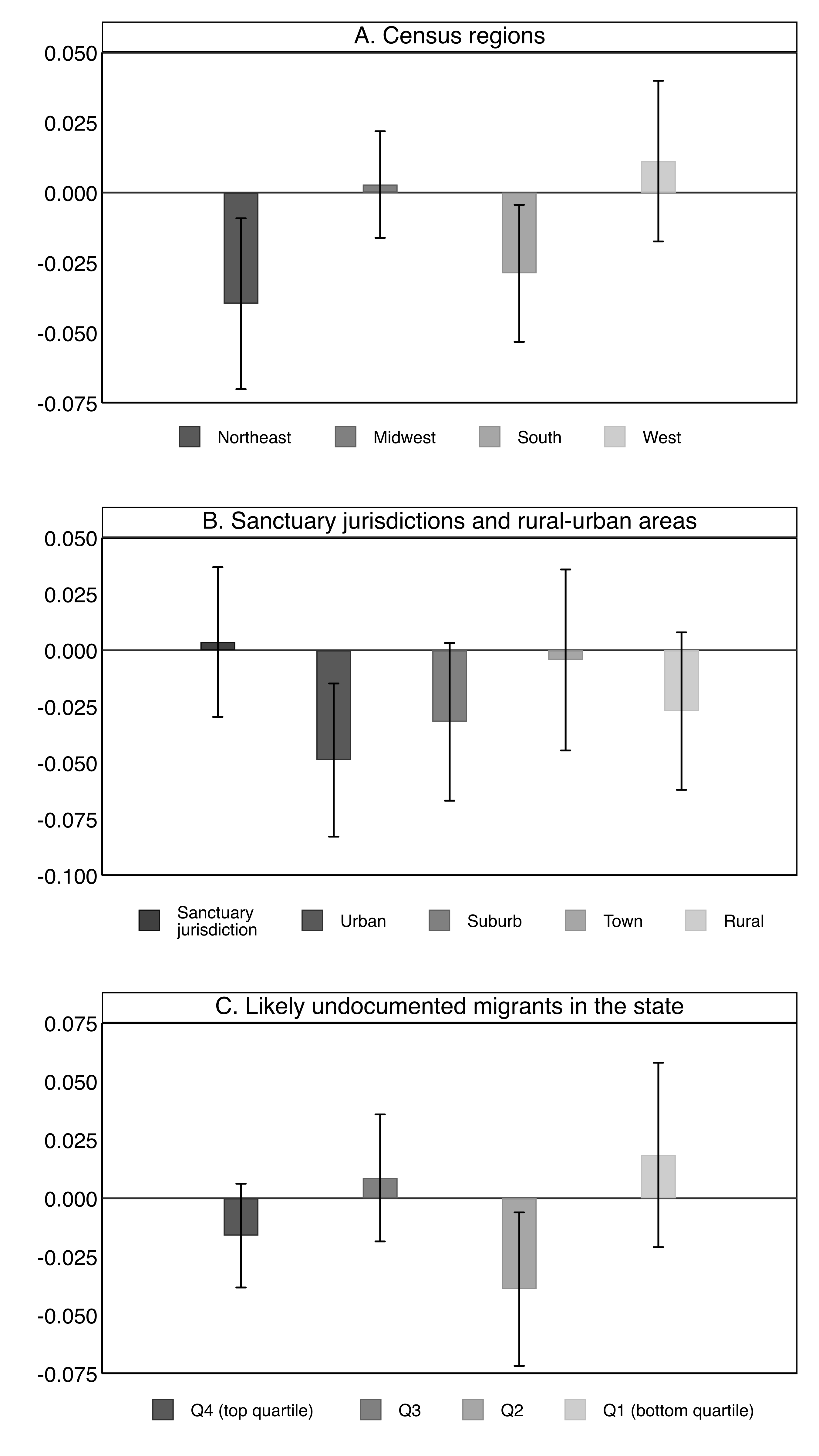

Supplement: S2 Fig — Data from SEDA 2009–18, DHS, and CPS. Coefficients and 95 percent confidence intervals based on Eq 1 are plotted. Results are obtained using the method outlined by Sun and Abraham (2021). The y-axis reports the size of the coefficients for Secure Communities. Models are estimated with clustered standard errors at the county level and precision weights. Urban areas exclude sanctuary jurisdictions. (TIF) [file pone.0276636.s002.tif]

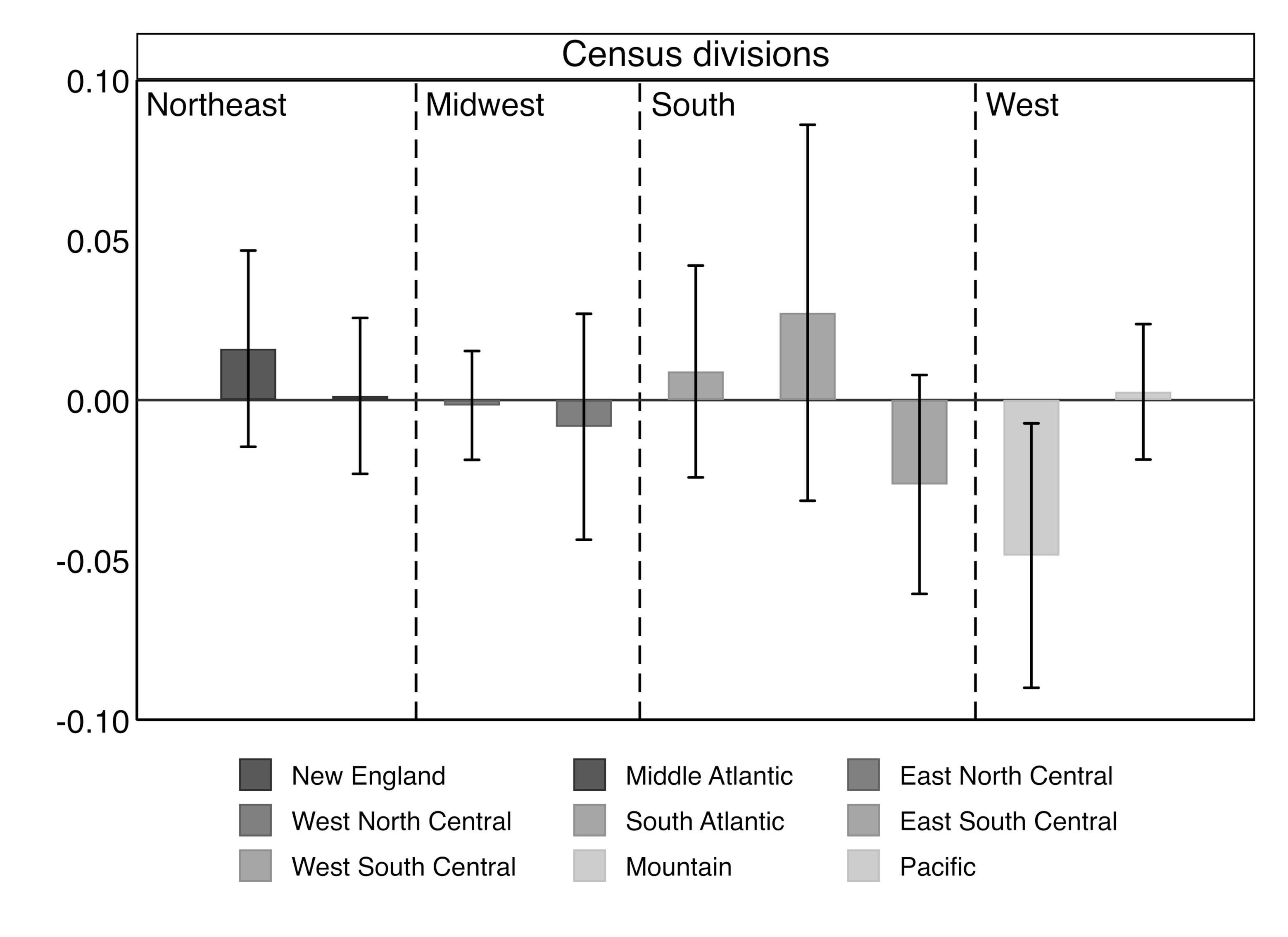

Supplement: S3 Fig — Data from SEDA 2009–18 and DHS. Coefficients and 95 percent confidence intervals based on Eq 1 are plotted. Results are obtained using the method outlined by Sun and Abraham (2021). The y-axis reports the size of the coefficients for Secure Communities. Models are estimated with clustered standard errors at the county level and precision weights. (TIF) [file pone.0276636.s003.tif]

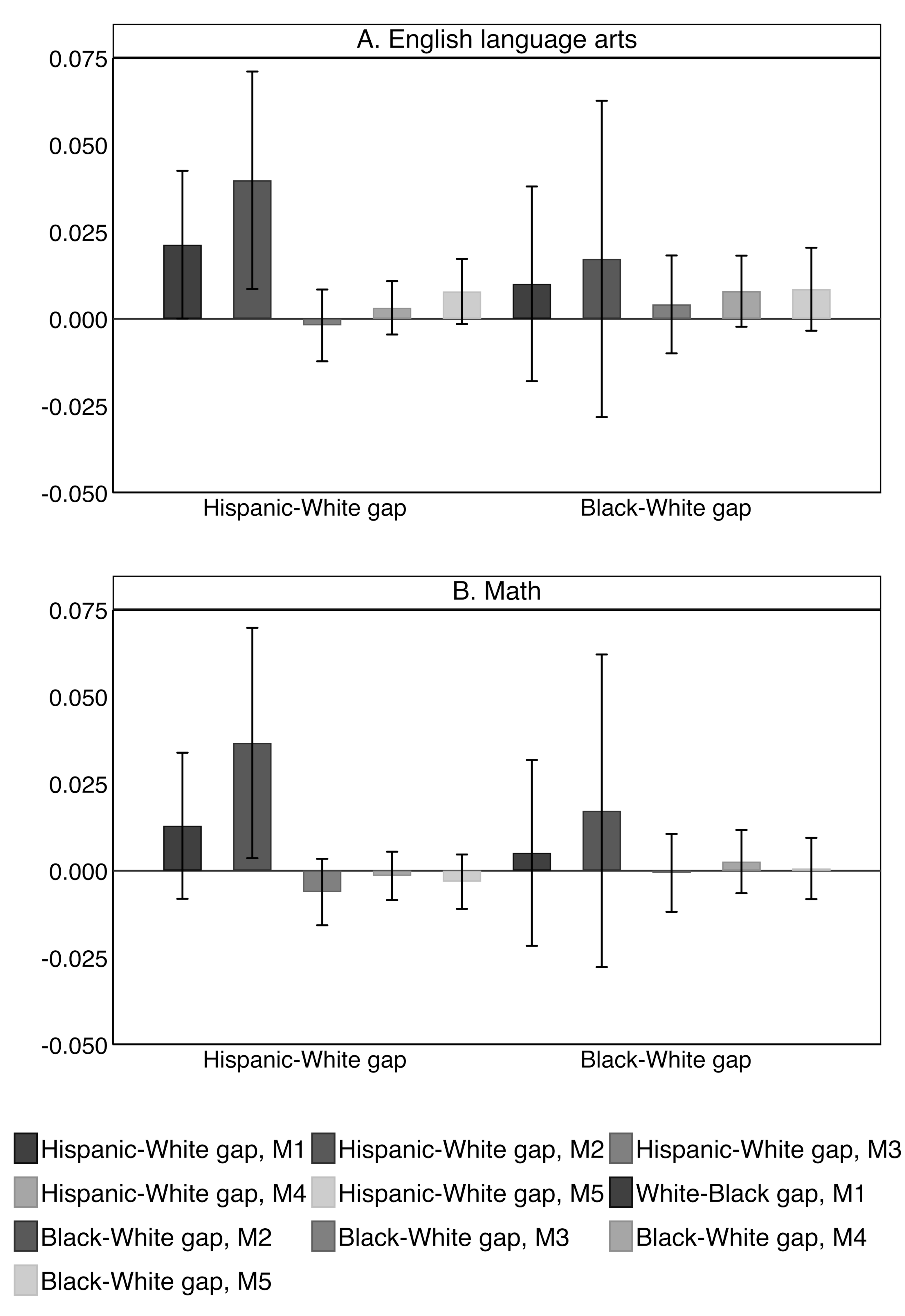

Supplement: S4 Fig — Data from SEDA 2009–18 and DHS. Coefficients and 95 percent confidence intervals based on Eq 1 are plotted. Models 1–4 show results obtained from standard two-way fixed effect specifications. Model 5 shows results obtained using the method outlined by Sun and Abraham (2021). The y-axis reports the size of the coefficients for Secure Communities and the x-axis reports race. Models are estimated with clustered standard errors at the county level and precision weights. (TIF) [file pone.0276636.s004.tif]
