# Supplementary material for: Apprehension and educational outcomes among Hispanic students in the United States: The impact of Secure Communities
Source: PLoS One. 2022 Oct 24;17(10):e0276636. doi: 10.1371/journal.pone.0276636 (PMC9591052; doi:10.1371/journal.pone.0276636)
Supplement: S1 Table — (PDF) [file pone.0276636.s005.pdf]

S1 Table. Documentation of the assignment of school districts which are in multiple counties.

State code	School district ID	County ID	County name	Year SC	County ID New	County name New	Year SC New	Indic-ator
1	100002	1001	Autauga County	2011	1001	Autauga County	2011	0
1	100002	1025	Clarke County	2013	1001	Autauga County	2011	1
1	100002	1073	Jefferson County	2011	1001	Autauga County	2011	0
1	100002	1101	Montgomery County	2013	1001	Autauga County	2011	1
1	100007	1073	Jefferson County	2011	1073	Jefferson County	2011	0
1	100007	1117	Shelby County	2011	1073	Jefferson County	2011	0
1	102700	1081	Lee County	2011	1081	Lee County	2011	0
1	102700	1113	Russell County	2013	1081	Lee County	2011	1
2	200050	2240	Southeast Fairbanks Census Area	2012	2240	Southeast Fairbanks Census Area	2012	0
2	200050	2261	Valdez-Cordova Census Area	2012	2240	Southeast Fairbanks Census Area	2012	0
2	200130	2090	Fairbanks North Star Borough	2012	2090	Fairbanks North Star Borough	2012	0
2	200130	2290	Yukon-Koyukuk Census Area	2012	2090	Fairbanks North Star Borough	2012	0
2	200485	2060	Bristol Bay Borough	2012	2060	Bristol Bay Borough	2012	0
2	200485	2164	Lake And Peninsula Borough	2012	2060	Bristol Bay Borough	2012	0
2	200520	2020	Anchorage Municipality	2012	2020	Anchorage Municipality	2012	0
2	200520	2290	Yukon-Koyukuk Census Area	2012	2020	Anchorage Municipality	2012	0
2	200800	2020	Anchorage Municipality	2012	2020	Anchorage Municipality	2012	0
2	200800	2261	Valdez-Cordova Census Area	2012	2020	Anchorage Municipality	2012	0
4	409160	4007	Gila County	2011	4007	Gila County	2011	0
4	409160	4017	Navajo County	2011	4007	Gila County	2011	0
5	500001	5001	Arkansas County	2012	5001	Arkansas County	2012	0
5	500001	5069	Jefferson County	2011	5001	Arkansas County	2012	1
5	500015	5053	Grant County	2012	5053	Grant County	2012	0
5	500015	5125	Saline County	2011	5053	Grant County	2012	1
5	500068	5027	Columbia County	2012	5027	Columbia County	2012	0
5	500068	5073	Lafayette County	2012	5027	Columbia County	2012	0
5	500073	5097	Montgomery County	2012	5097	Montgomery County	2012	0
5	500073	5113	Polk County	2012	5097	Montgomery County	2012	0
5	500076	5089	Marion County	2012	5089	Marion County	2012	0
5	500076	5101	Newton County	2012	5089	Marion County	2012	0
5	500076	5129	Searcy County	2012	5089	Marion County	2012	0
5	500405	5061	Howard County	2012	5061	Howard County	2012	0
5	500405	5113	Polk County	2012	5061	Howard County	2012	0
5	503300	5057	Hempstead County	2012	5057	Hempstead County	2012	0
5	503300	5099	Nevada County	2012	5057	Hempstead County	2012	0
5	503710	5031	Craighead County	2012	5031	Craighead County	2012	0
5	503710	5093	Mississippi County	2012	5031	Craighead County	2012	0
5	504740	5047	Franklin County	2012	5047	Franklin County	2012	0
5	504740	5083	Logan County	2012	5047	Franklin County	2012	0
5	506690	5019	Clark County	2012	5019	Clark County	2012	0
5	506690	5109	Pike County	2012	5019	Clark County	2012	0
5	507290	5039	Dallas County	2012	5039	Dallas County	2012	0
5	507290	5103	Ouachita County	2012	5039	Dallas County	2012	0
5	507860	5081	Little River County	2012	5081	Little River County	2012	0
5	507860	5133	Sevier County	2012	5081	Little River County	2012	0
5	508040	5035	Crittenden County	2012	5035	Crittenden County	2012	0
5	508040	5123	St. Francis County	2012	5035	Crittenden County	2012	0
5	508240	5071	Johnson County	2012	5071	Johnson County	2012	0
5	508240	5087	Madison County	2012	5071	Johnson County	2012	0
5	508240	5101	Newton County	2012	5071	Johnson County	2012	0
5	509780	5057	Hempstead County	2012	5057	Hempstead County	2012	0
5	509780	5061	Howard County	2012	5057	Hempstead County	2012	0
5	510290	5033	Crawford County	2011	5033	Crawford County	2011	0
5	510290	5047	Franklin County	2012	5033	Crawford County	2011	1
5	512480	5075	Lawrence County	2012	5075	Lawrence County	2012	0
5	512480	5121	Randolph County	2012	5075	Lawrence County	2012	0
5	512660	5007	Benton County	2011	5007	Benton County	2011	0
5	512660	5143	Washington County	2011	5007	Benton County	2011	0

6	600033	6019	Fresno County	2010	6019	Fresno County	2010	0
6	600033	6047	Merced County	2011	6019	Fresno County	2010	1
6	604080	6039	Madera County	2011	6039	Madera County	2011	0
6	604080	6043	Mariposa County	2011	6039	Madera County	2011	0
6	605010	6035	Lassen County	2011	6035	Lassen County	2011	0
6	605010	6049	Modoc County	2011	6035	Lassen County	2011	0
6	607900	6061	Placer County	2011	6061	Placer County	2011	0
6	607900	6067	Sacramento County	2010	6061	Placer County	2011	1
6	609070	6025	Imperial County	2010	6025	Imperial County	2010	0
6	609070	6065	Riverside County	2010	6025	Imperial County	2010	0
6	611490	6061	Placer County	2011	6061	Placer County	2011	0
6	611490	6067	Sacramento County	2010	6061	Placer County	2011	1
6	623010	6037	Los Angeles County	2010	6037	Los Angeles County	2010	0
6	623010	6059	Orange County	2010	6037	Los Angeles County	2010	0
6	629490	6053	Monterey County	2010	6053	Monterey County	2010	0
6	629490	6087	Santa Cruz County	2011	6053	Monterey County	2010	1
6	633110	6067	Sacramento County	2010	6067	Sacramento County	2010	0
6	633110	6095	Solano County	2010	6067	Sacramento County	2010	0
6	633110	6113	Yolo County	2011	6067	Sacramento County	2010	1
6	636450	6053	Monterey County	2010	6053	Monterey County	2010	0
6	636450	6079	San Luis Obispo County	2010	6053	Monterey County	2010	0
6	636670	6041	Marin County	2011	6041	Marin County	2011	0
6	636670	6097	Sonoma County	2010	6041	Marin County	2011	1
6	638070	6011	Colusa County	2011	6011	Colusa County	2011	0
6	638070	6021	Glenn County	2011	6011	Colusa County	2011	0
6	638770	6057	Nevada County	2011	6057	Nevada County	2011	0
6	638770	6061	Placer County	2011	6057	Nevada County	2011	0
6	643560	6065	Riverside County	2010	6065	Riverside County	2010	0
6	643560	6071	San Bernardino County	2010	6065	Riverside County	2010	0
6	691093	6049	Modoc County	2011	6049	Modoc County	2011	0
6	691093	6093	Siskiyou County	2011	6049	Modoc County	2011	0
6	691136	6053	Monterey County	2010	6053	Monterey County	2010	0
6	691136	6069	San Benito County	2011	6053	Monterey County	2010	1
8	800282	8001	Adams County	2012	8001	Adams County	2012	0
8	800282	8005	Arapahoe County	2011	8001	Adams County	2012	1
8	802340	8001	Adams County	2012	8001	Adams County	2012	0
8	802340	8005	Arapahoe County	2011	8001	Adams County	2012	1
8	802490	8013	Boulder County	2012	8013	Boulder County	2012	0
8	802490	8014	Broomfield County	2012	8013	Boulder County	2012	0
8	804260	8037	Eagle County	2012	8037	Eagle County	2012	0
8	804260	8045	Garfield County	2012	8037	Eagle County	2012	0
8	804800	8014	Broomfield County	2012	8014	Broomfield County	2012	0
8	804800	8059	Jefferson County	2012	8014	Broomfield County	2012	0
8	805370	8013	Boulder County	2012	8013	Boulder County	2012	0
8	805370	8123	Weld County	2012	8013	Boulder County	2012	0
8	806900	8001	Adams County	2012	8001	Adams County	2012	0
8	806900	8014	Broomfield County	2012	8001	Adams County	2012	0
9	903520	9003	Hartford County	2012	9003	Hartford County	2012	0
9	903520	9005	Litchfield County	2012	9003	Hartford County	2012	0
10	1001080	10001	Kent County	2010	10001	Kent County	2010	0
10	1001080	10005	Sussex County	2010	10001	Kent County	2010	0
11	1100030	11001	District Of Columbia	2012	11001	District Of Columbia	2012	0
12	1201560	12017	Citrus County	2011	12017	Citrus County	2011	0
12	1201560	12053	Hernando County	2011	12017	Citrus County	2011	0
12	1201560	12103	Pinellas County	2010	12017	Citrus County	2011	1
12	1202010	12093	Okeechobee County	2011	12093	Okeechobee County	2011	0
12	1202010	12133	Washington County	2011	12093	Okeechobee County	2011	0
13	1300120	13089	Dekalb County	2010	13089	Dekalb County	2010	0
13	1300120	13121	Fulton County	2011	13089	Dekalb County	2010	1
13	1302580	13011	Banks County	2012	13011	Banks County	2012	0
13	1302580	13137	Habersham County	2012	13011	Banks County	2012	0
15	1500030	15001	Hawaii County	2011	15001	Hawaii County	2011	0
15	1500030	15007	Kauai County	2012	15001	Hawaii County	2011	1

15	1500030	15009	Maui County	2012	15001	Hawaii County	2011	1
16	1600720	16037	Custer County	2012	16037	Custer County	2012	0
16	1600720	16059	Lemhi County	2012	16037	Custer County	2012	0
16	1601290	16029	Caribou County	2012	16029	Caribou County	2012	0
16	1601290	16041	Franklin County	2012	16029	Caribou County	2012	0
16	1601650	16055	Kootenai County	2011	16055	Kootenai County	2011	0
16	1601650	16079	Shoshone County	2012	16055	Kootenai County	2011	1
16	1602520	16035	Clearwater County	2012	16035	Clearwater County	2012	0
16	1602520	16069	Nez Perce County	2012	16035	Clearwater County	2012	0
17	1700003	17039	De Witt County	2011	17039	De Witt County	2011	0
17	1700003	17147	Piatt County	2011	17039	De Witt County	2011	0
17	1700005	17105	Livingston County	2013	17105	Livingston County	2013	0
17	1700005	17113	Mclean County	2013	17105	Livingston County	2013	0
17	1700122	17041	Douglas County	2013	17041	Douglas County	2013	0
17	1700122	17045	Edgar County	2013	17041	Douglas County	2013	0
17	1700162	17001	Adams County	2013	17001	Adams County	2013	0
17	1700162	17149	Pike County	2013	17001	Adams County	2013	0
17	1700165	17049	Effingham County	2011	17049	Effingham County	2011	0
17	1700165	17051	Fayette County	2013	17049	Effingham County	2011	1
17	1700165	17135	Montgomery County	2013	17049	Effingham County	2011	1
17	1700166	17007	Boone County	2013	17007	Boone County	2013	0
17	1700166	17201	Winnebago County	2010	17007	Boone County	2013	1
17	1700169	17061	Greene County	2013	17061	Greene County	2013	0
17	1700169	17117	Macoupin County	2013	17061	Greene County	2013	0
17	1700170	17085	Jo Daviess County	2013	17085	Jo Daviess County	2013	0
17	1700170	17177	Stephenson County	2011	17085	Jo Daviess County	2013	1
17	1700174	17081	Jefferson County	2013	17081	Jefferson County	2013	0
17	1700174	17121	Marion County	2011	17081	Jefferson County	2013	1
17	1700175	17029	Coles County	2013	17029	Coles County	2013	0
17	1700175	17045	Edgar County	2013	17029	Coles County	2013	0
17	1700175	17173	Shelby County	2013	17029	Coles County	2013	0
17	1700184	17063	Grundy County	2013	17063	Grundy County	2013	0
17	1700184	17093	Kendall County	2013	17063	Grundy County	2013	0
17	1700186	17057	Fulton County	2013	17057	Fulton County	2013	0
17	1700186	17067	Hancock County	2013	17057	Fulton County	2013	0
17	1700186	17109	Mcdonough County	2013	17057	Fulton County	2013	0
17	1700190	17077	Jackson County	2011	17077	Jackson County	2011	0
17	1700190	17145	Perry County	2013	17077	Jackson County	2011	1
17	1700191	17089	Kane County	2010	17089	Kane County	2010	0
17	1700191	17111	Mchenry County	2010	17089	Kane County	2010	0
17	1700192	17095	Knox County	2013	17095	Knox County	2013	0
17	1700192	17187	Warren County	2013	17095	Knox County	2013	0
17	1700195	17103	Lee County	2013	17103	Lee County	2013	0
17	1700195	17141	Ogle County	2013	17103	Lee County	2013	0
17	1700195	17195	Whiteside County	2013	17103	Lee County	2013	0
17	1700206	17129	Menard County	2013	17129	Menard County	2013	0
17	1700206	17167	Sangamon County	2013	17129	Menard County	2013	0
17	1700223	17139	Moultrie County	2013	17139	Moultrie County	2013	0
17	1700223	17173	Shelby County	2013	17139	Moultrie County	2013	0
17	1700324	17115	Macon County	2013	17115	Macon County	2013	0
17	1700324	17167	Sangamon County	2013	17115	Macon County	2013	0
17	1700326	17113	Mclean County	2013	17113	Mclean County	2013	0
17	1700326	17203	Woodford County	2013	17113	Mclean County	2013	0
17	1700332	17109	Mcdonough County	2013	17109	Mcdonough County	2013	0
17	1700332	17169	Schuyler County	2013	17109	Mcdonough County	2013	0
17	1701411	17103	Lee County	2013	17103	Lee County	2013	0
17	1701411	17195	Whiteside County	2013	17103	Lee County	2013	0
17	1701413	17057	Fulton County	2013	17057	Fulton County	2013	0
17	1701413	17095	Knox County	2013	17057	Fulton County	2013	0
17	1704260	17041	Douglas County	2013	17041	Douglas County	2013	0
17	1704260	17139	Moultrie County	2013	17041	Douglas County	2013	0
17	1704260	17147	Piatt County	2011	17041	Douglas County	2013	1
17	1704470	17129	Menard County	2013	17129	Menard County	2013	0

17	1704470	17167	Sangamon County	2013	17129	Menard County	2013	0
17	1705050	17031	Cook County	2013	17031	Cook County	2013	0
17	1705050	17089	Kane County	2010	17031	Cook County	2013	1
17	1705050	17097	Lake County	2010	17031	Cook County	2013	1
17	1708550	17089	Kane County	2010	17089	Kane County	2010	0
17	1708550	17111	Mchenry County	2010	17089	Kane County	2010	0
17	1709100	17021	Christian County	2011	17021	Christian County	2011	0
17	1709100	17173	Shelby County	2013	17021	Christian County	2011	1
17	1709270	17027	Clinton County	2011	17027	Clinton County	2011	0
17	1709270	17121	Marion County	2011	17027	Clinton County	2011	0
17	1710650	17119	Madison County	2010	17119	Madison County	2010	0
17	1710650	17163	St. Clair County	2010	17119	Madison County	2010	0
17	1712270	17011	Bureau County	2013	17011	Bureau County	2013	0
17	1712270	17099	Lasalle County	2013	17011	Bureau County	2013	0
17	1713710	17031	Cook County	2013	17031	Cook County	2013	0
17	1713710	17043	Dupage County	2010	17031	Cook County	2013	1
17	1713710	17089	Kane County	2010	17031	Cook County	2013	1
17	1715100	17123	Marshall County	2013	17123	Marshall County	2013	0
17	1715100	17203	Woodford County	2013	17123	Marshall County	2013	0
17	1715490	17141	Ogle County	2013	17141	Ogle County	2013	0
17	1715490	17177	Stephenson County	2011	17141	Ogle County	2013	1
17	1719260	17037	Dekalb County	2013	17037	Dekalb County	2013	0
17	1719260	17089	Kane County	2010	17037	Dekalb County	2013	1
17	1721000	17011	Bureau County	2013	17011	Bureau County	2013	0
17	1721000	17073	Henry County	2013	17011	Bureau County	2013	0
17	1726310	17063	Grundy County	2013	17063	Grundy County	2013	0
17	1726310	17093	Kendall County	2013	17063	Grundy County	2013	0
17	1726310	17197	Will County	2010	17063	Grundy County	2013	1
17	1727710	17043	Dupage County	2010	17043	Dupage County	2010	0
17	1727710	17197	Will County	2010	17043	Dupage County	2010	0
17	1729100	17097	Lake County	2010	17097	Lake County	2010	0
17	1729100	17143	Peoria County	2013	17097	Lake County	2010	1
17	1729890	17107	Logan County	2013	17107	Logan County	2013	0
17	1729890	17113	Mclean County	2013	17107	Logan County	2013	0
17	1729890	17179	Tazewell County	2013	17107	Logan County	2013	0
17	1730270	17093	Kendall County	2013	17093	Kendall County	2013	0
17	1730270	17197	Will County	2010	17093	Kendall County	2013	1
17	1730630	17021	Christian County	2011	17021	Christian County	2011	0
17	1730630	17173	Shelby County	2013	17021	Christian County	2011	1
17	1731740	17093	Kendall County	2013	17093	Kendall County	2013	0
17	1731740	17197	Will County	2010	17093	Kendall County	2013	1
17	1736180	17131	Mercer County	2013	17131	Mercer County	2013	0
17	1736180	17161	Rock Island County	2013	17131	Mercer County	2013	0
17	1737170	17043	Dupage County	2010	17043	Dupage County	2010	0
17	1737170	17089	Kane County	2010	17043	Dupage County	2010	0
17	1737590	17117	Macoupin County	2013	17117	Macoupin County	2013	0
17	1737590	17119	Madison County	2010	17117	Macoupin County	2013	1
17	1737680	17031	Cook County	2013	17031	Cook County	2013	0
17	1737680	17197	Will County	2010	17031	Cook County	2013	1
17	1740920	17123	Marshall County	2013	17123	Marshall County	2013	0
17	1740920	17203	Woodford County	2013	17123	Marshall County	2013	0
17	1741190	17097	Lake County	2010	17097	Lake County	2010	0
17	1741190	17111	Mchenry County	2010	17097	Lake County	2010	0
17	1741690	17043	Dupage County	2010	17043	Dupage County	2010	0
17	1741690	17197	Will County	2010	17043	Dupage County	2010	0
17	1742990	17077	Jackson County	2011	17077	Jackson County	2011	0
17	1742990	17181	Union County	2013	17077	Jackson County	2011	1
17	1743962	17125	Mason County	2013	17125	Mason County	2013	0
17	1743962	17179	Tazewell County	2013	17125	Mason County	2013	0
18	1800390	18047	Franklin County	2012	18047	Franklin County	2012	0
18	1800390	18137	Ripley County	2012	18047	Franklin County	2012	0
18	1801060	18065	Henry County	2011	18065	Henry County	2011	0
18	1801060	18139	Rush County	2012	18065	Henry County	2011	1

18	1802520	18099	Marshall County	2012	18099	Marshall County	2012	0
18	1802520	18131	Pulaski County	2011	18099	Marshall County	2012	1
18	1806240	18053	Grant County	2011	18053	Grant County	2011	0
18	1806240	18095	Madison County	2011	18053	Grant County	2011	0
18	1806570	18091	Laporte County	2012	18091	Laporte County	2012	0
18	1806570	18127	Porter County	2012	18091	Laporte County	2012	0
18	1807560	18091	Laporte County	2012	18091	Laporte County	2012	0
18	1807560	18141	St. Joseph County	2012	18091	Laporte County	2012	0
18	1811190	18029	Dearborn County	2012	18029	Dearborn County	2012	0
18	1811190	18137	Ripley County	2012	18029	Dearborn County	2012	0
18	1811370	18049	Fulton County	2011	18049	Fulton County	2011	0
18	1811370	18085	Kosciusko County	2012	18049	Fulton County	2011	1
18	1811910	18099	Marshall County	2012	18099	Marshall County	2012	0
18	1811910	18141	St. Joseph County	2012	18099	Marshall County	2012	0
18	1813230	18085	Kosciusko County	2012	18085	Kosciusko County	2012	0
18	1813230	18183	Whitley County	2012	18085	Kosciusko County	2012	0
19	1900022	19013	Black Hawk County	2011	19013	Black Hawk County	2011	0
19	1900022	19171	Tama County	2012	19013	Black Hawk County	2011	1
19	1900028	19025	Calhoun County	2012	19025	Calhoun County	2012	0
19	1900028	19187	Webster County	2012	19025	Calhoun County	2012	0
19	1900060	19127	Marshall County	2012	19127	Marshall County	2012	0
19	1900060	19171	Tama County	2012	19127	Marshall County	2012	0
19	1903060	19075	Grundy County	2012	19075	Grundy County	2012	0
19	1903060	19083	Hardin County	2011	19075	Grundy County	2012	1
19	1903780	19027	Carroll County	2012	19027	Carroll County	2012	0
19	1903780	19047	Crawford County	2012	19027	Carroll County	2012	0
19	1903850	19063	Emmet County	2012	19063	Emmet County	2012	0
19	1903850	19109	Kossuth County	2012	19063	Emmet County	2012	0
19	1904440	19075	Grundy County	2012	19075	Grundy County	2012	0
19	1904440	19083	Hardin County	2011	19075	Grundy County	2012	1
19	1904950	19123	Mahaska County	2012	19123	Mahaska County	2012	0
19	1904950	19179	Wapello County	2012	19123	Mahaska County	2012	0
19	1906660	19011	Benton County	2011	19011	Benton County	2011	0
19	1906660	19113	Linn County	2011	19011	Benton County	2011	0
19	1907590	19095	Iowa County	2012	19095	Iowa County	2012	0
19	1907590	19103	Johnson County	2011	19095	Iowa County	2012	1
19	1908310	19003	Adams County	2012	19003	Adams County	2012	0
19	1908310	19175	Union County	2012	19003	Adams County	2012	0
19	1908520	19049	Dallas County	2012	19049	Dallas County	2012	0
19	1908520	19153	Polk County	2011	19049	Dallas County	2012	1
19	1909120	19023	Butler County	2012	19023	Butler County	2012	0
19	1909120	19075	Grundy County	2012	19023	Butler County	2012	0
19	1909570	19047	Crawford County	2012	19047	Crawford County	2012	0
19	1909570	19085	Harrison County	2012	19047	Crawford County	2012	0
19	1910340	19035	Cherokee County	2012	19035	Cherokee County	2012	0
19	1910340	19193	Woodbury County	2011	19035	Cherokee County	2012	1
19	1910500	19043	Clayton County	2012	19043	Clayton County	2012	0
19	1910500	19055	Delaware County	2012	19043	Clayton County	2012	0
19	1911250	19009	Audubon County	2012	19009	Audubon County	2012	0
19	1911250	19165	Shelby County	2012	19009	Audubon County	2012	0
19	1911790	19081	Hancock County	2012	19081	Hancock County	2012	0
19	1911790	19189	Winnebago County	2012	19081	Hancock County	2012	0
19	1912330	19033	Cerro Gordo County	2012	19033	Cerro Gordo County	2012	0
19	1912330	19081	Hancock County	2012	19033	Cerro Gordo County	2012	0
19	1912660	19075	Grundy County	2012	19075	Grundy County	2012	0
19	1912660	19171	Tama County	2012	19075	Grundy County	2012	0
19	1912810	19059	Dickinson County	2012	19059	Dickinson County	2012	0
19	1912810	19147	Palo Alto County	2012	19059	Dickinson County	2012	0
19	1913230	19029	Cass County	2012	19029	Cass County	2012	0
19	1913230	19137	Montgomery County	2012	19029	Cass County	2012	0
19	1913470	19023	Butler County	2012	19023	Butler County	2012	0
19	1913470	19069	Franklin County	2012	19023	Butler County	2012	0
19	1914280	19089	Howard County	2012	19089	Howard County	2012	0

19	1914280	19191	Winneshiek County	2012	19089	Howard County	2012	0
19	1914880	19027	Carroll County	2012	19027	Carroll County	2012	0
19	1914880	19165	Shelby County	2012	19027	Carroll County	2012	0
19	1915750	19149	Plymouth County	2012	19149	Plymouth County	2012	0
19	1915750	19193	Woodbury County	2011	19149	Plymouth County	2012	1
19	1917880	19081	Hancock County	2012	19081	Hancock County	2012	0
19	1917880	19109	Kossuth County	2012	19081	Hancock County	2012	0
19	1918330	19025	Calhoun County	2012	19025	Calhoun County	2012	0
19	1918330	19187	Webster County	2012	19025	Calhoun County	2012	0
19	1918480	19133	Monona County	2012	19133	Monona County	2012	0
19	1918480	19193	Woodbury County	2011	19133	Monona County	2012	1
19	1919140	19103	Johnson County	2011	19103	Johnson County	2011	0
19	1919140	19183	Washington County	2012	19103	Johnson County	2011	1
19	1919200	19045	Clinton County	2011	19045	Clinton County	2011	0
19	1919200	19105	Jones County	2012	19045	Clinton County	2011	1
19	1919740	19053	Decatur County	2012	19053	Decatur County	2012	0
19	1919740	19185	Wayne County	2012	19053	Decatur County	2012	0
19	1919860	19159	Ringgold County	2012	19159	Ringgold County	2012	0
19	1919860	19173	Taylor County	2012	19159	Ringgold County	2012	0
19	1920190	19017	Bremer County	2012	19017	Bremer County	2012	0
19	1920190	19037	Chickasaw County	2012	19017	Bremer County	2012	0
19	1920760	19067	Floyd County	2012	19067	Floyd County	2012	0
19	1920760	19195	Worth County	2012	19067	Floyd County	2012	0
19	1921600	19093	Ida County	2012	19093	Ida County	2012	0
19	1921600	19161	Sac County	2012	19093	Ida County	2012	0
19	1923190	19025	Calhoun County	2012	19025	Calhoun County	2012	0
19	1923190	19151	Pocahontas County	2012	19025	Calhoun County	2012	0
19	1925920	19033	Cerro Gordo County	2012	19033	Cerro Gordo County	2012	0
19	1925920	19069	Franklin County	2012	19033	Cerro Gordo County	2012	0
19	1926070	19071	Fremont County	2012	19071	Fremont County	2012	0
19	1926070	19145	Page County	2012	19071	Fremont County	2012	0
19	1927500	19049	Dallas County	2012	19049	Dallas County	2012	0
19	1927500	19077	Guthrie County	2012	19049	Dallas County	2012	0
19	1927600	19017	Bremer County	2012	19017	Bremer County	2012	0
19	1927600	19037	Chickasaw County	2012	19017	Bremer County	2012	0
19	1928680	19049	Dallas County	2012	19049	Dallas County	2012	0
19	1928680	19153	Polk County	2011	19049	Dallas County	2012	1
19	1929490	19087	Henry County	2012	19087	Henry County	2012	0
19	1929490	19183	Washington County	2012	19087	Henry County	2012	0
19	1929760	19017	Bremer County	2012	19017	Bremer County	2012	0
19	1929760	19019	Buchanan County	2012	19017	Bremer County	2012	0
19	1930540	19017	Bremer County	2012	19017	Bremer County	2012	0
19	1930540	19023	Butler County	2012	19017	Bremer County	2012	0
19	1931350	19055	Delaware County	2012	19055	Delaware County	2012	0
19	1931350	19061	Dubuque County	2011	19055	Delaware County	2012	1
19	1932010	19015	Boone County	2012	19015	Boone County	2012	0
19	1932010	19049	Dallas County	2012	19015	Boone County	2012	0
19	1999019	19015	Boone County	2012	19015	Boone County	2012	0
19	1999019	19187	Webster County	2012	19015	Boone County	2012	0
20	2000346	20147	Phillips County	2012	20147	Phillips County	2012	0
20	2000346	20183	Smith County	2012	20147	Phillips County	2012	0
20	2000349	20009	Barton County	2012	20009	Barton County	2012	0
20	2000349	20053	Ellsworth County	2012	20009	Barton County	2012	0
20	2000349	20159	Rice County	2012	20009	Barton County	2012	0
20	2000350	20117	Marshall County	2012	20117	Marshall County	2012	0
20	2000350	20131	Nemaha County	2012	20117	Marshall County	2012	0
20	2003270	20137	Norton County	2012	20137	Norton County	2012	0
20	2003270	20147	Phillips County	2012	20137	Norton County	2012	0
20	2003360	20015	Butler County	2012	20015	Butler County	2012	0
20	2003360	20173	Sedgwick County	2011	20015	Butler County	2012	1
20	2003870	20089	Jewell County	2012	20089	Jewell County	2012	0
20	2003870	20123	Mitchell County	2012	20089	Jewell County	2012	0
20	2004470	20123	Mitchell County	2012	20123	Mitchell County	2012	0

20	2004470	20141	Osborne County	2012	20123	Mitchell County	2012	0
20	2004560	20117	Marshall County	2012	20117	Marshall County	2012	0
20	2004560	20131	Nemaha County	2012	20117	Marshall County	2012	0
20	2004710	20021	Cherokee County	2012	20021	Cherokee County	2012	0
20	2004710	20037	Crawford County	2012	20021	Cherokee County	2012	0
20	2004740	20125	Montgomery County	2012	20125	Montgomery County	2012	0
20	2004740	20133	Neosho County	2012	20125	Montgomery County	2012	0
20	2004770	20099	Labette County	2012	20099	Labette County	2012	0
20	2004770	20133	Neosho County	2012	20099	Labette County	2012	0
20	2004950	20029	Cloud County	2012	20029	Cloud County	2012	0
20	2004950	20201	Washington County	2012	20029	Cloud County	2012	0
20	2005280	20127	Morris County	2012	20127	Morris County	2012	0
20	2005280	20197	Wabaunsee County	2012	20127	Morris County	2012	0
20	2006840	20079	Harvey County	2012	20079	Harvey County	2012	0
20	2006840	20173	Sedgwick County	2011	20079	Harvey County	2012	1
20	2006960	20155	Reno County	2012	20155	Reno County	2012	0
20	2006960	20173	Sedgwick County	2011	20155	Reno County	2012	1
20	2007440	20041	Dickinson County	2012	20041	Dickinson County	2012	0
20	2007440	20127	Morris County	2012	20041	Dickinson County	2012	0
20	2007500	20049	Elk County	2012	20049	Elk County	2012	0
20	2007500	20073	Greenwood County	2012	20049	Elk County	2012	0
20	2007890	20061	Geary County	2012	20061	Geary County	2012	0
20	2007890	20161	Riley County	2012	20061	Geary County	2012	0
20	2007970	20085	Jackson County	2012	20085	Jackson County	2012	0
20	2007970	20149	Pottawatomie County	2012	20085	Jackson County	2012	0
20	2007970	20177	Shawnee County	2011	20085	Jackson County	2012	1
20	2008190	20119	Meade County	2012	20119	Meade County	2012	0
20	2008190	20175	Seward County	2012	20119	Meade County	2012	0
20	2008250	20107	Linn County	2012	20107	Linn County	2012	0
20	2008250	20121	Miami County	2011	20107	Linn County	2012	1
20	2008880	20113	Mcpherson County	2012	20113	Mcpherson County	2012	0
20	2008880	20159	Rice County	2012	20113	Mcpherson County	2012	0
20	2010620	20045	Douglas County	2011	20045	Douglas County	2011	0
20	2010620	20087	Jefferson County	2012	20045	Douglas County	2011	1
20	2010980	20149	Pottawatomie County	2012	20149	Pottawatomie County	2012	0
20	2010980	20161	Riley County	2012	20149	Pottawatomie County	2012	0
20	2011040	20179	Sheridan County	2012	20179	Sheridan County	2012	0
20	2011040	20193	Thomas County	2012	20179	Sheridan County	2012	0
20	2011850	20091	Johnson County	2011	20091	Johnson County	2011	0
20	2011850	20121	Miami County	2011	20091	Johnson County	2011	0
20	2012120	20105	Lincoln County	2012	20105	Lincoln County	2012	0
20	2012120	20167	Russell County	2012	20105	Lincoln County	2012	0
20	2012300	20015	Butler County	2012	20015	Butler County	2012	0
20	2012300	20173	Sedgwick County	2011	20015	Butler County	2012	1
21	2101110	21009	Barren County	2012	21009	Barren County	2012	0
21	2101110	21099	Hart County	2012	21009	Barren County	2012	0
21	2101320	21121	Knox County	2012	21121	Knox County	2012	0
21	2101320	21235	Whitley County	2012	21121	Knox County	2012	0
21	2101860	21067	Fayette County	2011	21067	Fayette County	2011	0
21	2101860	21209	Scott County	2012	21067	Fayette County	2011	1
21	2104980	21019	Boyd County	2012	21019	Boyd County	2012	0
21	2104980	21089	Greenup County	2012	21019	Boyd County	2012	0
22	2200057	22001	Acadia Parish	2012	22001	Acadia Parish	2012	0
22	2200057	22005	Ascension Parish	2012	22001	Acadia Parish	2012	0
22	2200057	22015	Bossier Parish	2012	22001	Acadia Parish	2012	0
22	2200057	22019	Calcasieu Parish	2012	22001	Acadia Parish	2012	0
22	2200057	22033	East Baton Rouge Parish	2011	22001	Acadia Parish	2012	1
22	2200057	22051	Jefferson Parish	2010	22001	Acadia Parish	2012	1
22	2200057	22057	Lafourche Parish	2011	22001	Acadia Parish	2012	1
22	2200057	22065	Madison Parish	2012	22001	Acadia Parish	2012	0
22	2200057	22079	Rapides Parish	2012	22001	Acadia Parish	2012	0
22	2200720	22045	Iberia Parish	2012	22045	Iberia Parish	2012	0
22	2200720	22113	Vermilion Parish	2012	22045	Iberia Parish	2012	0

23	2310770	23005	Cumberland County	2012	23005	Cumberland County	2012	0
23	2310770	23017	Oxford County	2012	23005	Cumberland County	2012	0
23	2311550	23013	Knox County	2012	23013	Knox County	2012	0
23	2311550	23015	Lincoln County	2012	23013	Knox County	2012	0
23	2311580	23019	Penobscot County	2012	23019	Penobscot County	2012	0
23	2311580	23021	Piscataquis County	2012	23019	Penobscot County	2012	0
23	2311790	23005	Cumberland County	2012	23005	Cumberland County	2012	0
23	2311790	23031	York County	2012	23005	Cumberland County	2012	0
23	2312100	23003	Aroostook County	2012	23003	Aroostook County	2012	0
23	2312100	23019	Penobscot County	2012	23003	Aroostook County	2012	0
23	2312100	23025	Somerset County	2012	23003	Aroostook County	2012	0
23	2312100	23029	Washington County	2012	23003	Aroostook County	2012	0
23	2314190	23005	Cumberland County	2012	23005	Cumberland County	2012	0
23	2314190	23017	Oxford County	2012	23005	Cumberland County	2012	0
23	2314190	23031	York County	2012	23005	Cumberland County	2012	0
23	2314330	23011	Kennebec County	2012	23011	Kennebec County	2012	0
23	2314330	23025	Somerset County	2012	23011	Kennebec County	2012	0
23	2314768	23005	Cumberland County	2012	23005	Cumberland County	2012	0
23	2314768	23023	Sagadahoc County	2012	23005	Cumberland County	2012	0
23	2314776	23011	Kennebec County	2012	23011	Kennebec County	2012	0
23	2314776	23015	Lincoln County	2012	23011	Kennebec County	2012	0
23	2314776	23023	Sagadahoc County	2012	23011	Kennebec County	2012	0
23	2314780	23011	Kennebec County	2012	23011	Kennebec County	2012	0
23	2314780	23015	Lincoln County	2012	23011	Kennebec County	2012	0
23	2314780	23027	Waldo County	2012	23011	Kennebec County	2012	0
23	2314783	23001	Androscoggin County	2012	23001	Androscoggin County	2012	0
23	2314783	23011	Kennebec County	2012	23001	Androscoggin County	2012	0
23	2314785	23019	Penobscot County	2012	23019	Penobscot County	2012	0
23	2314785	23025	Somerset County	2012	23019	Penobscot County	2012	0
23	2314789	23001	Androscoggin County	2012	23001	Androscoggin County	2012	0
23	2314789	23005	Cumberland County	2012	23001	Androscoggin County	2012	0
23	2314790	23009	Hancock County	2012	23009	Hancock County	2012	0
23	2314790	23029	Washington County	2012	23009	Hancock County	2012	0
23	2314805	23001	Androscoggin County	2012	23001	Androscoggin County	2012	0
23	2314805	23007	Franklin County	2012	23001	Androscoggin County	2012	0
23	2314815	23019	Penobscot County	2012	23019	Penobscot County	2012	0
23	2314815	23027	Waldo County	2012	23019	Penobscot County	2012	0
25	2503030	25005	Bristol County	2012	25005	Bristol County	2012	0
25	2503030	25023	Plymouth County	2012	25005	Bristol County	2012	0
25	2503390	25003	Berkshire County	2012	25003	Berkshire County	2012	0
25	2503390	25015	Hampshire County	2012	25003	Berkshire County	2012	0
25	2505070	25005	Bristol County	2012	25005	Bristol County	2012	0
25	2505070	25023	Plymouth County	2012	25005	Bristol County	2012	0
25	2505160	25013	Hampden County	2012	25013	Hampden County	2012	0
25	2505160	25015	Hampshire County	2012	25013	Hampden County	2012	0
25	2508310	25017	Middlesex County	2012	25017	Middlesex County	2012	0
25	2508310	25027	Worcester County	2012	25017	Middlesex County	2012	0
26	2602790	26099	Macomb County	2011	26099	Macomb County	2011	0
26	2602790	26147	St. Clair County	2011	26099	Macomb County	2011	0
26	2604950	26019	Benzie County	2012	26019	Benzie County	2012	0
26	2604950	26101	Manistee County	2012	26019	Benzie County	2012	0
26	2606270	26005	Allegan County	2011	26005	Allegan County	2011	0
26	2606270	26159	Van Buren County	2012	26005	Allegan County	2011	1
26	2607620	26005	Allegan County	2011	26005	Allegan County	2011	0
26	2607620	26081	Kent County	2011	26005	Allegan County	2011	0
26	2608130	26067	Ionia County	2011	26067	Ionia County	2011	0
26	2608130	26117	Montcalm County	2012	26067	Ionia County	2011	1
26	2609560	26073	Isabella County	2012	26073	Isabella County	2012	0
26	2609560	26107	Mecosta County	2012	26073	Isabella County	2012	0
26	2612150	26027	Cass County	2012	26027	Cass County	2012	0
26	2612150	26159	Van Buren County	2012	26027	Cass County	2012	0
26	2613050	26009	Antrim County	2012	26009	Antrim County	2012	0
26	2613050	26055	Grand Traverse County	2012	26009	Antrim County	2012	0

26	2614570	26055	Grand Traverse County	2012	26055	Grand Traverse County	2012	0
26	2614570	26079	Kalkaska County	2012	26055	Grand Traverse County	2012	0
26	2615600	26033	Chippewa County	2012	26033	Chippewa County	2012	0
26	2615600	26097	Mackinac County	2012	26033	Chippewa County	2012	0
26	2616410	26037	Clinton County	2011	26037	Clinton County	2011	0
26	2616410	26045	Eaton County	2011	26037	Clinton County	2011	0
26	2617160	26081	Kent County	2011	26081	Kent County	2011	0
26	2617160	26117	Montcalm County	2012	26081	Kent County	2011	1
26	2618420	26005	Allegan County	2011	26005	Allegan County	2011	0
26	2618420	26139	Ottawa County	2011	26005	Allegan County	2011	0
26	2618480	26045	Eaton County	2011	26045	Eaton County	2011	0
26	2618480	26065	Ingham County	2011	26045	Eaton County	2011	0
26	2619890	26119	Montmorency County	2012	26119	Montmorency County	2012	0
26	2619890	26137	Otsego County	2012	26119	Montmorency County	2012	0
26	2620280	26081	Kent County	2011	26081	Kent County	2011	0
26	2620280	26139	Ottawa County	2011	26081	Kent County	2011	0
26	2620940	26049	Genesee County	2011	26049	Genesee County	2011	0
26	2620940	26087	Lapeer County	2012	26049	Genesee County	2011	1
26	2620980	26015	Barry County	2011	26015	Barry County	2011	0
26	2620980	26045	Eaton County	2011	26015	Barry County	2011	0
26	2620980	26067	Ionia County	2011	26015	Barry County	2011	0
26	2621150	26037	Clinton County	2011	26037	Clinton County	2011	0
26	2621150	26045	Eaton County	2011	26037	Clinton County	2011	0
26	2621150	26065	Ingham County	2011	26037	Clinton County	2011	0
26	2622620	26015	Barry County	2011	26015	Barry County	2011	0
26	2622620	26045	Eaton County	2011	26015	Barry County	2011	0
26	2623490	26099	Macomb County	2011	26099	Macomb County	2011	0
26	2623490	26147	St. Clair County	2011	26099	Macomb County	2011	0
26	2623850	26115	Monroe County	2011	26115	Monroe County	2011	0
26	2623850	26161	Washtenaw County	2011	26115	Monroe County	2011	0
26	2624180	26121	Muskegon County	2011	26121	Muskegon County	2011	0
26	2624180	26127	Oceana County	2012	26121	Muskegon County	2011	1
26	2625350	26095	Luce County	2012	26095	Luce County	2012	0
26	2625350	26097	Mackinac County	2012	26095	Luce County	2012	0
26	2625560	26021	Berrien County	2012	26021	Berrien County	2012	0
26	2625560	26027	Cass County	2012	26021	Berrien County	2012	0
26	2625980	26125	Oakland County	2011	26125	Oakland County	2011	0
26	2625980	26163	Wayne County	2010	26125	Oakland County	2011	1
26	2626970	26001	Alcona County	2012	26001	Alcona County	2012	0
26	2626970	26069	Iosco County	2012	26001	Alcona County	2012	0
26	2627060	26005	Allegan County	2011	26005	Allegan County	2011	0
26	2627060	26077	Kalamazoo County	2011	26005	Allegan County	2011	0
26	2627150	26037	Clinton County	2011	26037	Clinton County	2011	0
26	2627150	26155	Shiawassee County	2011	26037	Clinton County	2011	0
26	2627960	26037	Clinton County	2011	26037	Clinton County	2011	0
26	2627960	26067	Ionia County	2011	26037	Clinton County	2011	0
26	2628200	26085	Lake County	2012	26085	Lake County	2012	0
26	2628200	26133	Osceola County	2012	26085	Lake County	2012	0
26	2628530	26005	Allegan County	2011	26005	Allegan County	2011	0
26	2628530	26077	Kalamazoo County	2011	26005	Allegan County	2011	0
26	2630090	26099	Macomb County	2011	26099	Macomb County	2011	0
26	2630090	26125	Oakland County	2011	26099	Macomb County	2011	0
26	2632250	26093	Livingston County	2011	26093	Livingston County	2011	0
26	2632250	26125	Oakland County	2011	26093	Livingston County	2011	0
26	2632250	26161	Washtenaw County	2011	26093	Livingston County	2011	0
26	2632300	26005	Allegan County	2011	26005	Allegan County	2011	0
26	2632300	26159	Van Buren County	2012	26005	Allegan County	2011	1
26	2633930	26081	Kent County	2011	26081	Kent County	2011	0
26	2633930	26117	Montcalm County	2012	26081	Kent County	2011	1
26	2634560	26161	Washtenaw County	2011	26161	Washtenaw County	2011	0
26	2634560	26163	Wayne County	2010	26161	Washtenaw County	2011	1
26	2635190	26099	Macomb County	2011	26099	Macomb County	2011	0
26	2635190	26125	Oakland County	2011	26099	Macomb County	2011	0

26	2635520	26045	Eaton County	2011	26045	Eaton County	2011	0
26	2635520	26065	Ingham County	2011	26045	Eaton County	2011	0
27	2700006	27013	Blue Earth County	2012	27013	Blue Earth County	2012	0
27	2700006	27043	Faribault County	2012	27013	Blue Earth County	2012	0
27	2700013	27079	Le Sueur County	2012	27079	Le Sueur County	2012	0
27	2700013	27131	Rice County	2012	27079	Le Sueur County	2012	0
27	2700025	27053	Hennepin County	2012	27053	Hennepin County	2012	0
27	2700025	27123	Ramsey County	2012	27053	Hennepin County	2012	0
27	2700055	27109	Olmsted County	2012	27109	Olmsted County	2012	0
27	2700055	27157	Wabasha County	2012	27109	Olmsted County	2012	0
27	2700055	27169	Winona County	2012	27109	Olmsted County	2012	0
27	2700086	27129	Renville County	2012	27129	Renville County	2012	0
27	2700086	27145	Stearns County	2012	27129	Renville County	2012	0
27	2700089	27147	Steele County	2012	27147	Steele County	2012	0
27	2700089	27161	Waseca County	2012	27147	Steele County	2012	0
27	2700091	27023	Chippewa County	2012	27023	Chippewa County	2012	0
27	2700091	27067	Kandiyohi County	2012	27023	Chippewa County	2012	0
27	2700104	27041	Douglas County	2012	27041	Douglas County	2012	0
27	2700104	27051	Grant County	2012	27041	Douglas County	2012	0
27	2700106	27067	Kandiyohi County	2012	27067	Kandiyohi County	2012	0
27	2700106	27093	Meeker County	2012	27067	Kandiyohi County	2012	0
27	2700107	27089	Marshall County	2012	27089	Marshall County	2012	0
27	2700107	27135	Roseau County	2012	27089	Marshall County	2012	0
27	2700110	27127	Redwood County	2012	27127	Redwood County	2012	0
27	2700110	27129	Renville County	2012	27127	Redwood County	2012	0
27	2700123	27085	Mcleod County	2012	27085	Mcleod County	2012	0
27	2700123	27171	Wright County	2012	27085	Mcleod County	2012	0
27	2700125	27073	Lac Qui Parle County	2012	27073	Lac Qui Parle County	2012	0
27	2700125	27151	Swift County	2012	27073	Lac Qui Parle County	2012	0
27	2700182	27127	Redwood County	2012	27127	Redwood County	2012	0
27	2700182	27129	Renville County	2012	27127	Redwood County	2012	0
27	2700183	27033	Cottonwood County	2012	27033	Cottonwood County	2012	0
27	2700183	27127	Redwood County	2012	27033	Cottonwood County	2012	0
27	2700224	27071	Koochiching County	2012	27071	Koochiching County	2012	0
27	2700224	27137	St. Louis County	2012	27071	Koochiching County	2012	0
27	2700294	27083	Lyon County	2012	27083	Lyon County	2012	0
27	2700294	27117	Pipestone County	2012	27083	Lyon County	2012	0
27	2700328	27011	Big Stone County	2012	27011	Big Stone County	2012	0
27	2700328	27073	Lac Qui Parle County	2012	27011	Big Stone County	2012	0
27	2700379	27079	Le Sueur County	2012	27079	Le Sueur County	2012	0
27	2700379	27131	Rice County	2012	27079	Le Sueur County	2012	0
27	2700383	27121	Pope County	2012	27121	Pope County	2012	0
27	2700383	27149	Stevens County	2012	27121	Pope County	2012	0
27	2700420	27139	Scott County	2012	27139	Scott County	2012	0
27	2700420	27171	Wright County	2012	27139	Scott County	2012	0
27	2702264	27003	Anoka County	2012	27003	Anoka County	2012	0
27	2702264	27053	Hennepin County	2012	27003	Anoka County	2012	0
27	2702264	27123	Ramsey County	2012	27003	Anoka County	2012	0
27	2702264	27163	Washington County	2012	27003	Anoka County	2012	0
27	2703180	27003	Anoka County	2012	27003	Anoka County	2012	0
27	2703180	27053	Hennepin County	2012	27003	Anoka County	2012	0
27	2707290	27037	Dakota County	2012	27037	Dakota County	2012	0
27	2707290	27139	Scott County	2012	27037	Dakota County	2012	0
27	2708220	27045	Fillmore County	2012	27045	Fillmore County	2012	0
27	2708220	27109	Olmsted County	2012	27045	Fillmore County	2012	0
27	2710060	27093	Meeker County	2012	27093	Meeker County	2012	0
27	2710060	27171	Wright County	2012	27093	Meeker County	2012	0
27	2711190	27093	Meeker County	2012	27093	Meeker County	2012	0
27	2711190	27145	Stearns County	2012	27093	Meeker County	2012	0
27	2711370	27053	Hennepin County	2012	27053	Hennepin County	2012	0
27	2711370	27141	Sherburne County	2012	27053	Hennepin County	2012	0
27	2711370	27171	Wright County	2012	27053	Hennepin County	2012	0
27	2711670	27019	Carver County	2012	27019	Carver County	2012	0

27	2711670	27053	Hennepin County	2012	27019	Carver County	2012	0
27	2712270	27003	Anoka County	2012	27003	Anoka County	2012	0
27	2712270	27025	Chisago County	2012	27003	Anoka County	2012	0
27	2712270	27163	Washington County	2012	27003	Anoka County	2012	0
27	2712580	27129	Renville County	2012	27129	Renville County	2012	0
27	2712580	27143	Sibley County	2012	27129	Renville County	2012	0
27	2713590	27039	Dodge County	2012	27039	Dodge County	2012	0
27	2713590	27099	Mower County	2012	27039	Dodge County	2012	0
27	2718070	27079	Le Sueur County	2012	27079	Le Sueur County	2012	0
27	2718070	27143	Sibley County	2012	27079	Le Sueur County	2012	0
27	2718780	27013	Blue Earth County	2012	27013	Blue Earth County	2012	0
27	2718780	27103	Nicollet County	2012	27013	Blue Earth County	2012	0
27	2723490	27015	Brown County	2012	27015	Brown County	2012	0
27	2723490	27103	Nicollet County	2012	27015	Brown County	2012	0
27	2723850	27123	Ramsey County	2012	27123	Ramsey County	2012	0
27	2723850	27163	Washington County	2012	27123	Ramsey County	2012	0
27	2723880	27037	Dakota County	2012	27037	Dakota County	2012	0
27	2723880	27131	Rice County	2012	27037	Dakota County	2012	0
27	2728950	27049	Goodhue County	2012	27049	Goodhue County	2012	0
27	2728950	27109	Olmsted County	2012	27049	Goodhue County	2012	0
27	2728970	27021	Cass County	2012	27021	Cass County	2012	0
27	2728970	27035	Crow Wing County	2012	27021	Cass County	2012	0
27	2732070	27053	Hennepin County	2012	27053	Hennepin County	2012	0
27	2732070	27171	Wright County	2012	27053	Hennepin County	2012	0
27	2733510	27009	Benton County	2012	27009	Benton County	2012	0
27	2733510	27141	Sherburne County	2012	27009	Benton County	2012	0
27	2733510	27145	Stearns County	2012	27009	Benton County	2012	0
27	2733900	27097	Morrison County	2012	27097	Morrison County	2012	0
27	2733900	27153	Todd County	2012	27097	Morrison County	2012	0
27	2740740	27075	Lake County	2012	27075	Lake County	2012	0
27	2740740	27137	St. Louis County	2012	27075	Lake County	2012	0
27	2741850	27077	Lake Of The Woods County	2012	27077	Lake Of The Woods County	2012	0
27	2741850	27135	Roseau County	2012	27077	Lake Of The Woods County	2012	0
27	2742120	27005	Becker County	2012	27005	Becker County	2012	0
27	2742120	27087	Mahnomen County	2012	27005	Becker County	2012	0
27	2742360	27123	Ramsey County	2012	27123	Ramsey County	2012	0
27	2742360	27163	Washington County	2012	27123	Ramsey County	2012	0
27	2745735	27049	Goodhue County	2012	27049	Goodhue County	2012	0
27	2745735	27157	Wabasha County	2012	27049	Goodhue County	2012	0
27	2791451	27013	Blue Earth County	2012	27013	Blue Earth County	2012	0
27	2791451	27161	Waseca County	2012	27013	Blue Earth County	2012	0
28	2800540	28081	Lee County	2012	28081	Lee County	2012	0
28	2800540	28117	Prentiss County	2012	28081	Lee County	2012	0
29	2900001	29073	Gasconade County	2012	29073	Gasconade County	2012	0
29	2900001	29125	Maries County	2012	29073	Gasconade County	2012	0
29	2911250	29021	Buchanan County	2011	29021	Buchanan County	2011	0
29	2911250	29049	Clinton County	2012	29021	Buchanan County	2011	1
29	2911650	29047	Clay County	2011	29047	Clay County	2011	0
29	2911650	29177	Ray County	2012	29047	Clay County	2011	1
29	2912690	29079	Grundy County	2012	29079	Grundy County	2012	0
29	2912690	29211	Sullivan County	2011	29079	Grundy County	2012	1
29	2916190	29027	Callaway County	2012	29027	Callaway County	2012	0
29	2916190	29051	Cole County	2011	29027	Callaway County	2012	1
29	2916350	29097	Jasper County	2011	29097	Jasper County	2011	0
29	2916350	29145	Newton County	2011	29097	Jasper County	2011	0
29	2919170	29077	Greene County	2011	29077	Greene County	2011	0
29	2919170	29225	Webster County	2011	29077	Greene County	2011	0
29	2921120	29009	Barry County	2012	29009	Barry County	2012	0
29	2921120	29109	Lawrence County	2012	29009	Barry County	2012	0
29	2921540	29091	Howell County	2011	29091	Howell County	2011	0
29	2921540	29203	Shannon County	2012	29091	Howell County	2011	1
29	2922560	29087	Holt County	2012	29087	Holt County	2012	0
29	2922560	29147	Nodaway County	2012	29087	Holt County	2012	0

29	2923340	29071	Franklin County	2012	29071	Franklin County	2012	0
29	2923340	29073	Gasconade County	2012	29071	Franklin County	2012	0
29	2929810	29203	Shannon County	2012	29203	Shannon County	2012	0
29	2929810	29215	Texas County	2011	29203	Shannon County	2012	1
29	2931110	29071	Franklin County	2012	29071	Franklin County	2012	0
29	2931110	29183	St. Charles County	2011	29071	Franklin County	2012	1
29	2931110	29219	Warren County	2012	29071	Franklin County	2012	0
30	3000093	30051	Liberty County	2012	30051	Liberty County	2012	0
30	3000093	30101	Toole County	2012	30051	Liberty County	2012	0
30	3000098	30041	Hill County	2012	30041	Hill County	2012	0
30	3000098	30051	Liberty County	2012	30041	Hill County	2012	0
30	3000102	30073	Pondera County	2012	30073	Pondera County	2012	0
30	3000102	30099	Teton County	2012	30073	Pondera County	2012	0
31	3100003	31023	Butler County	2012	31023	Butler County	2012	0
31	3100003	31155	Saunders County	2011	31023	Butler County	2012	1
31	3100018	31029	Chase County	2012	31029	Chase County	2012	0
31	3100018	31087	Hitchcock County	2012	31029	Chase County	2012	0
31	3100021	31025	Cass County	2012	31025	Cass County	2012	0
31	3100021	31109	Lancaster County	2011	31025	Cass County	2012	1
31	3100066	31001	Adams County	2011	31001	Adams County	2011	0
31	3100066	31181	Webster County	2012	31001	Adams County	2011	1
31	3100071	31021	Burt County	2012	31021	Burt County	2012	0
31	3100071	31177	Washington County	2012	31021	Burt County	2012	0
31	3100077	31057	Dundy County	2012	31057	Dundy County	2012	0
31	3100077	31087	Hitchcock County	2012	31057	Dundy County	2012	0
31	3100119	31003	Antelope County	2012	31003	Antelope County	2012	0
31	3100119	31107	Knox County	2012	31003	Antelope County	2012	0
31	3100122	31035	Clay County	2012	31035	Clay County	2012	0
31	3100122	31129	Nuckolls County	2012	31035	Clay County	2012	0
31	3100123	31121	Merrick County	2011	31121	Merrick County	2011	0
31	3100123	31143	Polk County	2012	31121	Merrick County	2011	1
31	3100125	31067	Gage County	2012	31067	Gage County	2012	0
31	3100125	31095	Jefferson County	2012	31067	Gage County	2012	0
31	3100129	31121	Merrick County	2011	31121	Merrick County	2011	0
31	3100129	31125	Nance County	2012	31121	Merrick County	2011	1
31	3100134	31061	Franklin County	2012	31061	Franklin County	2012	0
31	3100134	31099	Kearney County	2012	31061	Franklin County	2012	0
31	3100140	31033	Cheyenne County	2012	31033	Cheyenne County	2012	0
31	3100140	31049	Deuel County	2012	31033	Cheyenne County	2012	0
31	3100141	31051	Dixon County	2012	31051	Dixon County	2012	0
31	3100141	31173	Thurston County	2012	31051	Dixon County	2012	0
31	3100146	31055	Douglas County	2011	31055	Douglas County	2011	0
31	3100146	31079	Hall County	2011	31055	Douglas County	2011	0
31	3100164	31019	Buffalo County	2011	31019	Buffalo County	2011	0
31	3100164	31043	Dakota County	2012	31019	Buffalo County	2011	1
31	3100164	31111	Lincoln County	2012	31019	Buffalo County	2011	1
31	3100164	31141	Platte County	2012	31019	Buffalo County	2011	1
31	3100170	31031	Cherry County	2012	31031	Cherry County	2012	0
31	3100170	31161	Sheridan County	2012	31031	Cherry County	2012	0
31	3100183	31037	Colfax County	2012	31037	Colfax County	2012	0
31	3100183	31053	Dodge County	2011	31037	Colfax County	2012	1
31	3100184	31011	Boone County	2012	31011	Boone County	2012	0
31	3100184	31077	Greeley County	2012	31011	Boone County	2012	0
31	3100185	31077	Greeley County	2012	31077	Greeley County	2012	0
31	3100185	31175	Valley County	2012	31077	Greeley County	2012	0
31	3104950	31109	Lancaster County	2011	31109	Lancaster County	2011	0
31	3104950	31155	Saunders County	2011	31109	Lancaster County	2011	0
31	3170710	31053	Dodge County	2011	31053	Dodge County	2011	0
31	3170710	31155	Saunders County	2011	31053	Dodge County	2011	0
31	3171520	31027	Cedar County	2012	31027	Cedar County	2012	0
31	3171520	31051	Dixon County	2012	31027	Cedar County	2012	0
31	3172210	31075	Grant County	2012	31075	Grant County	2012	0
31	3172210	31161	Sheridan County	2012	31075	Grant County	2012	0

31	3173740	31055	Douglas County	2011	31055	Douglas County	2011	0
31	3173740	31153	Sarpy County	2011	31055	Douglas County	2011	0
31	3174430	31119	Madison County	2011	31119	Madison County	2011	0
31	3174430	31167	Stanton County	2012	31119	Madison County	2011	1
31	3174580	31079	Hall County	2011	31079	Hall County	2011	0
31	3174580	31093	Howard County	2011	31079	Hall County	2011	0
31	3174580	31121	Merrick County	2011	31079	Hall County	2011	0
31	3174820	31055	Douglas County	2011	31055	Douglas County	2011	0
31	3174820	31153	Sarpy County	2011	31055	Douglas County	2011	0
31	3175210	31109	Lancaster County	2011	31109	Lancaster County	2011	0
31	3175210	31131	Otoe County	2012	31109	Lancaster County	2011	1
31	3175770	31043	Dakota County	2012	31043	Dakota County	2012	0
31	3175770	31051	Dixon County	2012	31043	Dakota County	2012	0
31	3175810	31033	Cheyenne County	2012	31033	Cheyenne County	2012	0
31	3175810	31105	Kimball County	2012	31033	Cheyenne County	2012	0
31	3176380	31009	Blaine County	2012	31009	Blaine County	2012	0
31	3176380	31171	Thomas County	2012	31009	Blaine County	2012	0
31	3176590	31023	Butler County	2012	31023	Butler County	2012	0
31	3176590	31143	Polk County	2012	31023	Butler County	2012	0
33	3302480	33005	Cheshire County	2012	33005	Cheshire County	2012	0
33	3302480	33011	Hillsborough County	2012	33005	Cheshire County	2012	0
33	3302990	33005	Cheshire County	2012	33005	Cheshire County	2012	0
33	3302990	33019	Sullivan County	2012	33005	Cheshire County	2012	0
33	3303330	33003	Carroll County	2012	33003	Carroll County	2012	0
33	3303330	33017	Strafford County	2012	33003	Carroll County	2012	0
33	3303960	33001	Belknap County	2012	33001	Belknap County	2012	0
33	3303960	33003	Carroll County	2012	33001	Belknap County	2012	0
33	3305220	33001	Belknap County	2012	33001	Belknap County	2012	0
33	3305220	33009	Grafton County	2012	33001	Belknap County	2012	0
33	3305220	33013	Merrimack County	2012	33001	Belknap County	2012	0
33	3306180	33001	Belknap County	2012	33001	Belknap County	2012	0
33	3306180	33013	Merrimack County	2012	33001	Belknap County	2012	0
33	3307300	33001	Belknap County	2012	33001	Belknap County	2012	0
33	3307300	33013	Merrimack County	2012	33001	Belknap County	2012	0
34	3405760	34003	Bergen County	2012	34003	Bergen County	2012	0
34	3405760	34031	Passaic County	2012	34003	Bergen County	2012	0
34	3417700	34021	Mercer County	2012	34021	Mercer County	2012	0
34	3417700	34023	Middlesex County	2012	34021	Mercer County	2012	0
35	3500060	35001	Bernalillo County	2010	35001	Bernalillo County	2010	0
35	3500060	35043	Sandoval County	2011	35001	Bernalillo County	2010	1
35	3500120	35005	Chaves County	2011	35005	Chaves County	2011	0
35	3500120	35015	Eddy County	2011	35005	Chaves County	2011	0
35	3500180	35053	Socorro County	2011	35053	Socorro County	2011	0
35	3500180	35061	Valencia County	2011	35053	Socorro County	2011	0
35	3500900	35039	Rio Arriba County	2011	35039	Rio Arriba County	2011	0
35	3500900	35049	Santa Fe County	2011	35039	Rio Arriba County	2011	0
35	3501080	35013	Dona Ana County	2010	35013	DoñA Ana County	0	1
35	3501080	35035	Otero County	2011	35013	DoñA Ana County	0	1
35	3501890	35001	Bernalillo County	2010	35001	Bernalillo County	2010	0
35	3501890	35049	Santa Fe County	2011	35001	Bernalillo County	2010	1
35	3501890	35057	Torrance County	2011	35001	Bernalillo County	2010	1
36	3600013	36053	Madison County	2012	36053	Madison County	2012	0
36	3600013	36065	Oneida County	2012	36053	Madison County	2012	0
36	3601107	36035	Fulton County	2011	36035	Fulton County	2011	0
36	3601107	36057	Montgomery County	2012	36035	Fulton County	2011	1
36	3602940	36059	Nassau County	2011	36059	Nassau County	2011	0
36	3602940	36103	Suffolk County	2011	36059	Nassau County	2011	0
36	3605040	36049	Lewis County	2012	36049	Lewis County	2012	0
36	3605040	36065	Oneida County	2012	36049	Lewis County	2012	0
36	3605940	36091	Saratoga County	2012	36091	Saratoga County	2012	0
36	3605940	36093	Schenectady County	2012	36091	Saratoga County	2012	0
36	3606900	36067	Onondaga County	2012	36067	Onondaga County	2012	0
36	3606900	36075	Oswego County	2012	36067	Onondaga County	2012	0

36	3608010	36059	Nassau County	2011	36059	Nassau County	2011	0
36	3608010	36103	Suffolk County	2011	36059	Nassau County	2011	0
36	3608970	36009	Cattaraugus County	2012	36009	Cattaraugus County	2012	0
36	3608970	36121	Wyoming County	2011	36009	Cattaraugus County	2012	1
36	3610710	36007	Broome County	2012	36007	Broome County	2012	0
36	3610710	36107	Tioga County	2012	36007	Broome County	2012	0
36	3610980	36059	Nassau County	2011	36059	Nassau County	2011	0
36	3610980	36103	Suffolk County	2011	36059	Nassau County	2011	0
36	3612300	36069	Ontario County	2011	36069	Ontario County	2011	0
36	3612300	36123	Yates County	2011	36069	Ontario County	2011	0
36	3612390	36009	Cattaraugus County	2012	36009	Cattaraugus County	2012	0
36	3612390	36029	Erie County	2012	36009	Cattaraugus County	2012	0
36	3616170	36019	Clinton County	2011	36019	Clinton County	2011	0
36	3616170	36031	Essex County	2012	36019	Clinton County	2011	1
36	3620580	36005	Bronx County	2012	36005	Bronx County	2012	0
36	3620580	36047	Kings County	2012	36005	Bronx County	2012	0
36	3620580	36061	New York County	2012	36005	Bronx County	2012	0
36	3620580	36081	Queens County	2012	36005	Bronx County	2012	0
36	3620580	36085	Richmond County	2012	36005	Bronx County	2012	0
36	3620880	36091	Saratoga County	2012	36091	Saratoga County	2012	0
36	3620880	36093	Schenectady County	2012	36091	Saratoga County	2012	0
36	3622100	36017	Chenango County	2012	36017	Chenango County	2012	0
36	3622100	36053	Madison County	2012	36017	Chenango County	2012	0
36	3625740	36031	Essex County	2012	36031	Essex County	2012	0
36	3625740	36033	Franklin County	2012	36031	Essex County	2012	0
36	3627240	36091	Saratoga County	2012	36091	Saratoga County	2012	0
36	3627240	36119	Westchester County	2011	36091	Saratoga County	2012	1
36	3627750	36015	Chemung County	2012	36015	Chemung County	2012	0
36	3627750	36107	Tioga County	2012	36015	Chemung County	2012	0
36	3629610	36007	Broome County	2012	36007	Broome County	2012	0
36	3629610	36107	Tioga County	2012	36007	Broome County	2012	0
36	3629790	36071	Orange County	2011	36071	Orange County	2011	0
36	3629790	36111	Ulster County	2011	36071	Orange County	2011	0
36	3630270	36015	Chemung County	2012	36015	Chemung County	2012	0
36	3630270	36107	Tioga County	2012	36015	Chemung County	2012	0
37	3702430	37025	Cabarrus County	2009	37025	Cabarrus County	2009	0
37	3702430	37159	Rowan County	2011	37025	Cabarrus County	2009	1
37	3703270	37065	Edgecombe County	2011	37065	Edgecombe County	2011	0
37	3703270	37127	Nash County	2011	37065	Edgecombe County	2011	0
38	3800042	38031	Foster County	2012	38031	Foster County	2012	0
38	3800042	38039	Griggs County	2012	38031	Foster County	2012	0
38	3800057	38003	Barnes County	2012	38003	Barnes County	2012	0
38	3800057	38045	Lamoure County	2012	38003	Barnes County	2012	0
38	3800058	38061	Mountrail County	2012	38061	Mountrail County	2012	0
38	3800058	38101	Ward County	2012	38061	Mountrail County	2012	0
38	3800060	38003	Barnes County	2012	38003	Barnes County	2012	0
38	3800060	38093	Stutsman County	2012	38003	Barnes County	2012	0
38	3800397	38067	Pembina County	2012	38067	Pembina County	2012	0
38	3800397	38099	Walsh County	2012	38067	Pembina County	2012	0
38	3812020	38003	Barnes County	2012	38003	Barnes County	2012	0
38	3812020	38017	Cass County	2012	38003	Barnes County	2012	0
38	3812350	38055	Mclean County	2012	38055	Mclean County	2012	0
38	3812350	38101	Ward County	2012	38055	Mclean County	2012	0
38	3812930	38073	Ransom County	2012	38073	Ransom County	2012	0
38	3812930	38081	Sargent County	2012	38073	Ransom County	2012	0
38	3813200	38015	Burleigh County	2012	38015	Burleigh County	2012	0
38	3813200	38055	Mclean County	2012	38015	Burleigh County	2012	0
38	3813400	38079	Rolette County	2012	38079	Rolette County	2012	0
38	3813400	38095	Towner County	2012	38079	Rolette County	2012	0
39	3904359	39043	Erie County	2012	39043	Erie County	2012	0
39	3904359	39077	Huron County	2011	39043	Erie County	2012	1
39	3904359	39143	Sandusky County	2011	39043	Erie County	2012	1
39	3904388	39003	Allen County	2011	39003	Allen County	2011	0

39	3904388	39161	Van Wert County	2011	39003	Allen County	2011	0
39	3904399	39147	Seneca County	2011	39147	Seneca County	2011	0
39	3904399	39173	Wood County	2011	39147	Seneca County	2011	0
39	3904504	39041	Delaware County	2011	39041	Delaware County	2011	0
39	3904504	39049	Franklin County	2010	39041	Delaware County	2011	1
39	3904528	39035	Cuyahoga County	2010	39035	Cuyahoga County	2010	0
39	3904528	39055	Geauga County	2011	39035	Cuyahoga County	2010	1
39	3904540	39071	Highland County	2011	39071	Highland County	2011	0
39	3904540	39141	Ross County	2011	39071	Highland County	2011	0
39	3904700	39049	Franklin County	2010	39049	Franklin County	2010	0
39	3904700	39089	Licking County	2011	39049	Franklin County	2010	1
39	3904702	39041	Delaware County	2011	39041	Delaware County	2011	0
39	3904702	39049	Franklin County	2010	39041	Delaware County	2011	1
39	3904702	39159	Union County	2011	39041	Delaware County	2011	0
39	3904768	39075	Holmes County	2012	39075	Holmes County	2012	0
39	3904768	39169	Wayne County	2011	39075	Holmes County	2012	1
39	3904800	39049	Franklin County	2010	39049	Franklin County	2010	0
39	3904800	39089	Licking County	2011	39049	Franklin County	2010	1
39	3904838	39029	Columbiana County	2012	39029	Columbiana County	2012	0
39	3904838	39099	Mahoning County	2012	39029	Columbiana County	2012	0
39	3904865	39013	Belmont County	2011	39013	Belmont County	2011	0
39	3904865	39111	Monroe County	2011	39013	Belmont County	2011	0
39	3904883	39059	Guernsey County	2011	39059	Guernsey County	2011	0
39	3904883	39119	Muskingum County	2011	39059	Guernsey County	2011	0
39	3904884	39119	Muskingum County	2011	39119	Muskingum County	2011	0
39	3904884	39127	Perry County	2011	39119	Muskingum County	2011	0
39	3904888	39089	Licking County	2011	39089	Licking County	2011	0
39	3904888	39119	Muskingum County	2011	39089	Licking County	2011	0
39	3904908	39073	Hocking County	2011	39073	Hocking County	2011	0
39	3904908	39129	Pickaway County	2011	39073	Hocking County	2011	0
39	3904957	39123	Ottawa County	2011	39123	Ottawa County	2011	0
39	3904957	39143	Sandusky County	2011	39123	Ottawa County	2011	0
39	3904989	39029	Columbiana County	2012	39029	Columbiana County	2012	0
39	3904989	39151	Stark County	2011	39029	Columbiana County	2012	1
39	3905041	39113	Montgomery County	2011	39113	Montgomery County	2011	0
39	3905041	39165	Warren County	2011	39113	Montgomery County	2011	0
39	3905058	39075	Holmes County	2012	39075	Holmes County	2012	0
39	3905058	39169	Wayne County	2011	39075	Holmes County	2012	1
40	4000782	40063	Hughes County	2012	40063	Hughes County	2012	0
40	4000782	40107	Okfuskee County	2011	40063	Hughes County	2012	1
40	4002720	40003	Alfalfa County	2012	40003	Alfalfa County	2012	0
40	4002720	40093	Major County	2012	40003	Alfalfa County	2012	0
40	4003840	40001	Adair County	2011	40001	Adair County	2011	0
40	4003840	40135	Sequoyah County	2011	40001	Adair County	2011	0
40	4005490	40143	Tulsa County	2010	40143	Tulsa County	2010	0
40	4005490	40145	Wagoner County	2011	40143	Tulsa County	2010	1
40	4015690	40041	Delaware County	2011	40041	Delaware County	2011	0
40	4015690	40097	Mayes County	2011	40041	Delaware County	2011	0
40	4019290	40125	Pottawatomie County	2011	40125	Pottawatomie County	2011	0
40	4019290	40133	Seminole County	2012	40125	Pottawatomie County	2011	1
40	4019950	40027	Cleveland County	2011	40027	Cleveland County	2011	0
40	4019950	40109	Oklahoma County	2010	40027	Cleveland County	2011	1
40	4020250	40027	Cleveland County	2011	40027	Cleveland County	2011	0
40	4020250	40109	Oklahoma County	2010	40027	Cleveland County	2011	1
40	4023280	40131	Rogers County	2011	40131	Rogers County	2011	0
40	4023280	40143	Tulsa County	2010	40131	Rogers County	2011	1
40	4027750	40113	Osage County	2011	40113	Osage County	2011	0
40	4027750	40143	Tulsa County	2010	40113	Osage County	2011	1
40	4030240	40113	Osage County	2011	40113	Osage County	2011	0
40	4030240	40143	Tulsa County	2010	40113	Osage County	2011	1
41	4100020	41043	Linn County	2012	41043	Linn County	2012	0
41	4100020	41047	Marion County	2010	41043	Linn County	2012	1
41	4101120	41003	Benton County	2012	41003	Benton County	2012	0

41	4101120	41043	Linn County	2012	41003	Benton County	2012	0
41	4101230	41053	Polk County	2012	41053	Polk County	2012	0
41	4101230	41071	Yamhill County	2012	41053	Polk County	2012	0
41	4103720	41013	Crook County	2012	41013	Crook County	2012	0
41	4103720	41017	Deschutes County	2012	41013	Crook County	2012	0
41	4106000	41005	Clackamas County	2010	41005	Clackamas County	2010	0
41	4106000	41051	Multnomah County	2010	41005	Clackamas County	2010	0
41	4106740	41031	Jefferson County	2012	41031	Jefferson County	2012	0
41	4106740	41065	Wasco County	2012	41031	Jefferson County	2012	0
41	4106900	41029	Jackson County	2012	41029	Jackson County	2012	0
41	4106900	41033	Josephine County	2012	41029	Jackson County	2012	0
41	4110040	41051	Multnomah County	2010	41051	Multnomah County	2010	0
41	4110040	41067	Washington County	2011	41051	Multnomah County	2010	1
41	4110820	41047	Marion County	2010	41047	Marion County	2010	0
41	4110820	41053	Polk County	2012	41047	Marion County	2010	1
41	4110980	41009	Columbia County	2011	41009	Columbia County	2011	0
41	4110980	41051	Multnomah County	2010	41009	Columbia County	2011	1
41	4111450	41005	Clackamas County	2010	41005	Clackamas County	2010	0
41	4111450	41047	Marion County	2010	41005	Clackamas County	2010	0
41	4112240	41005	Clackamas County	2010	41005	Clackamas County	2010	0
41	4112240	41067	Washington County	2011	41005	Clackamas County	2010	1
42	4203210	42051	Fayette County	2012	42051	Fayette County	2012	0
42	4203210	42129	Westmoreland County	2012	42051	Fayette County	2012	0
42	4203480	42037	Columbia County	2012	42037	Columbia County	2012	0
42	4203480	42079	Luzerne County	2012	42037	Columbia County	2012	0
42	4203570	42077	Lehigh County	2012	42077	Lehigh County	2012	0
42	4203570	42095	Northampton County	2012	42077	Lehigh County	2012	0
42	4203750	42063	Indiana County	2012	42063	Indiana County	2012	0
42	4203750	42129	Westmoreland County	2012	42063	Indiana County	2012	0
42	4203960	42011	Berks County	2012	42011	Berks County	2012	0
42	4203960	42091	Montgomery County	2009	42011	Berks County	2012	1
42	4206860	42039	Crawford County	2012	42039	Crawford County	2012	0
42	4206860	42049	Erie County	2012	42039	Crawford County	2012	0
42	4207320	42093	Montour County	2012	42093	Montour County	2012	0
42	4207320	42097	Northumberland County	2012	42093	Montour County	2012	0
42	4207830	42033	Clearfield County	2012	42033	Clearfield County	2012	0
42	4207830	42065	Jefferson County	2012	42033	Clearfield County	2012	0
42	4208670	42089	Monroe County	2012	42089	Monroe County	2012	0
42	4208670	42103	Pike County	2012	42089	Monroe County	2012	0
42	4209600	42005	Armstrong County	2012	42005	Armstrong County	2012	0
42	4209600	42019	Butler County	2012	42005	Armstrong County	2012	0
42	4210440	42005	Armstrong County	2012	42005	Armstrong County	2012	0
42	4210440	42019	Butler County	2012	42005	Armstrong County	2012	0
42	4211700	42079	Luzerne County	2012	42079	Luzerne County	2012	0
42	4211700	42107	Schuylkill County	2012	42079	Luzerne County	2012	0
42	4212300	42039	Crawford County	2012	42039	Crawford County	2012	0
42	4212300	42085	Mercer County	2012	42039	Crawford County	2012	0
42	4212480	42035	Clinton County	2012	42035	Clinton County	2012	0
42	4212480	42081	Lycoming County	2012	42035	Clinton County	2012	0
42	4212725	42027	Centre County	2012	42027	Centre County	2012	0
42	4212725	42035	Clinton County	2012	42027	Centre County	2012	0
42	4212840	42005	Armstrong County	2012	42005	Armstrong County	2012	0
42	4212840	42129	Westmoreland County	2012	42005	Armstrong County	2012	0
42	4215480	42097	Northumberland County	2012	42097	Northumberland County	2012	0
42	4215480	42119	Union County	2012	42097	Northumberland County	2012	0
42	4216020	42061	Huntingdon County	2012	42061	Huntingdon County	2012	0
42	4216020	42087	Mifflin County	2012	42061	Huntingdon County	2012	0
42	4219020	42027	Centre County	2012	42027	Centre County	2012	0
42	4219020	42033	Clearfield County	2012	42027	Centre County	2012	0
42	4219800	42063	Indiana County	2012	42063	Indiana County	2012	0
42	4219800	42065	Jefferson County	2012	42063	Indiana County	2012	0
42	4219830	42033	Clearfield County	2012	42033	Clearfield County	2012	0
42	4219830	42063	Indiana County	2012	42033	Clearfield County	2012	0

42	4220130	42005	Armstrong County	2012	42005	Armstrong County	2012	0
42	4220130	42031	Clarion County	2012	42005	Armstrong County	2012	0
42	4221570	42041	Cumberland County	2012	42041	Cumberland County	2012	0
42	4221570	42055	Franklin County	2012	42041	Cumberland County	2012	0
42	4222560	42029	Chester County	2012	42029	Chester County	2012	0
42	4222560	42091	Montgomery County	2009	42029	Chester County	2012	1
42	4223490	42039	Crawford County	2012	42039	Crawford County	2012	0
42	4223490	42121	Venango County	2012	42039	Crawford County	2012	0
42	4223970	42009	Bedford County	2012	42009	Bedford County	2012	0
42	4223970	42061	Huntingdon County	2012	42009	Bedford County	2012	0
42	4224000	42011	Berks County	2012	42011	Berks County	2012	0
42	4224000	42029	Chester County	2012	42011	Berks County	2012	0
42	4224540	42011	Berks County	2012	42011	Berks County	2012	0
42	4224540	42091	Montgomery County	2009	42011	Berks County	2012	1
42	4224750	42103	Pike County	2012	42103	Pike County	2012	0
42	4224750	42127	Wayne County	2012	42103	Pike County	2012	0
42	4225830	42041	Cumberland County	2012	42041	Cumberland County	2012	0
42	4225830	42133	York County	2012	42041	Cumberland County	2012	0
42	4226400	42043	Dauphin County	2012	42043	Dauphin County	2012	0
42	4226400	42107	Schuylkill County	2012	42043	Dauphin County	2012	0
42	4226700	42015	Bradford County	2012	42015	Bradford County	2012	0
42	4226700	42131	Wyoming County	2012	42015	Bradford County	2012	0
44	4400360	44003	Kent County	2011	44003	Kent County	2011	0
44	4400360	44009	Washington County	2011	44003	Kent County	2011	0
45	4500720	45003	Aiken County	2012	45003	Aiken County	2012	0
45	4500720	45081	Saluda County	2012	45003	Aiken County	2012	0
45	4502370	45047	Greenwood County	2011	45047	Greenwood County	2011	0
45	4502370	45059	Laurens County	2011	45047	Greenwood County	2011	0
45	4502820	45063	Lexington County	2012	45063	Lexington County	2012	0
45	4502820	45079	Richland County	2011	45063	Lexington County	2012	1
46	4600042	46065	Hughes County	2012	46065	Hughes County	2012	0
46	4600042	46119	Sully County	2012	46065	Hughes County	2012	0
46	4600046	46005	Beadle County	2012	46005	Beadle County	2012	0
46	4600046	46115	Spink County	2012	46005	Beadle County	2012	0
46	4600052	46083	Lincoln County	2012	46083	Lincoln County	2012	0
46	4600052	46099	Minnehaha County	2011	46083	Lincoln County	2012	1
46	4600053	46091	Marshall County	2012	46091	Marshall County	2012	0
46	4600053	46109	Roberts County	2012	46091	Marshall County	2012	0
46	4601027	46077	Kingsbury County	2012	46077	Kingsbury County	2012	0
46	4601027	46079	Lake County	2012	46077	Kingsbury County	2012	0
46	4614130	46095	Mellette County	2012	46095	Mellette County	2012	0
46	4614130	46123	Tripp County	2012	46095	Mellette County	2012	0
46	4620100	46041	Dewey County	2012	46041	Dewey County	2012	0
46	4620100	46137	Ziebach County	2012	46041	Dewey County	2012	0
46	4621420	46061	Hanson County	2012	46061	Hanson County	2012	0
46	4621420	46087	Mccook County	2012	46061	Hanson County	2012	0
46	4636150	46005	Beadle County	2012	46005	Beadle County	2012	0
46	4636150	46077	Kingsbury County	2012	46005	Beadle County	2012	0
46	4641550	46083	Lincoln County	2012	46083	Lincoln County	2012	0
46	4641550	46125	Turner County	2012	46083	Lincoln County	2012	0
46	4647100	46067	Hutchinson County	2012	46067	Hutchinson County	2012	0
46	4647100	46135	Yankton County	2012	46067	Hutchinson County	2012	0
46	4648390	46035	Davison County	2012	46035	Davison County	2012	0
46	4648390	46061	Hanson County	2012	46035	Davison County	2012	0
46	4651750	46013	Brown County	2012	46013	Brown County	2012	0
46	4651750	46115	Spink County	2012	46013	Brown County	2012	0
46	4659820	46093	Meade County	2012	46093	Meade County	2012	0
46	4659820	46103	Pennington County	2011	46093	Meade County	2012	1
46	4669930	46081	Lawrence County	2012	46081	Lawrence County	2012	0
46	4669930	46093	Meade County	2012	46081	Lawrence County	2012	0
46	4672450	46023	Charles Mix County	2012	46023	Charles Mix County	2012	0
46	4672450	46043	Douglas County	2012	46023	Charles Mix County	2012	0
46	4672450	46067	Hutchinson County	2012	46023	Charles Mix County	2012	0

46	4678300	46011	Brookings County	2012	46011	Brookings County	2012	0
46	4678300	46039	Deuel County	2012	46011	Brookings County	2012	0
46	4679350	46005	Beadle County	2012	46005	Beadle County	2012	0
46	4679350	46025	Clark County	2012	46005	Beadle County	2012	0
46	4680437	46055	Haakon County	2012	46055	Haakon County	2012	0
46	4680437	46071	Jackson County	2011	46055	Haakon County	2012	1
46	4680438	46015	Brule County	2012	46015	Brule County	2012	0
46	4680438	46023	Charles Mix County	2012	46015	Brule County	2012	0
46	4680439	46027	Clay County	2012	46027	Clay County	2012	0
46	4680439	46125	Turner County	2012	46027	Clay County	2012	0
46	4680441	46021	Campbell County	2012	46021	Campbell County	2012	0
46	4680441	46129	Walworth County	2012	46021	Campbell County	2012	0
46	4680445	46003	Aurora County	2012	46003	Aurora County	2012	0
46	4680445	46043	Douglas County	2012	46003	Aurora County	2012	0
47	4702190	47073	Hawkins County	2012	47073	Hawkins County	2012	0
47	4702190	47163	Sullivan County	2011	47073	Hawkins County	2012	1
47	4704200	47031	Coffee County	2011	47031	Coffee County	2011	0
47	4704200	47051	Franklin County	2012	47031	Coffee County	2011	1
48	4808130	48375	Potter County	2010	48375	Potter County	2010	0
48	4808130	48381	Randall County	2010	48375	Potter County	2010	0
48	4809200	48367	Parker County	2011	48367	Parker County	2011	0
48	4809200	48439	Tarrant County	2010	48367	Parker County	2011	1
48	4809300	48399	Runnels County	2011	48399	Runnels County	2011	0
48	4809300	48451	Tom Green County	2011	48399	Runnels County	2011	0
48	4809450	48071	Chambers County	2011	48071	Chambers County	2011	0
48	4809450	48201	Harris County	2009	48071	Chambers County	2011	1
48	4810710	48029	Bexar County	2010	48029	Bexar County	2010	0
48	4810710	48259	Kendall County	2011	48029	Bexar County	2010	1
48	4812180	48251	Johnson County	2009	48251	Johnson County	2009	0
48	4812180	48439	Tarrant County	2010	48251	Johnson County	2009	1
48	4813050	48113	Dallas County	2009	48113	Dallas County	2009	0
48	4813050	48121	Denton County	2009	48113	Dallas County	2009	0
48	4814280	48167	Galveston County	2010	48167	Galveston County	2010	0
48	4814280	48201	Harris County	2009	48167	Galveston County	2010	1
48	4814700	48015	Austin County	2011	48015	Austin County	2011	0
48	4814700	48089	Colorado County	2011	48015	Austin County	2011	0
48	4814730	48029	Bexar County	2010	48029	Bexar County	2010	0
48	4814730	48091	Comal County	2011	48029	Bexar County	2010	1
48	4814730	48187	Guadalupe County	2011	48029	Bexar County	2010	1
48	4816200	48111	Dallam County	2011	48111	Dallam County	2011	0
48	4816200	48205	Hartley County	2011	48111	Dallam County	2011	0
48	4816950	48163	Frio County	2011	48163	Frio County	2011	0
48	4816950	48325	Medina County	2011	48163	Frio County	2011	0
48	4817850	48029	Bexar County	2010	48029	Bexar County	2010	0
48	4817850	48493	Wilson County	2011	48029	Bexar County	2010	1
48	4818070	48095	Concho County	2011	48095	Concho County	2011	0
48	4818070	48451	Tom Green County	2011	48095	Concho County	2011	0
48	4818360	48021	Bastrop County	2011	48021	Bastrop County	2011	0
48	4818360	48453	Travis County	2010	48021	Bastrop County	2011	1
48	4819050	48119	Delta County	2011	48119	Delta County	2011	0
48	4819050	48147	Fannin County	2011	48119	Delta County	2011	0
48	4820010	48085	Collin County	2009	48085	Collin County	2009	0
48	4820010	48121	Denton County	2009	48085	Collin County	2009	0
48	4821150	48071	Chambers County	2011	48071	Chambers County	2011	0
48	4821150	48201	Harris County	2009	48071	Chambers County	2011	1
48	4822410	48199	Hardin County	2011	48199	Hardin County	2011	0
48	4822410	48245	Jefferson County	2010	48199	Hardin County	2011	1
48	4824150	48239	Jackson County	2011	48239	Jackson County	2011	0
48	4824150	48469	Victoria County	2011	48239	Jackson County	2011	0
48	4825170	48157	Fort Bend County	2010	48157	Fort Bend County	2010	0
48	4825170	48201	Harris County	2009	48157	Fort Bend County	2010	1
48	4825170	48473	Waller County	2011	48157	Fort Bend County	2010	1
48	4825620	48183	Gregg County	2011	48183	Gregg County	2011	0

48	4825620	48401	Rusk County	2011	48183	Gregg County	2011	0
48	4825660	48027	Bell County	2010	48027	Bell County	2010	0
48	4825660	48099	Coryell County	2011	48027	Bell County	2010	1
48	4825870	48207	Haskell County	2011	48207	Haskell County	2011	0
48	4825870	48275	Knox County	2011	48207	Haskell County	2011	0
48	4826310	48177	Gonzales County	2011	48177	Gonzales County	2011	0
48	4826310	48493	Wilson County	2011	48177	Gonzales County	2011	0
48	4827030	48453	Travis County	2010	48453	Travis County	2010	0
48	4827030	48491	Williamson County	2010	48453	Travis County	2010	0
48	4828650	48013	Atascosa County	2011	48013	Atascosa County	2011	0
48	4828650	48325	Medina County	2011	48013	Atascosa County	2011	0
48	4828680	48213	Henderson County	2011	48213	Henderson County	2011	0
48	4828680	48257	Kaufman County	2009	48213	Henderson County	2011	1
48	4828920	48251	Johnson County	2009	48251	Johnson County	2009	0
48	4828920	48439	Tarrant County	2010	48251	Johnson County	2009	1
48	4831920	48049	Brown County	2011	48049	Brown County	2011	0
48	4831920	48219	Hockley County	2011	48049	Brown County	2011	0
48	4831920	48303	Lubbock County	2010	48049	Brown County	2011	1
48	4831920	48333	Mills County	2011	48049	Brown County	2011	0
48	4831920	48411	San Saba County	2011	48049	Brown County	2011	0
48	4832370	48091	Comal County	2011	48091	Comal County	2011	0
48	4832370	48187	Guadalupe County	2011	48091	Comal County	2011	0
48	4833180	48121	Denton County	2009	48121	Denton County	2009	0
48	4833180	48439	Tarrant County	2010	48121	Denton County	2009	1
48	4833180	48497	Wise County	2011	48121	Denton County	2009	1
48	4833240	48137	Edwards County	2011	48137	Edwards County	2011	0
48	4833240	48385	Real County	2009	48137	Edwards County	2011	1
48	4833980	48095	Concho County	2011	48095	Concho County	2011	0
48	4833980	48451	Tom Green County	2011	48095	Concho County	2011	0
48	4836000	48085	Collin County	2009	48085	Collin County	2009	0
48	4836000	48121	Denton County	2009	48085	Collin County	2009	0
48	4837380	48081	Coke County	2011	48081	Coke County	2011	0
48	4837380	48451	Tom Green County	2011	48081	Coke County	2011	0
48	4837860	48335	Mitchell County	2011	48335	Mitchell County	2011	0
48	4837860	48353	Nolan County	2011	48335	Mitchell County	2011	0
48	4838080	48453	Travis County	2010	48453	Travis County	2010	0
48	4838080	48491	Williamson County	2010	48453	Travis County	2010	0
48	4838220	48085	Collin County	2009	48085	Collin County	2009	0
48	4838220	48231	Hunt County	2009	48085	Collin County	2009	0
48	4838220	48397	Rockwall County	2011	48085	Collin County	2009	1
48	4839480	48029	Bexar County	2010	48029	Bexar County	2010	0
48	4839480	48187	Guadalupe County	2011	48029	Bexar County	2010	1
48	4841550	48431	Sterling County	2011	48431	Sterling County	2011	0
48	4841550	48451	Tom Green County	2011	48431	Sterling County	2011	0
48	4842960	48201	Harris County	2009	48201	Harris County	2009	0
48	4842960	48339	Montgomery County	2010	48201	Harris County	2009	1
48	4843720	48463	Uvalde County	2009	48463	Uvalde County	2009	0
48	4843720	48507	Zavala County	2009	48463	Uvalde County	2009	0
48	4843760	48035	Bosque County	2011	48035	Bosque County	2011	0
48	4843760	48309	Mclennan County	2010	48035	Bosque County	2011	1
48	4844430	48201	Harris County	2009	48201	Harris County	2009	0
48	4844430	48473	Waller County	2011	48201	Harris County	2009	1
48	4844470	48015	Austin County	2011	48015	Austin County	2011	0
48	4844470	48157	Fort Bend County	2010	48015	Austin County	2011	1
48	4846620	48123	Dewitt County	2011	48123	Dewitt County	2011	0
48	4846620	48285	Lavaca County	2011	48123	Dewitt County	2011	0
50	5000384	50017	Orange County	2012	33009	Grafton County	0	1
50	5000385	50021	Rutland County	2012	50021	Rutland County	2012	0
50	5000385	50027	Windsor County	2012	50021	Rutland County	2012	0
50	5000404	50017	Orange County	2012	50017	Orange County	2012	0
50	5000404	50023	Washington County	2012	50017	Orange County	2012	0
50	5000407	50005	Caledonia County	2012	50005	Caledonia County	2012	0
50	5000407	50009	Essex County	2012	50005	Caledonia County	2012	0

50	5099904	50001	Addison County	2012	50001	Addison County	2012	0
50	5099904	50021	Rutland County	2012	50001	Addison County	2012	0
50	5099906	50003	Bennington County	2012	50003	Bennington County	2012	0
50	5099906	50021	Rutland County	2012	50003	Bennington County	2012	0
50	5099906	50025	Windham County	2012	50003	Bennington County	2012	0
50	5099930	50001	Addison County	2012	50001	Addison County	2012	0
50	5099930	50017	Orange County	2012	50001	Addison County	2012	0
50	5099930	50027	Windsor County	2012	50001	Addison County	2012	0
50	5099931	50009	Essex County	2012	50009	Essex County	2012	0
50	5099931	50019	Orleans County	2012	50009	Essex County	2012	0
50	5099935	50005	Caledonia County	2012	50005	Caledonia County	2012	0
50	5099935	50015	Lamoille County	2012	50005	Caledonia County	2012	0
50	5099935	50019	Orleans County	2012	50005	Caledonia County	2012	0
50	5099935	50023	Washington County	2012	50005	Caledonia County	2012	0
50	5099936	50001	Addison County	2012	50001	Addison County	2012	0
50	5099936	50021	Rutland County	2012	50001	Addison County	2012	0
50	5099949	50003	Bennington County	2012	50003	Bennington County	2012	0
50	5099949	50025	Windham County	2012	50003	Bennington County	2012	0
50	5099951	50021	Rutland County	2012	50021	Rutland County	2012	0
50	5099951	50027	Windsor County	2012	50021	Rutland County	2012	0
51	5100090	51003	Albemarle County	2011	51003	Albemarle County	2011	0
51	5100090	51540	Charlottesville City	2011	51003	Albemarle County	2011	0
51	5101260	51059	Fairfax County	2009	51059	Fairfax County	2009	0
51	5101260	51600	Fairfax City	2009	51059	Fairfax County	2009	0
51	5101470	51069	Frederick County	2011	51069	Frederick County	2011	0
51	5101470	51840	Winchester City	2011	51069	Frederick County	2011	0
51	5101740	51081	Greensville County	2010	51081	Greensville County	2010	0
51	5101740	51595	Emporia City	2010	51081	Greensville County	2010	0
51	5103240	51087	Henrico County	2010	51087	Henrico County	2010	0
51	5103240	51760	Richmond City	2010	51087	Henrico County	2010	0
51	5103330	51161	Roanoke County	2011	51161	Roanoke County	2011	0
51	5103330	51770	Roanoke City	2011	51161	Roanoke County	2011	0
51	5103370	51163	Rockbridge County	2011	51163	Rockbridge County	2011	0
51	5103370	51678	Lexington City	2011	51163	Rockbridge County	2011	0
51	5103390	51165	Rockingham County	2011	51165	Rockingham County	2011	0
51	5103390	51660	Harrisonburg City	2011	51165	Rockingham County	2011	0
51	5104020	51095	James City County	2011	51095	James City County	2011	0
51	5104020	51830	Williamsburg City	2011	51095	James City County	2011	0
53	5300005	53011	Clark County	2012	53011	Clark County	2012	0
53	5300005	53015	Cowlitz County	2012	53011	Clark County	2012	0
53	5300007	53009	Clallam County	2012	53009	Clallam County	2012	0
53	5300007	53035	Kitsap County	2012	53009	Clallam County	2012	0
53	5300008	53057	Skagit County	2012	53057	Skagit County	2012	0
53	5300008	53061	Snohomish County	2012	53057	Skagit County	2012	0
53	5300008	53073	Whatcom County	2012	53057	Skagit County	2012	0
53	5300300	53033	King County	2012	53033	King County	2012	0
53	5300300	53053	Pierce County	2012	53033	King County	2012	0
53	5303130	53025	Grant County	2012	53025	Grant County	2012	0
53	5303130	53047	Okanogan County	2012	53025	Grant County	2012	0
53	5305640	53063	Spokane County	2012	53063	Spokane County	2012	0
53	5305640	53065	Stevens County	2012	53063	Spokane County	2012	0
53	5305910	53033	King County	2012	53033	King County	2012	0
53	5305910	53061	Snohomish County	2012	53033	King County	2012	0
53	5308340	53029	Island County	2012	53029	Island County	2012	0
53	5308340	53061	Snohomish County	2012	53029	Island County	2012	0
53	5308970	53011	Clark County	2012	53011	Clark County	2012	0
53	5308970	53077	Yakima County	2012	53011	Clark County	2012	0
53	5309540	53011	Clark County	2012	53011	Clark County	2012	0
53	5309540	53059	Skamania County	2012	53011	Clark County	2012	0
53	5310140	53053	Pierce County	2012	53053	Pierce County	2012	0
53	5310140	53067	Thurston County	2012	53053	Pierce County	2012	0
55	5500058	55003	Ashland County	2011	55003	Ashland County	2011	0
55	5500058	55099	Price County	2011	55003	Ashland County	2011	0

55	5500061	55005	Barron County	2011	55005	Barron County	2011	0
55	5500061	55107	Rusk County	2011	55005	Barron County	2011	0
55	5500360	55067	Langlade County	2011	55067	Langlade County	2011	0
55	5500360	55115	Shawano County	2011	55067	Langlade County	2011	0
55	5500390	55015	Calumet County	2011	55015	Calumet County	2011	0
55	5500390	55087	Outagamie County	2011	55015	Calumet County	2011	0
55	5500870	55005	Barron County	2011	55005	Barron County	2011	0
55	5500870	55033	Dunn County	2011	55005	Barron County	2011	0
55	5500900	55003	Ashland County	2011	55003	Ashland County	2011	0
55	5500900	55007	Bayfield County	2011	55003	Ashland County	2011	0
55	5501140	55047	Green Lake County	2011	55047	Green Lake County	2011	0
55	5501140	55137	Waushara County	2011	55047	Green Lake County	2011	0
55	5501310	55049	Iowa County	2011	55049	Iowa County	2011	0
55	5501310	55065	Lafayette County	2011	55049	Iowa County	2011	0
55	5501890	55101	Racine County	2011	55101	Racine County	2011	0
55	5501890	55127	Walworth County	2011	55101	Racine County	2011	0
55	5507170	55053	Jackson County	2011	55053	Jackson County	2011	0
55	5507170	55055	Jefferson County	2011	55053	Jackson County	2011	0
55	5507470	55015	Calumet County	2011	55015	Calumet County	2011	0
55	5507470	55087	Outagamie County	2011	55015	Calumet County	2011	0
55	5508640	55007	Bayfield County	2011	55007	Bayfield County	2011	0
55	5508640	55031	Douglas County	2011	55007	Bayfield County	2011	0
55	5509000	55053	Jackson County	2011	55053	Jackson County	2011	0
55	5509000	55063	La Crosse County	2011	55053	Jackson County	2011	0
55	5509210	55069	Lincoln County	2011	55069	Lincoln County	2011	0
55	5509210	55073	Marathon County	2011	55069	Lincoln County	2011	0
55	5510170	55101	Racine County	2011	55101	Racine County	2011	0
55	5510170	55133	Waukesha County	2011	55101	Racine County	2011	0
55	5510590	55087	Outagamie County	2011	55087	Outagamie County	2011	0
55	5510590	55135	Waupaca County	2011	55087	Outagamie County	2011	0
55	5510890	55055	Jefferson County	2011	55055	Jefferson County	2011	0
55	5510890	55133	Waukesha County	2011	55055	Jefferson County	2011	0
55	5511100	55025	Dane County	2011	55025	Dane County	2011	0
55	5511100	55045	Green County	2011	55025	Dane County	2011	0
55	5511220	55035	Eau Claire County	2011	55035	Eau Claire County	2011	0
55	5511220	55121	Trempealeau County	2011	55035	Eau Claire County	2011	0
55	5511350	55055	Jefferson County	2011	55055	Jefferson County	2011	0
55	5511350	55133	Waukesha County	2011	55055	Jefferson County	2011	0
55	5512060	55021	Columbia County	2011	55021	Columbia County	2011	0
55	5512060	55077	Marquette County	2011	55021	Columbia County	2011	0
55	5512330	55009	Brown County	2011	55009	Brown County	2011	0
55	5512330	55083	Oconto County	2011	55009	Brown County	2011	0
55	5513650	55071	Manitowoc County	2011	55071	Manitowoc County	2011	0
55	5513650	55117	Sheboygan County	2011	55071	Manitowoc County	2011	0
55	5514250	55049	Iowa County	2011	55049	Iowa County	2011	0
55	5514250	55103	Richland County	2011	55049	Iowa County	2011	0
55	5514250	55111	Sauk County	2011	55049	Iowa County	2011	0
55	5514910	55057	Juneau County	2011	55057	Juneau County	2011	0
55	5514910	55081	Monroe County	2011	55057	Juneau County	2011	0
55	5515750	55027	Dodge County	2011	55027	Dodge County	2011	0
55	5515750	55055	Jefferson County	2011	55027	Dodge County	2011	0
55	5515870	55027	Dodge County	2011	55027	Dodge County	2011	0
55	5515870	55039	Fond Du Lac County	2011	55027	Dodge County	2011	0
55	5516260	55079	Milwaukee County	2011	55079	Milwaukee County	2011	0
55	5516260	55133	Waukesha County	2011	55079	Milwaukee County	2011	0
55	5516440	55077	Marquette County	2011	55077	Marquette County	2011	0
55	5516440	55137	Waushara County	2011	55077	Marquette County	2011	0
55	5517040	55021	Columbia County	2011	55021	Columbia County	2011	0
55	5517040	55077	Marquette County	2011	55021	Columbia County	2011	0
55	5517040	55111	Sauk County	2011	55021	Columbia County	2011	0
55	5517070	55097	Portage County	2011	55097	Portage County	2011	0
55	5517070	55141	Wood County	2011	55097	Portage County	2011	0
55	5517100	55073	Marathon County	2011	55073	Marathon County	2011	0

55	5517100	55115	Shawano County	2011	55073	Marathon County	2011	0
56	5601030	56007	Carbon County	2012	56007	Carbon County	2012	0
56	5601030	56037	Sweetwater County	2012	56007	Carbon County	2012	0
56	5601260	56023	Lincoln County	2012	56023	Lincoln County	2012	0
56	5601260	56035	Sublette County	2012	56023	Lincoln County	2012	0
