# Supplementary material for: Apprehension and educational outcomes among Hispanic students in the United States: The impact of Secure Communities
Source: PLoS One. 2022 Oct 24;17(10):e0276636. doi: 10.1371/journal.pone.0276636 (PMC9591052; doi:10.1371/journal.pone.0276636)
Supplement: S2 Table — Data from SEDA 2009–18 and DHS. Standard two-way fixed effects specifications are estimated with clustered standard errors at the county level and precision weights. * p < 0.05, ** p < 0.01, *** p < 0.001 (two-tailed). (PDF) [file pone.0276636.s006.pdf]

S2 Table. Estimated associations between Secure Communities and the proportion of Hispanic students in the school district for grades 3-8.

	Grade 3	Grade 4	Grade 5
Secure Communities	0.001	0.002	0.001
	(0.002)	(0.001)	(0.001)
Controls for other policies	Yes	Yes	Yes
School district level controls	Yes	Yes	Yes
School district FE	Yes	Yes	Yes
Year FE	Yes	Yes	Yes
Adjusted R^2	0.989	0.989	0.990
N	29,283	29,235	29,075
	Grade 6	Grade 7	Grade 8
Secure Communities	-0.001	-0.001	-0.001
	(0.001)	(0.001)	(0.001)
Controls for other policies	Yes	Yes	Yes
School district level controls	Yes	Yes	Yes
School district FE	Yes	Yes	Yes
Year FE	Yes	Yes	Yes
Adjusted R^2	0.990	0.989	0.989
N	28,782	28,406	27,967

Data from SEDA 2009-18 and DHS. Standard two-way fixed effects specifications are estimated with clustered standard errors at the county level and precision weights.
* $p < 0.05$, ** $p < 0.01$, *** $p < 0.001$ (two-tailed)
