# Supplementary material for: Apprehension and educational outcomes among Hispanic students in the United States: The impact of Secure Communities
Source: PLoS One. 2022 Oct 24;17(10):e0276636. doi: 10.1371/journal.pone.0276636 (PMC9591052; doi:10.1371/journal.pone.0276636)
Supplement: S3 Table — Estimated associations between the implementation of Secure Communities and school district level English language arts achievement among (A) Hispanic, (B) white, and (C) black students. Data from SEDA 2009–18 and DHS. Precision weighted estimates are based on Eq 1. Models 1–4 show results obtained from standard two-way fixed effect specifications. Model 5 shows results obtained using the method outlined by Sun and Abraham (2021). Clustered standard errors at the county level are in parentheses. a Considering that these are state-level policies, there is no variation once we include state-by-year fixed effects. * p < 0.05, ** p < 0.01, *** p < 0.001 (two-tailed). (PDF) [file pone.0276636.s007.pdf]

S3 Table. Estimated associations between the implementation of Secure Communities and school district level English language arts achievement among (A) Hispanic, (B) white, and (C) black students.

A. Hispanic	(1)	(2)	(3)	(4)	(5)
Secure Communities	-0.030***	-0.030*	-0.012*	-0.011*	-0.024***
	(0.007)	(0.014)	(0.005)	(0.005)	(0.007)
**Controls for other policies**					
E-verify	0.056***	omitted[a]	0.041***	0.030**	0.038***
	(0.011)		(0.011)	(0.011)	(0.012)
Omnibus Immigration Laws	-0.007	omitted[a]	-0.002	0.009	0.006
	(0.016)		(0.016)	(0.017)	(0.015)
287(g) state-level agreements	-0.142***	omitted[a]	-0.175***	-0.171***	-0.187***
	(0.018)		(0.012)	(0.016)	(0.016)
287(g) county-level agreements	0.061***	0.061***	0.030	0.022	0.023
	(0.016)	(0.016)	(0.026)	(0.020)	(0.017)
Sanctuary jurisdictions	0.005	-0.005	0.031**	0.019*	0.014
	(0.015)	(0.016)	(0.011)	(0.009)	(0.009)
**School district characteristics**					
% Free/reduced lunch	-0.384***	-0.359***	-0.343***	-0.038	-0.035
	(0.035)	(0.037)	(0.029)	(0.023)	(0.023)
% Special education	-0.895***	-1.059***	-0.764***	0.352***	0.342***
	(0.117)	(0.131)	(0.120)	(0.101)	(0.103)
% English language learner	-0.443***	-0.517***	-0.329***	-0.104***	-0.095***
	(0.051)	(0.059)	(0.067)	(0.023)	(0.024)
SES compsite score	0.042***	0.044***	0.083***	0.010	0.013
	(0.010)	(0.011)	(0.010)	(0.011)	(0.011)
State FE	Yes				
State × Year FE		Yes			
County FE			Yes		
School district FE				Yes	Yes
Year FE	Yes	Yes	Yes	Yes	Yes
Constant	0.103***	0.098***	0.040	-0.315***	-0.309***
	(0.025)	(0.025)	(0.023)	(0.020)	(0.020)
Adjusted R^2	0.566	0.585	0.706	0.866	0.866
N	29,735	29,735	29,735	29,735	29,735

Continued

S3 Table. Continued

B. White	(1)	(2)	(3)	(4)	(5)
Secure Communities	0.001	0.004	-0.006	-0.004	-0.007
	(0.009)	(0.015)	(0.005)	(0.004)	(0.005)
**Controls for other policies**					
E-verify	0.032***	omitted[a]	0.026***	0.008	0.010
	(0.009)		(0.008)	(0.007)	(0.007)
Omnibus Immigration Laws	0.027*	omitted[a]	0.011	0.009	0.009
	(0.013)		(0.013)	(0.011)	(0.011)
287(g) state-level agreements	-0.010	omitted[a]	-0.019	-0.008	-0.013
	(0.019)		(0.019)	(0.011)	(0.011)
287(g) county-level agreements	0.027	0.027	0.011	0.009	0.008
	(0.023)	(0.023)	(0.021)	(0.016)	(0.016)
Sanctuary jurisdictions	0.063***	0.070***	0.006	0.002	0.002
	(0.016)	(0.019)	(0.007)	(0.006)	(0.006)
**School district characteristics**					
% Free/reduced lunch	-0.363***	-0.397***	-0.360***	-0.106***	-0.103***
	(0.035)	(0.039)	(0.030)	(0.016)	(0.016)
% Special education	-0.684***	-0.777***	-0.553***	0.134	0.125
	(0.115)	(0.128)	(0.109)	(0.069)	(0.069)
% English language learner	0.462***	0.516***	0.274***	-0.143***	-0.137***
	(0.066)	(0.074)	(0.078)	(0.032)	(0.032)
SES compsite score	0.148***	0.141***	0.136***	0.019*	0.019***
	(0.009)	(0.010)	(0.010)	(0.008)	(0.008)
State FE	Yes				
State × Year FE		Yes			
County FE			Yes		
School district FE				Yes	Yes
Year FE	Yes	Yes	Yes	Yes	Yes
Constant	0.415***	0.442***	0.431***	0.294***	0.296***
	(0.024)	(0.026)	(0.022)	(0.013)	(0.013)
Adjusted R^2	0.608	0.618	0.767	0.917	0.918
N	27,500	27,500	27,500	27,500	27,500

Continued

S3 Table. Continued

C. Black	(1)	(2)	(3)	(4)	(5)
Secure Communities	-0.001	-0.006	-0.001	-0.001	-0.008
	(0.011)	(0.016)	(0.008)	(0.008)	(0.009)
**Controls for other policies**					
E-verify	0.084***	omitted[a]	0.076***	0.068***	0.074***
	(0.011)		(0.011)	(0.010)	(0.010)
Omnibus Immigration Laws	-0.059***	omitted[a]	-0.068***	-0.067***	-0.066***
	(0.014)		(0.013)	(0.012)	(0.012)
287(g) state-level agreements	-0.058	omitted[a]	-0.074**	-0.076**	-0.081***
	(0.031)		(0.026)	(0.025)	(0.025)
287(g) county-level agreements	0.053	0.052	0.014	0.022	0.019
	(0.031)	(0.032)	(0.035)	(0.028)	(0.030)
Sanctuary jurisdictions	0.023	0.033	-0.006	-0.015	-0.018
	(0.020)	(0.023)	(0.015)	(0.014)	(0.014)
**School district characteristics**					
% Free/reduced lunch	-0.356***	-0.381***	-0.288***	-0.078**	-0.076**
	(0.041)	(0.047)	(0.036)	(0.030)	(0.030)
% Special education	-0.472**	-0.518**	-0.387**	0.230*	0.235*
	(0.162)	(0.180)	(0.134)	(0.102)	(0.102)
% English language learner	-0.267***	-0.259**	-0.381***	-0.201**	-0.189**
	(0.084)	(0.096)	(0.083)	(0.066)	(0.066)
SES compsite score	0.079***	0.076***	0.091***	0.029*	0.030*
	(0.009)	(0.009)	(0.009)	(0.013)	(0.013)
State FE	Yes				
State × Year FE		Yes			
County FE			Yes		
School district FE				Yes	Yes
Year FE	Yes	Yes	Yes	Yes	Yes
Constant	-0.094**	-0.089**	-0.120***	-0.358***	-0.355***
	(0.030)	(0.033)	(0.027)	(0.024)	(0.024)
Adjusted R^2	0.581	0.598	0.777	0.868	0.868
N	15,078	15,078	15,078	15,078	15,078

Data from SEDA 2009-18 and DHS. Precision weighted estimates are based on Equation 1. Models 1-4 show results obtained from standard two-way fixed effect specifications. Model 5 shows results obtained using the method outlined by Sun and Abraham (2021). Clustered standard errors at the county level are in parentheses. [a] Considering that these are state-level policies, there is no variation once we include state-by-year fixed effects. * p < 0.05, ** p < 0.01, *** p < 0.001 (two-tailed)
