# Supplementary material for: Apprehension and educational outcomes among Hispanic students in the United States: The impact of Secure Communities
Source: PLoS One. 2022 Oct 24;17(10):e0276636. doi: 10.1371/journal.pone.0276636 (PMC9591052; doi:10.1371/journal.pone.0276636)
Supplement: S4 Table — Estimated associations between the implementation of Secure Communities and school district level math achievement among (A) Hispanic, (B) white, and (C) black students. Data from SEDA 2009–18 and DHS. Precision weighted estimates are based on Eq 1. Models 1–4 show results obtained from standard two-way fixed effect specifications. Model 5 shows results obtained using the method outlined by Sun and Abraham (2021). Clustered standard errors at the county level are in parentheses. a Considering that these are state-level policies, there is no variation once we include state-by-year fixed effects. * p < 0.05, ** p < 0.01, *** p < 0.001 (two-tailed). (PDF) [file pone.0276636.s008.pdf]

S4 Table. Estimated associations between the implementation of Secure Communities and school district level math achievement among (A) Hispanic, (B) white, and (C) black students.

A. Hispanic	(1)	(2)	(3)	(4)	(5)
Secure Communities	-0.014	-0.017	-0.001	-0.002	-0.016
	(0.010)	(0.015)	(0.007)	(0.007)	(0.009)
**Controls for other policies**					
E-verify	0.037*	omitted[a]	0.028	0.024	0.029
	(0.016)		(0.015)	(0.015)	(0.015)
Omnibus Immigration Laws	0.028*	omitted[a]	0.031*	0.037**	0.035**
	(0.013)		(0.013)	(0.013)	(0.012)
287(g) state-level agreements	-0.077**	omitted[a]	-0.113***	-0.108***	-0.119***
	(0.026)		(0.017)	(0.025)	(0.041)
287(g) county-level agreements	0.056***	0.057***	-0.022	-0.036	-0.036
	(0.017)	(0.017)	(0.037)	(0.045)	(0.041)
Sanctuary jurisdictions	-0.014	-0.015	-0.004	-0.014	-0.018
	(0.013)	(0.015)	(0.010)	(0.011)	(0.012)
**School district characteristics**					
% Free/reduced lunch	-0.321***	-0.318***	-0.264***	-0.009	-0.007
	(0.038)	(0.045)	(0.028)	(0.032)	(0.032)
% Special education	-0.897***	-1.017***	-0.732***	0.085	0.079
	(0.166)	(0.187)	(0.164)	(0.119)	(0.120)
% English language learner	-0.286***	-0.313***	-0.213***	-0.139***	-0.133***
	(0.055)	(0.067)	(0.060)	(0.023)	(0.024)
SES compsite score	0.042***	0.042***	0.083***	0.052***	0.053***
	(0.011)	(0.012)	(0.009)	(0.013)	(0.013)
State FE	Yes				
State × Year FE		Yes			
County FE			Yes		
School district FE				Yes	Yes
Year FE	Yes	Yes	Yes	Yes	Yes
Constant	0.041	0.052	-0.016	-0.276***	-0.268***
	(0.030)	(0.032)	(0.026)	(0.025)	(0.025)
Adjusted R^2	0.500	0.517	0.656	0.818	0.818
N	29,679	29,679	29,679	29,679	29,679

Continued

S4 Table. Continued

B. White	(1)	(2)	(3)	(4)	(5)
Secure Communities	0.003	0.018	-0.003	-0.001	-0.009
	(0.009)	(0.015)	(0.006)	(0.005)	(0.006)
**Controls for other policies**					
E-verify	0.015	omitted[a]	0.014	-0.002	0.001
	(0.013)		(0.012)	(0.012)	(0.012)
Omnibus Immigration Laws	0.042**	omitted[a]	0.027*	0.024*	0.024*
	(0.014)		(0.013)	(0.012)	(0.012)
287(g) state-level agreements	-0.004	omitted[a]	-0.014	-0.008	-0.014
	(0.026)		(0.026)	(0.021)	(0.021)
287(g) county-level agreements	0.034	0.034	-0.023	-0.030	-0.031
	(0.023)	(0.023)	(0.025)	(0.026)	(0.025)
Sanctuary jurisdictions	0.056***	0.065***	-0.003	-0.008	-0.009
	(0.016)	(0.018)	(0.008)	(0.009)	(0.009)
**School district characteristics**					
% Free/reduced lunch	-0.402***	-0.422***	-0.387***	-0.119***	-0.117***
	(0.040)	(0.044)	(0.033)	(0.021)	(0.021)
% Special education	-0.849***	-0.979***	-0.749***	0.154	0.150
	(0.127)	(0.140)	(0.120)	(0.094)	(0.094)
% English language learner	0.469***	0.514***	0.324***	-0.189***	-0.184***
	(0.057)	(0.063)	(0.067)	(0.036)	(0.037)
SES compsite score	0.145***	0.141***	0.142***	0.048***	0.048***
	(0.010)	(0.010)	(0.010)	(0.011)	(0.011)
State FE	Yes				
State × Year FE		Yes			
County FE			Yes		
School district FE				Yes	Yes
Year FE	Yes	Yes	Yes	Yes	Yes
Constant	0.433***	0.452***	0.443***	0.267***	0.272***
	(0.027)	(0.029)	(0.024)	(0.017)	(0.017)
Adjusted R^2	0.602	0.619	0.755	0.894	0.894
N	27,394	27,394	27,394	27,394	27,394

Continued

S4 Table. Continued

C. Black	(1)	(2)	(3)	(4)	(5)
Secure Communities	-0.004	-0.005	-0.003	-0.001	-0.011
	(0.013)	(0.018)	(0.007)	(0.007)	(0.009)
**Controls for other policies**					
E-verify	0.040**	omitted[a]	0.037*	0.030*	0.036*
	(0.015)		(0.015)	(0.015)	(0.015)
Omnibus Immigration Laws	-0.027	omitted[a]	-0.034*	-0.033*	-0.032*
	(0.016)		(0.015)	(0.015)	(0.015)
287(g) state-level agreements	-0.019	omitted[a]	-0.033	-0.039	-0.046*
	(0.027)		(0.023)	(0.023)	(0.022)
287(g) county-level agreements	0.041	0.042	-0.028	-0.019	-0.023
	(0.032)	(0.032)	(0.035)	(0.036)	(0.034)
Sanctuary jurisdictions	-0.009	0.006	-0.028	-0.036*	-0.039*
	(0.021)	(0.025)	(0.015)	(0.015)	(0.015)
**School district characteristics**					
% Free/reduced lunch	-0.316***	-0.330***	-0.250***	-0.069*	-0.068*
	(0.043)	(0.050)	(0.035)	(0.030)	(0.030)
% Special education	-0.413*	-0.473*	-0.391**	0.234	0.237
	(0.172)	(0.198)	(0.148)	(0.135)	(0.136)
% English language learner	-0.292***	-0.296**	-0.392***	-0.232***	-0.217***
	(0.083)	(0.097)	(0.084)	(0.065)	(0.065)
SES compsite score	0.081***	0.079***	0.094***	0.072***	0.072***
	(0.009)	(0.010)	(0.008)	(0.016)	(0.015)
State FE	Yes				
State × Year FE		Yes			
County FE			Yes		
School district FE				Yes	Yes
Year FE	Yes	Yes	Yes	Yes	Yes
Constant	-0.196***	-0.185***	-0.215***	-0.423***	-0.418***
	(0.030)	(0.034)	(0.027)	(0.027)	(0.027)
Adjusted R^2	0.556	0.580	0.749	0.835	0.836
N	14,925	14,925	14,925	14,925	14,925

Data from SEDA 2009-18 and DHS. Precision weighted estimates are based on Equation 1. Models 1-4 show results obtained from standard two-way fixed effect specifications. Model 5 shows results obtained using the method outlined by Sun and Abraham (2021). Clustered standard errors at the county level are in parentheses. [a] Considering that these are state-level policies, there is no variation once we include state-by-year fixed effects. * $p < 0.05$, ** $p < 0.01$, *** $p < 0.001$ (two-tailed)
