# Supplementary material for: Apprehension and educational outcomes among Hispanic students in the United States: The impact of Secure Communities
Source: PLoS One. 2022 Oct 24;17(10):e0276636. doi: 10.1371/journal.pone.0276636 (PMC9591052; doi:10.1371/journal.pone.0276636)
Supplement: S5 Table — Estimated associations between the implementation of Secure Communities and school district level (A) English language arts and (B) math achievement among Hispanic, white, and black students relative to the implementation year. Data from SEDA 2009–18 and DHS. Precision weighted estimates are based on Eq 2. Results are obtained using the method outlined by Sun and Abraham (2021). Clustered standard errors at the county level are in parentheses. * p < 0.05, ** p < 0.01, *** p < 0.001 (two-tailed). (PDF) [file pone.0276636.s009.pdf]

S5 Table. Estimated associations between the implementation of Secure Communities and school district level (A) English language arts and (B) math achievement among Hispanic, white, and black students relative to the implementation year.

A. English language arts	Hispanic	White	Black
T-4	0.014	0.026*	0.006
	(0.013)	(0.012)	(0.031)
T-3	0.008	-0.001	-0.005
	(0.008)	(0.007)	(0.012)
T-2	0.010*	0.003	-0.004
	(0.004)	(0.003)	(0.006)
T-1	0	0	0
T	-0.003	0.004	-0.003
	(0.004)	(0.003)	(0.006)
T+1	-0.015*	0.001	-0.016
	(0.006)	(0.006)	(0.010)
T+2	-0.030***	-0.011	-0.016
	(0.008)	(0.007)	(0.013)
T+3	-0.028**	-0.017*	-0.017
	(0.009)	(0.008)	(0.015)
T+4	-0.012	-0.015	-0.011
	(0.009)	(0.008)	(0.018)
T+5	-0.029**	-0.016	-0.018
	(0.010)	(0.009)	(0.017)
T+6	-0.050***	-0.025*	-0.016
	(0.013)	(0.010)	(0.018)
T+7	-0.039*	-0.019	-0.018
	(0.019)	(0.012)	(0.020)
**Controls for other policies**			
E-verify	0.038***	0.010	0.073***
	(0.012)	(0.007)	(0.010)
Omnibus Immigration Laws	0.006	0.010	-0.064***
	(0.015)	(0.011)	(0.012)
287(g) state-level agreements	-0.202***	-0.020	-0.085**
	(0.017)	(0.013)	(0.028)
287(g) county-level agreements	0.022	0.008	0.018
	(0.014)	(0.016)	(0.029)
Sanctuary jurisdictions	0.011	0.001	-0.021
	(0.009)	(0.007)	(0.015)
**School district characteristics**			
% Free/reduced lunch	-0.035	-0.102***	-0.075*
	(0.025)	(0.016)	(0.030)
% Special education	0.316**	0.117	0.228*
	(0.107)	(0.069)	(0.102)
% English language learner	-0.101***	-0.147***	-0.211***
	(0.025)	(0.033)	(0.066)
SES composite score	0.013	0.018*	0.032*
	(0.012)	(0.008)	(0.013)
School district FE	Yes	Yes	Yes
Year FE	Yes	Yes	Yes
Constant	-0.302***	0.301***	-0.348***
	(0.021)	(0.013)	(0.025)
Adjusted R^2	0.867	0.918	0.869
N	29,735	27,500	15,078

Contined

S5 Table. Continued

B. Math	Hispanic	White	Black
T-4	0.049**	0.006	-0.006
	(0.017)	(0.015)	(0.043)
T-3	0.028**	0.012	0.008
	(0.010)	(0.008)	(0.016)
T-2	0.015**	0.007	-0.003
	(0.006)	(0.004)	(0.008)
T-1	0	0	0
T	-0.001	0.012**	-0.002
	(0.006)	(0.004)	(0.006)
T+1	-0.001	0.008	-0.013
	(0.008)	(0.007)	(0.010)
T+2	-0.019*	-0.001	-0.018
	(0.010)	(0.008)	(0.012)
T+3	-0.024	-0.014	-0.031*
	(0.013)	(0.011)	(0.015)
T+4	-0.010	-0.026*	-0.023
	(0.015)	(0.013)	(0.017)
T+5	-0.019	-0.029	-0.033
	(0.015)	(0.015)	(0.019)
T+6	-0.033*	-0.036*	-0.032
	(0.014)	(0.016)	(0.021)
T+7	-0.025	-0.032	-0.046*
	(0.016)	(0.017)	(0.022)
**Controls for other policies**			
E-verify	0.029	0.001	0.034*
	(0.015)	(0.013)	(0.015)
Omnibus Immigration Laws	0.033**	0.025*	-0.028
	(0.012)	(0.012)	(0.015)
287(g) state-level agreements	-0.141***	-0.031	-0.059*
	(0.026)	(0.022)	(0.025)
287(g) county-level agreements	-0.038	-0.030	-0.026
	(0.036)	(0.025)	(0.033)
Sanctuary jurisdictions	-0.021	-0.013	-0.045**
	(0.012)	(0.010)	(0.016)
**School district characteristics**			
% Free/reduced lunch	-0.003	-0.111***	-0.067*
	(0.032)	(0.022)	(0.030)
% Special education	0.052	0.147	0.223
	(0.125)	(0.095)	(0.138)
% English language learner	-0.125***	-0.186***	-0.236***
	(0.026)	(0.039)	(0.065)
SES composite score	0.054***	0.047***	0.074***
	(0.013)	(0.011)	(0.016)
School district FE	Yes	Yes	Yes
Year FE	Yes	Yes	Yes
Constant	-0.265***	0.276***	-0.403***
	(0.026)	(0.018)	(0.029)
Adjusted R^2	0.819	0.895	0.836
N	29,679	27,394	14,925

Data from SEDA 2009-18 and DHS. Precision weighted estimates are based on Equation 2. Results are obtained using the method outlined by Sun and Abraham (2021). Clustered standard errors at the county level are in parentheses. * $p < 0.05$, ** $p < 0 .01$, *** $p < 0.001$ (two-tailed)
