# Supplementary material for: Apprehension and educational outcomes among Hispanic students in the United States: The impact of Secure Communities
Source: PLoS One. 2022 Oct 24;17(10):e0276636. doi: 10.1371/journal.pone.0276636 (PMC9591052; doi:10.1371/journal.pone.0276636)
Supplement: S6 Table — Data from SEDA 2009–18 and DHS. After sample restrictions, there are four cohorts: 2010, 2011, 2012, 2013. The first treated cohort is used as the control group. (PDF) [file pone.0276636.s010.pdf]

S6 Table. Weights for each relative time period obtained in the Sun and Abraham (2021) method.

A. English language arts	Hispanic			White			Black		
	2011	2012	2013	2011	2012	2013	2011	2012	2013
T-4	0	0	1	0	0	1	0	0	1
T-3	0	0.824	0.176	0	0.906	0.094	0	0.815	0.185
T-2	0.556	0.378	0.066	0.503	0.460	0.037	0.547	0.392	0.061
T	0.550	0.398	0.053	0.516	0.452	0.032	0.579	0.371	0.050
T+1	0.575	0.363	0.062	0.558	0.403	0.039	0.632	0.308	0.059
T+2	0.629	0.313	0.058	0.567	0.393	0.040	0.605	0.344	0.052
T+3	0.651	0.255	0.093	0.615	0.332	0.053	0.680	0.244	0.076
T+4	0.544	0.391	0.065	0.538	0.421	0.040	0.566	0.371	0.063
T+5	0.600	0.335	0.065	0.591	0.370	0.039	0.623	0.312	0.065
T+6	0.561	0.439	0	0.534	0.466	0	0.592	0.408	0
T+7	1	0	0	1	0	0	1	0	0
	Hispanic			White			Black		
B. Math	2011	2012	2013	2011	2012	2013	2011	2012	2013
T-4	0	0	1	0	0	1	0	0	1
T-3	0	0.831	0.169	0	0.910	0.090	0	0.816	0.184
T-2	0.568	0.374	0.058	0.502	0.466	0.032	0.550	0.396	0.054
T	0.547	0.404	0.048	0.509	0.461	0.030	0.573	0.382	0.045
T+1	0.583	0.362	0.055	0.578	0.387	0.035	0.632	0.314	0.053
T+2	0.624	0.326	0.050	0.587	0.379	0.034	0.585	0.367	0.049
T+3	0.648	0.264	0.088	0.612	0.339	0.050	0.672	0.254	0.074
T+4	0.564	0.373	0.063	0.546	0.416	0.038	0.569	0.370	0.062
T+5	0.611	0.326	0.064	0.600	0.362	0.039	0.622	0.315	0.064
T+6	0.561	0.439	0	0.526	0.474	0	0.587	0.413	0
T+7	1	0	0	1	0	0	1	0	0

Data from SEDA 2009-18 and DHS. After sample restrictions, there are four cohorts: 2010, 2011, 2012, 2013. The first treated cohort is used as the control group.
