# Supplementary material for: Apprehension and educational outcomes among Hispanic students in the United States: The impact of Secure Communities
Source: PLoS One. 2022 Oct 24;17(10):e0276636. doi: 10.1371/journal.pone.0276636 (PMC9591052; doi:10.1371/journal.pone.0276636)
Supplement: S7 Table — Estimated associations between Secure Communities and school district level English language arts achievement among Hispanic students across (A) census regions, (B) rural-urban areas, and (C) the proportion of likely undocumented migrants. Data from SEDA 2009–18, DHS, and CPS. Precision weighted estimates are based on Eq 1. Results are obtained using the method outlined by Sun and Abraham (2021). Clustered standard errors at the county level are in parentheses. Urban areas exclude sanctuary jurisdictions. a No policy identified in the states. * p < 0.05, ** p < 0.01, *** p < 0.001 (two-tailed). (PDF) [file pone.0276636.s011.pdf]

S7 Table. Estimated associations between Secure Communities and school district level English language arts achievement among Hispanic students across (A) census regions, (B) rural-urban areas, and (C) the proportion of likely undocumented migrants.

A. Census region	Northeast	Midwest	South	West
Secure Communities	-0.008	-0.014	-0.027*	-0.004
	(0.010)	(0.008)	(0.011)	(0.009)
**Controls for other policies**				
E-verify	omitted [a]	omitted [a]	0.099***	omitted [a]
			(0.010)	
Omnibus Immigration Laws	omitted [a]	0.028	-0.118***	0.064***
		(0.020)	(0.020)	(0.014)
287(g) state-level agreements	omitted [a]	omitted [a]	-0.153***	omitted [a]
			(0.019)	
287(g) county-level agreements	0.037	omitted [a]	0.070***	omitted [a]
	(0.025)		(0.020)	
Sanctuary jurisdictions	-0.022	0.019	-0.028	0.009
	(0.015)	(0.011)	(0.021)	(0.010)
**School district characteristics**				
% Free/reduced lunch	-0.188**	-0.045	0.045	-0.042
	(0.066)	(0.038)	(0.033)	(0.035)
% Special education	-0.160	0.301	0.478***	0.152
	(0.222)	(0.218)	(0.143)	(0.199)
% English language learner	-0.029	-0.343***	-0.451***	-0.075**
	(0.245)	(0.095)	(0.131)	(0.024)
SES composite score	0.052	0.061***	-0.001	0.017
	(0.027)	(0.016)	(0.015)	(0.017)
School district FE	Yes	Yes	Yes	Yes
Year FE	Yes	Yes	Yes	Yes
Constant	-0.072	-0.280***	-0.256***	-0.427***
	(0.058)	(0.040)	(0.029)	(0.030)
Adjusted R^2	0.884	0.847	0.841	0.865
N	5,200	7,502	9,639	7,394

Continued

S7 Table. Continued

B. Rural-urban areas	Sanctuary jurisdiction	Urban	Surburb	Town	Rural
Secure Communities	-0.012	-0.027	-0.021	-0.028**	-0.040**
	(0.014)	(0.015)	(0.012)	(0.012)	(0.015)
**Controls for other policies**					
E-verify	0.010	0.049	-0.56	-0.026	0.088***
	(0.019)	(0.041)	(0.035)	(0.051)	(0.018)
Omnibus Immigration Laws	omitted [a]	0.033	0.018	0.044	-0.073*
		(0.028)	(0.017)	(0.026)	(0.034)
287(g) state-level agreements	omitted [a]	-0.161***	-0.219***	-0.112	-0.340***
		(0.016)	(0.021)	(0.146)	(0.018)
287(g) county-level agreements	omitted [a]	0.023	0.025	omitted [a]	omitted [a]
		(0.018)	(0.025)		
**School district characteristics**					
% Free/reduced lunch	-0.108**	-0.002	-0.178***	-0.001	0.045
	(0.033)	(0.052)	(0.047)	(0.025)	(0.039)
% Special education	0.131	0.590	0.236	0.909***	0.195
	(0.191)	(0.358)	(0.173)	(0.219)	(0.173)
% English language learner	-0.075	-0.130	-0.146**	-0.090**	-0.093**
	(0.044)	(0.069)	(0.051)	(0.029)	(0.035)
SES composite score	0.053***	-0.008	0.018	-0.027	0.011
	(0.015)	(0.036)	(0.016)	(0.016)	(0.021)
School district FE	Yes	Yes	Yes	Yes	Yes
Year FE	Yes	Yes	Yes	Yes	Yes
Constant	-0.333***	-0.453***	-0.087*	-0.486***	-0.320***
	(0.032)	(0.067)	(0.036)	(0.039)	(0.034)
Adjusted R^2	0.880	0.837	0.879	0.758	0.771
N	5,494	2,953	7,128	5,694	4,547

Continued

S7 Table. Continued

C. Proportion of likely undocumented migrants	Q4 (top quartile)	Q3	Q2	Q1 (lowest quartile)
Secure Communities	-0.029***	0.007	0.009	-0.019
	(0.009)	(0.011)	(0.012)	(0.018)
**Controls for other policies**				
E-verify	omitted [a]	0.081***	0.070***	0.064**
		(0.015)	(0.019)	(0.020)
Omnibus Immigration Laws	0.032**	0.112***	0.091***	-0.081***
	(0.012)	(0.021)	(0.021)	(0.014)
287(g) state-level agreements	omitted [a]	-0.187***	omitted [a]	omitted [a]
		(0.018)		
287(g) county-level agreements	-0.028***	0.031	omitted [a]	0.004
	(0.008)	(0.022)		(0.023)
Sanctuary jurisdictions	0.018	-0.010	0.012	0.048
	(0.011)	(0.015)	(0.014)	(0.032)
**School district characteristics**				
% Free/reduced lunch	0.003	-0.127*	-0.074	0.011
	(0.026)	(0.050)	(0.045)	(0.060)
% Special education	0.446**	-0.010	0.044	-0.105
	(0.152)	(0.336)	(0.177)	(0.200)
% English language learner	-0.083***	-0.182	-0.247*	-0.235
	(0.024)	(0.199)	(0.123)	(0.227)
SES composite score	-0.001	0.017	0.070**	0.036
	(0.015)	(0.021)	(0.024)	(0.028)
School district FE	Yes	Yes	Yes	Yes
Year FE	Yes	Yes	Yes	Yes
Constant	-0.385***	-0.204***	-0.270***	-0.259***
	(0.026)	(0.054)	(0.036)	(0.049)
Adjusted R^2	0.884	0.852	0.844	0.839
N	14,870	5,365	6,942	2,558

Data from SEDA 2009-18, DHS, and CPS. Precision weighted estimates are based on Equation 1. Results are obtained using the method outlined by Sun and Abraham (2021). Clustered standard errors at the county level are in parentheses. Urban areas exclude sanctuary jurisdictions. [a] No policy identified in the states. * $p < 0.05$, ** $p < 0.01$, *** $p < 0.001$ (two-tailed)
